# Synthesis and structural properties of 2-((10-alkyl-10*H*-phenothiazin-3-yl)methylene)malononitrile derivatives; a combined experimental and theoretical insight

**DOI:** 10.1186/s13065-016-0158-z

**Published:** 2016-03-15

**Authors:** Fatimah Ali Al-Zahrani, Muhammad Nadeem Arshad, Abdullah M. Asiri, Tariq Mahmood, Mazhar Amjad Gilani, Reda M. El-shishtawy

**Affiliations:** Chemistry Department, Faculty of Science, King Abdulaziz University, P.O. Box 80203, Jeddah, 21589 Saudi Arabia; Centre of Excellence for Advanced Materials Research (CEAMR), King Abdulaziz University, P.O. Box 80203, Jeddah, 21589 Saudi Arabia; Department of Chemistry, COMSATS Institute of Information Technology, University Road, Tobe Camp, Abbottabad, 22060 Pakistan; Department of Chemistry, College of Science and Humanities, Prince Sattam bin Abdulaziz University, P.O. Box 83, Alkharj, 11942 Saudi Arabia; Department of Chemical Engineering, COMSATS Institute of Information Technology, Defence Road, Off Raiwind Road, Lahore, Pakistan

**Keywords:** Phenothiazine, X-ray, DFT, MEP, NBO, NLO

## Abstract

**Background:**

Donor acceptor moieties connected through π-conjugated bridges i.e. D-π-A, in order to facilitate the electron/charge transfer phenomenon, have wide range of applications. Many classes of organic compounds, such as cyanine, coumarin carbazole, indoline, perylene, phenothiazine, triphenylamine, tetrahydroquinoline and pyrrole can act as charge transfer materials. Phenothiazines have been extensively studied as electron donor candidates due to their potential applications as electrochemical, photovoltaic, photo-physical and DSSC materials.

**Results:**

Two phenothiazine derivatives, 2-((10-hexyl-10*H*-phenothiazin-3-yl)methylene)malononitrile (**3a**) and 2-((10-octyl-10*H*-phenothiazin-3-yl)methylene)malononitrile (**3b**) have been synthesized in good yields and characterized by various spectroscopic techniques like FT-IR, UV–vis, ^1^H-NMR, ^13^C-NMR, and finally confirmed by single crystal X-ray diffraction studies. Density functional theory (DFT) calculations have been performed to compare the theoretical results with the experimental and to probe structural properties. In order to investigate the excited state stabilities the absorption studies have been carried out experimentally as well as theoretically.

**Conclusions:**

Compound **3a** crystallises as monoclinic, P2 (1)/a and **3b** as P-1. The X-ray crystal structures reveal that asymmetric unit contains one independent molecule in **3a**, whereas **3b** exhibits a very interesting behavior in having a higher Z value of 8 and four independent molecules in its asymmetric unit. The molecular electrostatic potential (MEP) mapped over the entire stabilized geometries of the molecules indicates the potential sites for chemical reactivities. Furthermore, high first hyperpolarizability values entitle these compounds as potential candidates in photonic applications.

**Graphical abstract:**
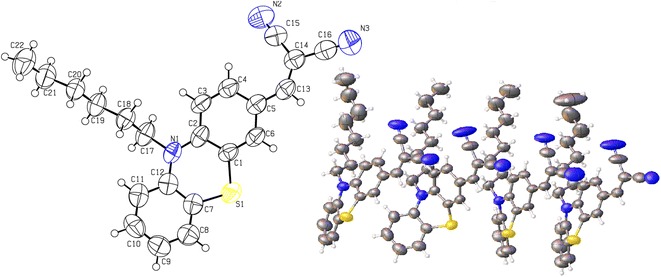
Phenothiazines; a comparison of experimental and theoretical analysis

**Electronic supplementary material:**

The online version of this article (doi:10.1186/s13065-016-0158-z) contains supplementary material, which is available to authorized users.

## Background

In few years, a great interest has developed in molecules having electron donor–acceptor (D–A) properties and their modern applications as dye sensitized solar cells (DSSC) [1], photosensitizers [2] and redox sensitizers [3]. The metal based donor–acceptor (D–A) complexes are well known where a metal atom behaves as an electron acceptor and ligands as electron donor species [4–6]. Ruthenium metal is a key contributor in the synthesis of such complexes. To avoid the cost of metal and its environmental hazards there is a space for the synthesis of new organic donor–acceptor molecules. A salient feature of such organic based (D–A) molecules is that donor acceptor moieties are connected through π-conjugated bridges i.e. D-π-A, in order to facilitate the electron/charge transfer phenomenon [7]. The classes of organic compounds that have been evaluated as (D–A) candidates include cyanine [8], coumarin [9], carbazole [10], indoline [11], perylene [12], phenothiazine [13], triphenylamine [14], tetrahydroquinoline [15] and pyrrole [16].

Molecules containing phenothiazine as electron donor part have been extensively studied due to their electrochemical [17], photovoltaic [18], photo-physical [19] and DSSC applications [1]. The synthesis of phenothiazine derivatives and their DSSC applications were claimed by many investigators, and the best results were produced in the solar cells where phenothiazine was used as electron donor and boradiazaindacene as electron acceptor candidates [19]. In addition to their physical applications, phenothiazine derivatives have been recognized as potent anti-psychotic [20], anti-infective [21], antioxidant, anti-cancer [22] and anti-Parkinson agents [23]. These were also qualified as valuable MALT1 protease [24], cholinesterase [25], and butyryl-cholinesterase enzyme inhibitors [26].

**Fig. 1:**
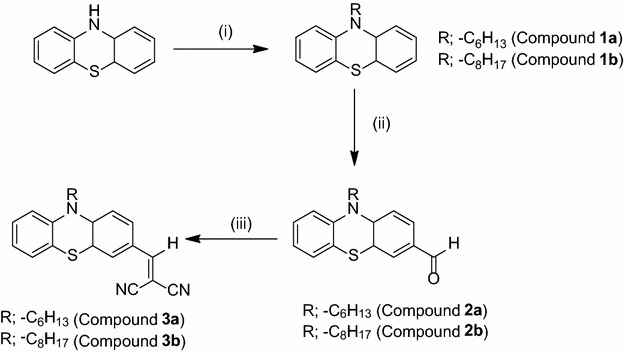
General synthetic scheme of title compounds **3a** and **3b**. *(i)* 1-Bromohexane (Compound **3a**), 1-Bromooctane (Compound **3b**), KOH, KI, DMSO; *(ii)* DMF, POCl3, 0 °C; *(iii)* Malonitrile, Piperidine, EtOH

In addition to our recent work [27–32], here we report the synthesis and structural properties of two new phenothiazine derivatives (Fig. 1). Both compounds have been synthesized in high yields and characterized by spectroscopic as well single crystal diffraction studies. The DFT investigations have been performed to validate the spectroscopic results, and to investigate other structural properties like frontier molecular orbital (FMO) analysis, molecular electrostatic potential (MEP), natural bond orbital (NBO) analysis (intra and inter molecular bonding and interaction among bonds), and first hyperpolarizability analysis (nonlinear optical response).

## Results and discussion

The synthesis of two phenothiazine derivatives **3a** and **3b** has been accomplished in three steps beginning from 10-phenothiazine resulting in good yields (details are given in the experimental section). These compounds have been characterized by ^1^H-NMR, ^13^C-NMR, FT-IR and UV–vis. spectroscopic techniques, and finally their structures have been confirmed by X-ray diffraction analysis. Computational studies have been carried out to compare the theoretically calculated spectroscopic properties with the experimental results, and to investigate some structural properties as well.

### X-ray diffraction analysis

**Fig. 2:**
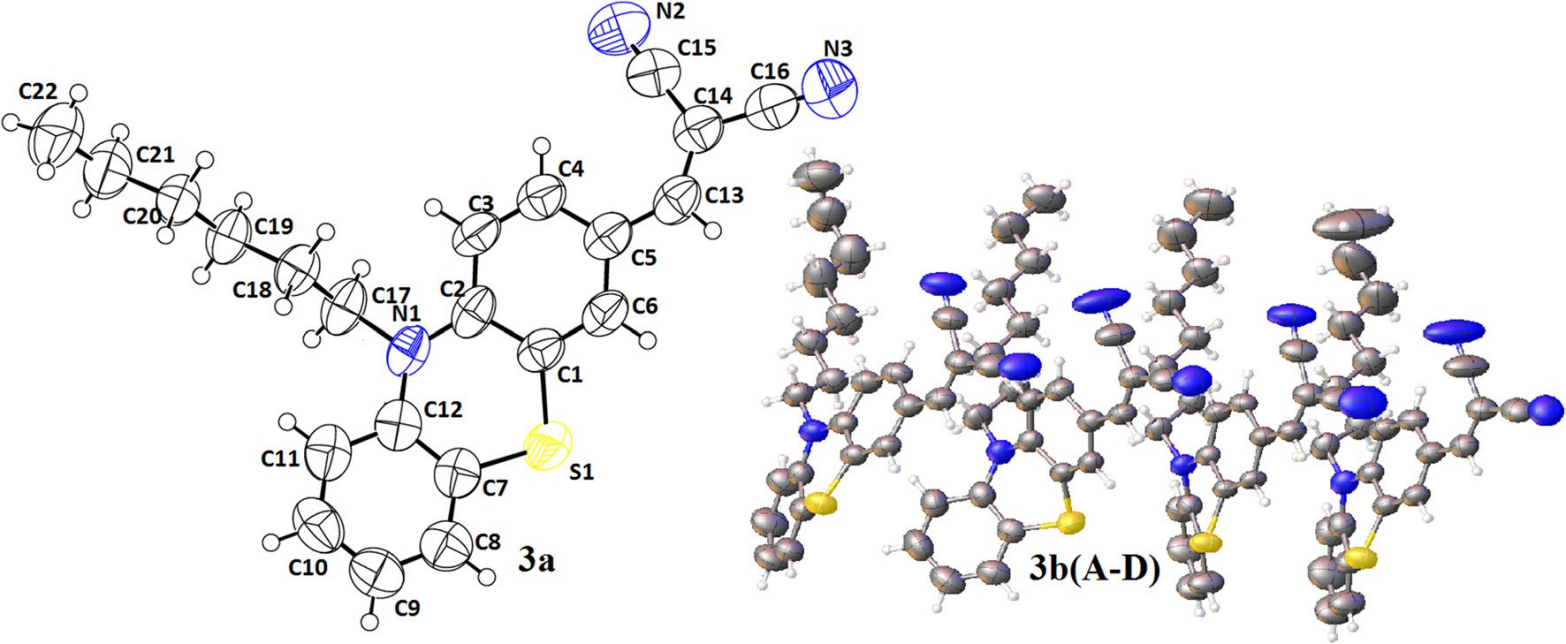
ORTEP diagram of **3a**, and **3b** containing four molecules (**A**, **B**, **C** and **D**) in an asymmetric unit, thermal ellipsoids were drawn at 50 % probability level

Both compounds **3a** and **3b** have been recrystallized in methanol under slow evaporation method in order to grow suitable crystals to ensure the final structures, and to study their three dimensional interactions. The compound **3a**, bearing a hexyl group at nitrogen, is crystallized in a monoclinic system having space group P2_1/a_ and **3b** containing an octyl substituent at nitrogen has been crystallized in a triclinic system having space group P-1. Complete crystal data parameters for both compounds have been provided in Table 1. The *ORTEP* views of both **3a** and **3b** are shown in Fig. 2.

**Table 1:** Crystal data and structure refinement parameters of **3a** and **3b**

Identification code	3a	3b
Empirical formula	C_22_H_21_N_3_S	C_24_H_25_N_3_S
Formula weight	359.48	387.53
Temperature/K	296.15	296.15
Crystal system	Monoclinic	Triclinic
Space group	P2_1_/a	P-1
a/Å	8.3072 (11)	16.4823 (7)
b/Å	13.5441 (19)	16.9423 (8)
c/Å	17.410 (2)	17.6368 (7)
α/°	90	106.027 (4)
β/°	92.275 (12)	110.499 (4)
γ/°	90	96.744 (4)
Volume/Å^3^	1957.3 (4)	4306.6 (3)
Z	4	8
Wave length Å	0.71073	0.71073
Diffraction radiation type	MoKα	MoKα
ρ_calc_mg/mm^3^	1.220	1.195
µ/mm^−1^	0.175	0.164
F (000)	760.0	1648.0
Crystal size/mm^3^	0.340 × 0.140 × 0.060	0.41 × 0.13 × 0.11
2θ range for data collection	5.756 to 59.036°	5.7 to 59.02°
Index ranges	−8 ≤ h ≤ 10, −17 ≤ k ≤ 17, −21 ≤ l ≤ 22	−21 ≤ h ≤ 22, −21 ≤ k ≤ 23, −23 ≤ l ≤ 24
Reflections collected	11,893	53,398
Independent reflections	4728 [R (int) = 0.0988]	20,881 [R (int) = 0.0574]
Data/restraints/parameters	4728/0/236	20,881/0/1013
Goodness-of-fit on F^2^	0.837	1.016
Final R indexes [I >=2σ (I)]	R_1_ = 0.0659, wR_2_ = 0.1162	R_1_ = 0.0752, wR_2_ = 0.1475
Final R indexes [all data]	R_1_ = 0.2559, wR_2_ = 0.1809	R_1_ = 0.2263, wR_2_ = 0.2183
Largest diff. peak/hole/e Å^−3^	0.18/−0.20	0.36/−0.29

**Fig. 3:**
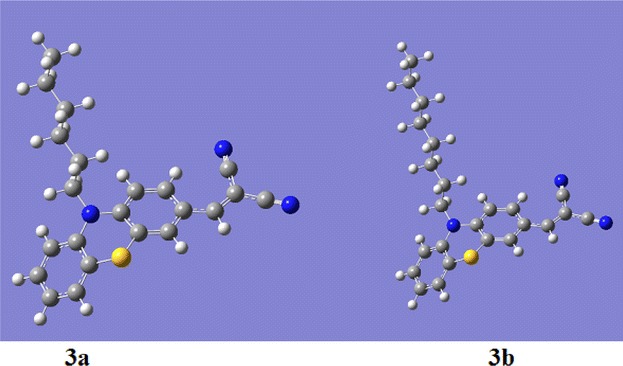
Optimized geometries of **3a**, **3b** at B3LYP/6-31G (d, p)

While analyzing the crystal structure it is observed that compound **3a** exists as single independent molecule in an asymmetric unit. On the other hand, an interesting behavior has been observed for **3b** which shows a high Z value of 8 and contains four independent molecules in its asymmetric unit (see Fig. 3) [C1–C24 molecule **A**, C25–C48 molecule **B**, C49–C72 molecule **C** and C73–C96 molecule **D**, (atomic labeling is in accordance with the compound **3a**, Fig. 2)].

The thiazine rings are not planar having the root mean square (rms) deviation values of 0.1721 (1) Å, 0.1841 (2) Å, 0.2184 (3) Å, 0.1392 (2) Å and 0.1593 (2) Å for compounds **3a** and **3b** (molecule **A**, molecule **B**, molecule **C**, molecule **D**) respectively. In compound **3a**, the two aromatic rings are oriented at a dihedral angle of 24.80(1)°, while the thiazine ring is oriented at dihedral angles of 13.33 (1)° and 12.56 (1)° with reference to ring 1 (C1–C6) and ring 2 (C7–C12), respectively.

In **3b**, having four molecules **A, B, C** and **D** in the asymmetric unit, the dihedral angles between the two aromatic rings are 24.85 (1)°, 32.41 (2)°, 18.83 (2)° and 23.80 (2)°. The observed orientation angles of thiazine rings with adjacent aromatic rings are 14.51 (2)°, 11.88 (2)° in molecule **A**, 16.28 (2)°, 16.49 (2)° in molecule **B**, 10.03(2)°, 10.16(2)° in molecule **C** and 13.63 (2)°, 11.74 (2)° in molecule **D**. These values are comparable with the already reported related structures [33–36], the difference is merely due to a variety of substituted groups on aromatic ring and nitrogen atom. The crystal structures revealed that the malononitrile group (NC–CH–CN) was not co-planar with the aromatic rings but was twisted at dihedral angles of 21.21 (2)°, 3.02 (5)°, 7.54 (5)°, 14.96 (4)° and 13.05 (5)° in **3a** and **3b** (A, B, C, D) respectively. The puckering parameters for molecule **3a** are Q_T_ = 0.424 Å, θ = 77.8 (5)° and φ = 4.1 (6)°, and in **3b** puckering parameters (Q_T_, θ and φ) are 0.4533 Å, 76.37°, 5.12 ° for molecule **A**, 0.5377 Å, 98.01°, 185.47° for molecule **B**, 0.3427 Å, 104.29°, 188.85° for molecule **C** and 0.3922 Å, 75.42°, 9.84° for molecule **D**. These values differentiate the four independent molecules in the asymmetric unit of crystal structure of compound **3b**, Additional file 1: Table S1. From the X-ray crystallographic studies, a weak C–H···N intermolecular interaction has been observed in **3a**. As a result of this interaction, a dimer is formed generating sixteen membered ring motifs *R*_1_^1^ (16) (see Additional file 1: Fig. S1). Molecules **A** and **B** in **3b** form dimers to generate sixteen membered ring motifs *R*_1_^1^ (16) Additional file 1: Fig. S2. The π-π interaction has not been observed either in **3a** or in **3b**.

### Geometry optimization

In the past decade, methods based on DFT have got the attention of researchers because of their accuracy and wide applications. The DFT investigations of both compounds **3a** and **3b** have been performed not only to validate X-ray results, but also to compare and investigate other spectroscopic and structural properties. The structures of both **3a** and **3b** have been optimized by using B3LYP/6-31G (d, p) level of theory, and the the optimized geometries are shown in Fig. 3. A comparison of bond angles and bond lengths for both compounds are listed in Additional file 1: Tables S2, S3. Although the packing diagram of **3b** shows four molecules in asymmetric unit, yet only molecule **A** has been considered for comparison. The experimental and simulated bond lengths/bond angles of all atoms for compounds **3a** and **3b (A)** are correlated nicely. A deviation of 0.001–0.036 Å in bond lengths has been appeared for both compounds. Maximum deviations of 5.4° and 4.2° in dihedral angles from C14–C13–C5 bonds in **3a** and from C23–C22–C21 bonds in **3b** have been observed.

### Vibrational analysis

**Fig. 4:**
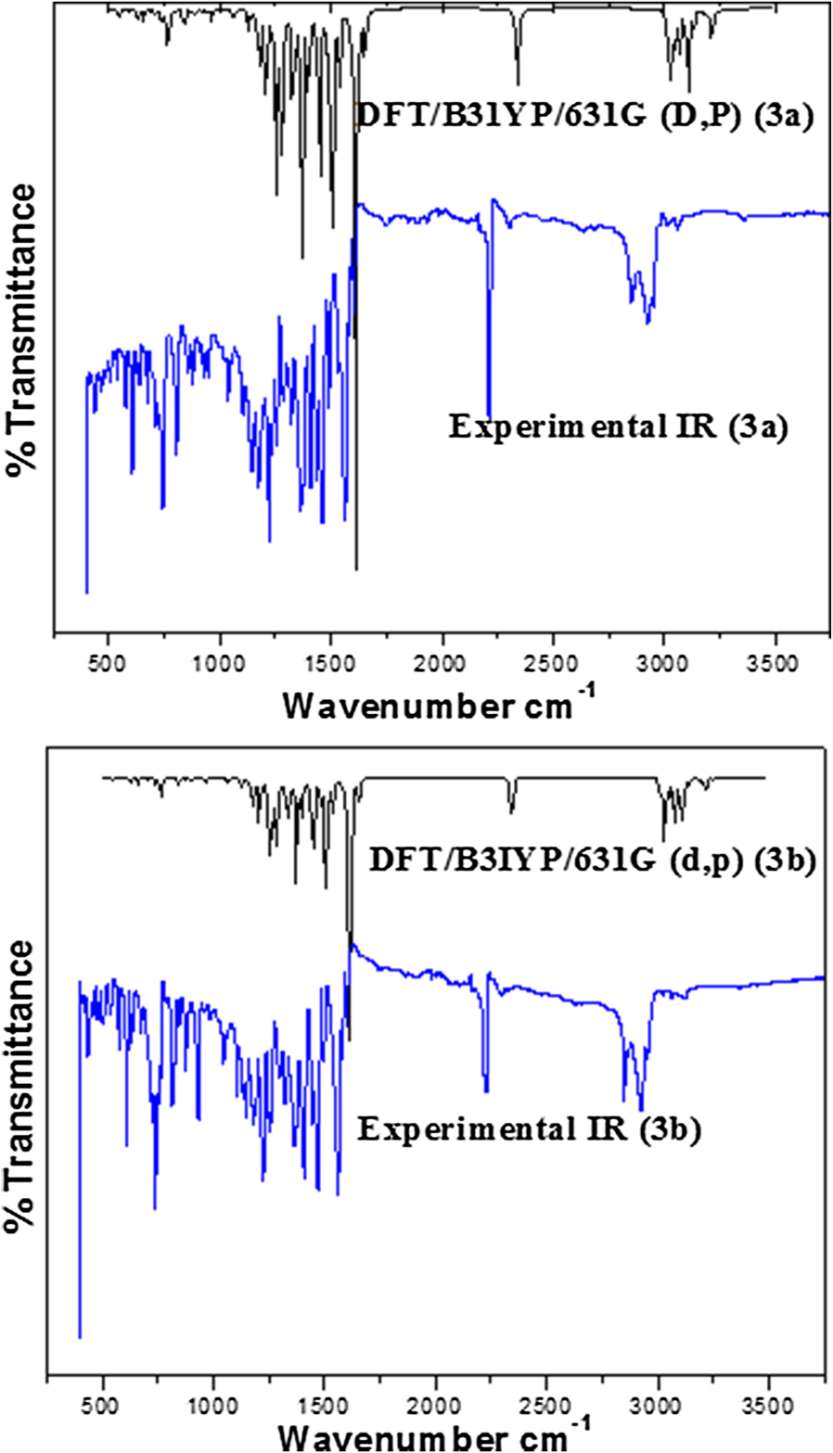
Experimental and simulated vibrational spectra of **3a** and **3b**

The experimental vibrational spectra of phenothiazine derivatives **3a** and **3b** have been recorded as neat, and both the experimental as well as simulated spectra are shown in Fig. 4. The vibrational frequencies of both were computed at the same level as was used for energy minima structures and assignments were accomplished by using Gauss-View 05 program. A comparison of experimental and calculated vibrational frequencies is given in Table 2.

**Table 2:** Experimental and simulated vibrational (cm^−1^) values of **3a** and **3b**

3a Calc. (intensity)	3a (Exp.)	Assignment	3b Calc. (intensity)	3b (Exp.)	Assignment
3086 (11.6)	–	υ_s_CH_arom._	3085 (13.1)	2916	υ_s_CH_arom._
3077 (21.9)	2916	υ_as_, υ_s_CH_arom._	3077 (21.2)	–	υ_as_CH_arom._
3001 (22.6)	–	υ_as_CH_2_	3005 (21.2)	–	υ_as_CH_2_
2986 (46.1)	–	υ_as_Me	2982 (42.8)	–	υ_as_Me
2980 (40.6)	–	υ_as_Me	2976 (59.1)	–	υ_as_Me,υ_s_CH_2_
			2965 (16.9)	–	υ_as_CH_2_
2966 (17.0)	–	υ_as_CH_2_	2954 (58.4)	2848	υ_as_CH_2_
2954 (58.6)	2848	υ_as_CH_2_	2945 (69.5)	–	υ_as_CH_2_
2936 (24.8)	–	υ_as_CH_2_	2923 (32.5)	–	υ_as_CH_2_
2926 (31.5)	–	υ_s_CH_2_, υ_as_CH_2_	2911 (35.6)	–	υ_s_Me
2914 (21.4)	–	υ_s_Me	2899 (80.5)	–	υ_s_CH_2_
2898 (43.2)	–	υ_s_CH_2_, υ_as_CH_2_	2893 (62.3)	–	υ_s_CH
2895 (48.8)	–	υ_s_CH_2_	2245 (119.0)	2215	υ_s_C≡N
2245 (119.1)	2214	υ_s_C≡N	2231 (13.9)	–	υ_as_C≡N
2230 (13.8)	–	υ_as_C≡N	1594 (64.5)	1570	υ_s_C=C_arom._
1603 (63.5)	1574	υ_s_C=C_arom._	1553 (579.0)	1559	υ_s_C=C_aliphatic_
1568 (10.9)	–	υ_s_C=C_arom._	1526 (18.4)	–	υ_as_C=C_arom._
1553 (578.2)	1559	υ_s_C=C_aliphatic_	1483 (61.2)	1461	υ_s_C–N–C
1526 (19.5)	–	υ_as_C=C_arom._			ρCH_2_
1483 (61.4)	1472	υ_s_C–N–C	1453 (112.5)	–	ρCH_2_
1456 (13.2)	–	ρCH_2_	1448 (189.8)	–	ρCH_2_
1453 (70.8)	–	ρCH_2_	1428 (41.4)	–	δCH_arom._
1448 (217.5)	1458	υ_as_C=C_arom._	1395 (230.2)	1405	υ_as_C=C_arom._
1428 (42.2)	–	βCH_arom._	1352 (23.2)	–	βCH
1395 (233.7)	1402	υ_as_C=C_arom._	1337 (206.7)	1364	υ_s_N–Ph,
1352 (21.6)	–	βCH			βCH_2_
1338 (189.1)	1360	υ_s_N–C, γCH_2_	1311 (24.2)	1323	βCH_2_, ωCH_2_
1337 (23.4)	–	βCH_2_	1303 (34.0)	–	βCH_2_, ωCH_2_
1312 (28.6)	–	βCH_2_	1294 (14.0)	–	υ_as_C=C_arom._
1300 (53.9)	–	βCH_2_	1290 (20.0)	–	ωCH_2_
1286 (98.9)	–	βCH_2_	1287 (87.5)	–	ωCH_2_
1279 (41.5)	–	υ_s_N–Ph	1279 (31.9)	–	υ_s_ CH_2_–N–Ph
1275 (27.8)	–	βCH_2_	1276 (39.2)	–	βCH_2_
1238 (97.0)	–	βCH_arom._	1238 (104.4)	1220	βCH_2_, υ_s_
1232 (90.2)	–	βCH_arom._			CH_2_–N–Ph
1208 (138.7)	1218	βCH_arom._			βCH_arom._
1206 (67.4)	–	βCH_2_	1233 (63.2)	–	υ_s_ CH_2_–N–Ph
1180 (22.7)	–	ωCH_2_	1212 (38.8)	–	γCH_2_
1163 (120.0)	–	γCH_arom._	1207 (168.5)	–	υ_s_C–C=CH
			1198 (27.7)	–	ωCH_2_
1133 (22.7)	–	υ_s_C–CN	1163 (121.8)	–	βCH_arom._
1127 (23.4)	–	ωCH_2_	1133 (23.1)		υ_as_C–CN
1119 (13.3)	–	βCH_arom._	1128 (24.0)	–	τCH_2_
1081 (15.0)	–	υ_s_C–S–C	1119 (13.1)	–	βCH_arom._
927 (10.9)	–	γCH	1083 (19.3)	–	υ_s_N–CH_2_
810 (22.3)	805	γCH_arom._	927 (10.6)	930	γCH
741 (26.2)	740	γPh	808 (22.6)	814	γCH_arom._
735 (27.2)	–	γCH_arom._	742 (10.3)	–	γCH_arom._
710 (17.5)	–	γPh	740 (15.2)	740	γCH_arom._
636 (12.4)	607	γC=C–CN			γCH_2_
429 (15.0)	–	γPh	734 (39.0)	–	γCHarom.
					βPh
			709 (12.2)	–	γPh
			588 (12.4)	–	γC=C–CN
			616 (10.0)	608	γPh
			429 (15.5)		γPh

Scaling factor used 0.958 for vibrations between 3200 and 1700 cm^−1^ and 0.9627 used below 1700 cm^−1^. Only those simulated values are given, those have shown intensity above 10

*υ*_*s*_ symmetric streching, *υ*_*as*_ asymmetric streching, *β* ın plane bending, γ out of plane bending, *τ* twisting, *ρ* scissoring, *ω* wagging

The simulated vibrations above 1700 cm^−1^ have been scaled by using a scaling factor of 0.958 and for less than 1700 cm^−1^ scaling factor is 0.9627 [37]. In the table only those simulated vibrations are given whose intensities are more than ten. For both compounds, the vibrations arise mainly from aromatic C–H, double bond C=C, C–N, C–S, nitrile, CH_2_, and CH_3_ functional groups. From Table 2, it is clear that there exists an excellent agreement between the experimental and theoretical vibrations.

### Aromatic (CH), (C=C) and aliphatic (C=C) vibrations

The aromatic (CH) vibrations generally appear in the region 2800–3100 cm^−1^ [38]. The bands appeared in this region are normally of very low intensity, and not much affected by substituents. In the simulated spectra, the aromatic CH stretching vibrations of both compounds **3a** and **3b** have been predicted at 3086, 3077 cm^−1^ and 3085, 3077 cm^−1^ respectively. The calculated aromatic CH stretching vibrations coincide well with the experimental value appearing at 2916 cm^−1^ for both compounds. The symmetric and asymmetric stretching vibrational regions of aromatic ring (C=C) usually lie in between 1600–1200 cm^−1^ [39]. The experimental scans of **3a** and **3b** show aromatic C=C stretching vibrations at 1574, 1402 cm^−1^ and 1570, 1405 cm^−1^ respectively. The simulated aromatic stretching C=C peaks are found in strong correlation and appear at 1603, 1568, 1526, 1395 cm^−1^ for compound **3a**, and 1594, 1526, 1395 cm^−1^ for compound **3b**. An aliphatic C=C group in conjugation with aromatic ring is also present in both compounds and appears at 1559 cm^−1^ experimentally whereas this stretching vibration appears at at 1553 cm^−1^ for both **3a** and **3b**.

Aromatic in-plane and out of plane CH bending vibrational regions are usually weak and are observed in the range 1000–1300 cm^−1^ and 650–900 cm^−1^ respectively [40]. In the simulated spectra, in plane CH (aromatic) bending vibrations are observed in the range of 1428–1286 cm^−1^ for compound **3a**, and in the region of 1352–1139 cm^−1^ for compound **3b**. The corresponding experimental values are depicted at 1218 cm^−1^ for compound **3a** and 1220 cm^−1^ for compound **3b**. The prominent out of plane CH (aromatic) bending vibrations of compound **3a** are observed at 1163, 927, 810 and 735 cm^−1^ in the simulated spectrum, and for compound **3b** these are observed in the range 927–740 cm^−1^. These out of plane bending vibrations are well supported by the experimental values of both compounds having their values noticed at 805 and 814 cm^−1^ respectively. The calculated out of plane bending vibrations of phenyl ring in compound **3a** are in the range 741–429 cm^−1^, and for **3b** in the range 709–429 cm^−1^. These simulated values are very nicely correlated with the experimental values of the both compounds.

### CH_2_ and CH_3_ group vibrations

The simulated stretching (symmetric/asymmetric) CH_2_ vibrations appear in the range of 3001–2895 cm^−1^, and 3005–2893 cm^−1^ for compounds **3a** and **3b** respectively. These simulated values appear in nice agreement with the experimental values having appeared at 2848 cm^−1^ for compound **3a**, and 2847 cm^−1^ for compound **3b**. Along with the stretching vibrations, several scissoring, in-plane and out of plane bending, methylene (CH_2_) and methyl vibrations are observed in the simulated and experimental spectra and a nice agreement is found between them.

Both compounds **3a** and **3b** show the CH_2_ scissoring vibrations in the range 1456–1448 cm^−1^ and 1453–1448 cm^−1^ respectively and these are correlated well with the experimental 1458 and 1462 cm^−1^ values respectively. The in-plane bending CH_2_ vibrations are observed in the range 1337–1275 cm^−1^ and 1337–1287 cm^−1^ for **3a** and **3b** respectively. These bending vibrations are in agreement with the experimental counterparts having appeared at 1317 cm^−1^, 1218 and 1323, 1228 cm^−1^ for **3a** and **3b** respectively.

### Nitrile and C–N Group vibrations

The nitrile symmetric stretching vibrations of very high intensity appear at 2245 cm^−1^ in the simulated spectra for **3a** and **3b**. The nitrile asymmetric stretching vibrations of low intensity also appear at 2230 and 2231 cm^−1^ for both compounds. In the experimental scans, the nitrile vibrations appear at 2214 and 2215 cm^−1^ for **3a** and **3b** respectively, and are found in excellent correlation with the simulated values. The simulated C–N–C stretching frequency appear at 1483 cm^−1^ for both **3a** and **3b** and is in full agreement with its experimental counterpart observed at 1472 and 1474 cm^−1^ respectively.

The assignments of N-Ph stretching modes are difficult, as there are problems to discriminate them from other aromatic ring vibrations. For substituted aromatic rings, Silverstein et al. [41] defined the N-Ph stretching bands in the range 1200–1400 cm^−1^. In the present study of compound **3a**, the observed N-Ph symmetric stretching bands appear at 1338 and 1279 cm^−1^ in the simulated spectrum and are in very good agreement with the experimental 1363 cm^−1^ value. Similarly, the calculated N-Ph stretching frequencies of **3b** appearing at 1337 and 1279 cm^−1^ also show good agreement with the experimental band at 1363 cm^−1^.

### Nuclear magnetic resonance (NMR) studies

For the last two to three decades, nuclear magnetic resonance spectroscopy has been unavoidable tool for structural investigations of organic and biological molecules. The ^1^H and ^13^C chemical shifts contain very important information about the structural environment of unknown compounds. Nowadays, a powerful method to predict and compare the structure of molecules is to combine the theoretical and experimental NMR methods. The DFT simulations using Gaussian software are playing very active role in this regard. A full and true geometry optimization of both compounds **3a** and **3b** has been performed by using B3LYP/6-311 + G (2d, p) basis set. An accurate optimization of molecular geometries is vital for reliable calculations of magnetic properties and their comparison with experimental results. The chemical shift calculations of both compounds have been performed by using the fully optimized geometries, adopting the GIAO method at the same level of theory and referred by using the internal reference standard i.e. trimethylsilane. Both the experimental as well as simulated NMR spectra have been recorded in CDCl_3_ (for experimental ^1^H and ^13^C NMR see Additional file 1: Figs. S3–S6). The detailed simulated and experimental ^1^H-NMR values are given in Table 3.

**Table 3:** Comparison of experimental and simulated ^1^H-NMR of **3a** and **3b** (ppm) in CDCl_3_

Proton (3a)	Exp.	Calc. (B3LYP)	Proton (3b)	Exp.	Calc. (B3LYP)
H_14_ (aromatic)	6.84	8.88	H_14_ (aromatic)	6.84	8.93
H_21_ (aliphatic)	7.47	7.68	H_21_ (aliphatic)	7.47	7.75
H_17_ (aromatic)	7.17	7.47	H_17_ (aromatic)	7.17	7.54
H_19_ (aromatic)	7.08	7.39	H_16_ (aromatic)	7.47	7.53
H_18_ (aromatic)	6.98	7.29	H_19_ (aromatic)	7.08	7.34
H_16_ (aromatic)	7.53	7.38	H_18_ (aromatic)	6.98	7.29
H_15_ (aromatic)	6.88	7.22	H_15_ (aromatic)	6.88	7.18
H_10_ (aromatic)	7.74	7.18	H_10_ (aromatic)	7.74	7.16
H_26_ (CH_2_)	3.87	4.24	H_26_ (CH_2_)	3.87	4.22
H_27_ (CH_2_)	3.87	3.77	H_27_ (CH_2_)	3.87	3.85
H_29_ (CH_2_)	1.81	2.04	H_29_ (CH_2_)	1.81	1.88
H_32_ (CH_2_)	1.81	1.87	H_32_ (CH_2_)	1.44	1.87
H_35_ (CH_2_)	1.44	1.94	H_35_ (CH_2_)	1.3	1.97
H_39_ (CH_2_)	1.32	1.67	H_30_ (CH_2_)	1.81	1.68
H_30_ (CH_2_)	1.81	1.61	H_39_ (CH_2_)	1.3	1.59
H_38_ (CH_2_)	1.32	1.23	H_41_ (CH_2_)	1.3	1.48
H_36_ (CH_2_)	1.44	1.11	H_48_ (CH_2_)	1.3	1.3
H_41_ (CH_3_)	0.88	1.09	H_36_ (CH_2_)	1.3	1.23
H_42_ (CH_3_)	0.88	1.01	H_49_ (CH_2_)	1.3	1.23
H_33_ (CH_2_)	1.81	1.07	H_38_ (CH_2_)	1.3	1.21
H_43_ (CH_3_)	0.88	0.55	H_51_ (CH_3_)	0.87	1.1
			H_33_ (CH_2_)	1.44	1.09
			H_42_ (CH_2_)	1.3	0.92
			H_52_ (CH_3_)	0.87	0.83
			H_53_ (CH_3_)	0.87	0.81

Both phenothiazine derivatives (**3a** and **3b**) mainly have aromatic and aliphatic protons. In the experimental ^1^H-NMR spectra, aromatic and double bonded protons appear in the range 7.74–6.83 ppm (compound **3a**) and 7.75–6.83 ppm (compound **3b**). The computed aromatic C–H signals (with respect to TMS) appear in the range 8.88–7.18 ppm (**3a**)/8.93–7.16 ppm (**3b**), and are found in nice agreement with the experimental values. The calculated chemical shift values for methylene and methyl hydrogen atoms of both **3a** and **3b** are found in the range 4.24–0.55/4.22–0.81 respectively, and are proved in good agreement with the experimental counterparts which appear in the range of 3.87–0.88 (**3a**)/3.87–0.87 (**3b**).

### Frontier molecular orbital analysis and UV–vis absorption studies

Frontier molecular orbital analysis has proved very helpful in understanding the electronic transitions within molecules and analyzing the electronic properties, UV–vis absorptions and chemical reactivity as well [42]. The FMO analysis also plays an important role in determining electronic properties such as ionization potential (I. P.) and electron affinity (E. A.). The HOMO (highest occupied molecular orbital) represents the ability to donate electrons and its energy corresponds to ionization potential (I. P.), whereas the LUMO (lowest unoccupied molecular orbital) acts as electron acceptor and its energy corresponds to electron affinity (E. A.) [43]. Frontier molecular orbital (FMO) analysis is carried out at the same level of theory as used for the geometry optimization, applying pop = full as an additional keyword. The HOMO and LUMO surfaces along with the corresponding energies and energy gaps are shown in Additional file 1: Fig. S6. Compound **3a** contains 93 filled orbitals, whereas **3b** contains 103 filled orbitals. The HOMO–LUMO energy difference in both **3a** and **3b** has been found to be 2.96 eV. The kinetic stabilities of compounds can be assigned on the basis of HOMO–LUMO energy gap [44]. A low HOMO–LUMO energy gap means less kinetic stability and high chemical reactivity. It is clear that the HOMO–LUMO energy gaps in compounds **3a** and **3b** are very less, indicating that electrons can easily be shifted from HOMO to LUMO after absorbing energy.

**Fig. 5:**
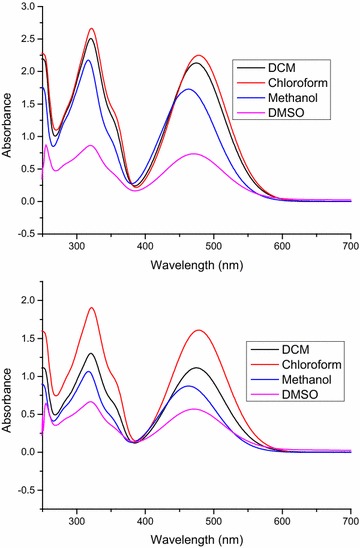
Combined experimental UV–vis. Spectra of **3a** (*above*), **3b** (*below*) in different solvents

The experimental UV–vis absorption spectra of both compounds **3a** and **3b** in various solvents like dichloromethane, chloroform, methanol and dimethyl sulphoxide (DMSO) have been recorded within 250–700 nm range, and the combined spectra are shown in (Fig. 5). The theoretical absorption studies are also carried out by using TD-DFT method at B3LYP/6-31G (d, p) level of theory in gas phase, and polarizable continuum model (PCM) is applied to account for solvent effect (For simulated UV–vis spectra see Additional file 1: Fig. S7). A comparison of characteristic experimental and simulated UV–vis. absorption wavelengths (λ_max_) of the both compounds in gas phase and different solvents (DCM, chloroform, methanol and DMSO) has been given in Table 4. As both the compounds have same chromophores; thus there is no significant difference in their absorption maxima.

**Table 4:** Experimental and simulated UV–vis. λ_max_ (nm) values of **3a** and **3b** measured in DCM, chloroform, methanol and DMSO

Experimental			Theoretical [TD-SCF/B3LYP/6-31G (d, p)]		
(3a)	λ_max1_ (abs.)	λ_max2_ (abs.)	(3b)	λ_max1_ (osc. strength)	λ_max2_ (osc. strength)
–	–	–	Gas Phase	300.4 (0.37)	476.4 (0.21)
DCM	320 (2.50)	474 (2.13)	DCM	310.4 (0.30)	502.9 (0.32)
Chloroform	321 (2.66)	478 (2.24	Chloroform	309 (0.29)	500.5 (0.32)
Methanol	317 (2.17)	478 (2.24)	Methanol	310.4 (0.35)	503.5 (0.30)
DMSO	319 (0.86)	472 (0.73)	DMSO	311.1 (0.28)	505.4 (0.32)
(3b)			(3b)		
–	–	–	Gas Phase	300.4 (0.36)	475.7 (0.21)
DCM	321 (1.30)	474 (1.11)	DCM	310.3 (0.28)	501.9 (0.32)
Chloroform	321 (1.90)	478 (1.61)	Chloroform	309.6 (0.28)	499.5 (0.32)
Methanol	317 (1.06)	463 (0.87)	Methanol	310.3 (0.34)	502.5 (0.31)
DMSO	320 (0.66)	473 (0.56)	DMSO	311.1 (0.26)	504.4 (0.32)

**Table 5:** Significant donor–acceptor interactions of **3a/3b** and their second order perturbation energies calculated at B3LYP level using 6-31G (d, p) basis set

Donor (i) (occupancy)	Type	ED_A_, % ED_B_, %	Acceptor (j) (occupancy)	Type	ED_A_, % ED_B_, %	E^(2)a^ (kcal/mol)	E_j_–E_i_^b^ (a.u.)	F (i, j) (a.u.)
BD C3–C4 1.97721	σ	49.64 50.36	BD* C2–C3 0.02660	σ*	51.36 48.64	3.16	1.25	0.056
BD C4–C5 1.97419	σ	48.62 51.38	BD* C3–C4 0.01233	σ*	50.36 49.64	2.53	1.29	0.051
BD C4–C5 1.59136	π	44.97 55.03	BD* C13–C14 0.24073	π*	59.29 40.71	22.02	0.27	0.071
BD C2–C3 1.97034	σ	48.64 51.36	BD* C3–C4 0.01233	σ*	50.36 49.64	2.69	1.30	0.053
BD C2–C3 1.60070	π	53.51 46.49	BD* C4–C5 0.42336	π*	55.03 44.97	25.19	0.28	0.076
BD C1–C2 1.97300	σ	50.22 49.78	BD* C2–C3 0.02660	σ*	51.36 48.64	3.44	1.26	0.059
BD C1–C6 1.97721	σ	50.97 49.03	BD* C5–C6 0.02189	σ*	50.82 49.18	2.95	1.27	0.055
BD C1–C6 1.71641	π	54.39 45.61	BD* C2–C3 0.40194	π*	46.49 53.51	19.81	0.29	0.069
BD C5–C6 1.97016	σ	49.18 50.82	BD* C4–C5 0.02494	σ*	51.38 48.62	3.18	1.24	0.056
BD C7–C12 1.97320	σ	49.77 50.23	BD* C11–C12 0.02533	σ*	48.64 51.36	3.93	1.28	0.063
BD C7–C8 1.97672	σ	51.41 48.59	BD* C7–C12 0.03387	σ*	50.23 49.77	4.58	1.26	0.068
BD C7–C8 1.69501	π	53.56 46.44	BD* C11–C12 0.38891	π*	51.02 48.98	20.16	0.28	0.069
BD C11–C12 1.66680	π	48.98 51.02	BD* C9–C10 0.33937	π*	50.66 49.34	20.47	0.29	0.069
BD C9–C10 1.66550	π	49.34 50.66	BD* C7–C8 0.38725	π*	46.44 53.56	22.74	0.27	0.071
BD C13–C14 1.81237	π	40.71 59.29	BD* C15–N2 0.08582	π*	54.47 45.53	19.91	0.39	0.081
BD C13–C14 1.81237	π	40.71 59.29	BD* C16–N3 0.08857	π*	54.71 45.29	20.52	0.40	0.083
LP N1 1.69519			BD* C2–C3 0.40194	π*	46.49 53.51	31.28	0.27	0.084
LP N1 1.69519			BD* C11–C12 0.38891	π*	51.02 48.98	24.09	0.28	0.075
LP S1 1.84528			BD* C1–C6 0.34392	π*	45.61 54.39	12.09	0.27	0.053
LP S1 1.84528			BD* C7–C8 0.38725	π*	46.44 53.56	11.23	0.27	0.053

^a^E^(2)^ means energy of hyperconjucative interactions (stabilization energy)

^b^Energy difference between donor (*i*) and acceptor (*j*) NBO orbitals

Different solvents covering a wide range of polarity and dielectric constant have been selected in order to explore the solvent effect on the absorption maxima, but no significant difference has been observed. The experimental UV–vis. spectra of both compounds show mainly two absorption bands. In dichloromethane, λ_max1_ and λ_max2_ values for compound **3a** appear at 320 and 474 nm corresponding to the π–π* and n–π* transitions respectively [45], and for **3b** the values appear at 321 nm and 474 nm. In chloroform the absorption maxima of **3a** are found at 321 nm (λ_max1_), 478 nm (λ_max2_) and for **3b** they have been appeared at 321 nm (λ_max1_), 478 (λ_max2_). Similarly, the absorption maxima values appear at 317 nm (λ_max1_), 478 nm for compound **3a**, and 317 nm (λ_max1_), 463 nm (λ_max2_), for compound **3b** in methanol (polar protic) and DMSO (polar aprotic) respectively. The gas phase simulated spectrum of compound **3a** show absorption maxima λ_max1_ and λ_max2_ at 300.4 nm (oscillating strength, f = 0.37) and 476.4 nm (f = 0.21) respectively. On the other hand, compound **3b** shows λ_max1_ at 300.4 nm (f = 0.36) and λ_max2_ at 475.7 nm (f = 0.21). The details of the simulated absorption values along with the oscillating strengths of both compounds in gas, dichloromethane (DCM), chloroform, methanol and DMSO are given in Table 4.

### Molecular electrostatic potential (MEP)

Molecular electrostatic potential (MEP) is associated with the electronic cloud. The electrophilic/nucleophilic reacting sites as well as hydrogen bonding interactions can be described in any compound on the basis of MEP [46, 47]. Recognition process of one molecule by another, as in drug-receptor and enzyme substrate interactions, is related to electrostatic potential V(r), because the two species show interaction to each other through their potentials. The MEP analysis can be performed by using the following mathematical relation, described previously [48].

$$V\left( r \right) = \sum {\frac{{Z_{A} }}{{\left| {R_{A} - \left. r \right|} \right.}}} - \int {\frac{{\rho \left( {r\prime } \right)}}{{\left| {r\prime - \left. r \right|} \right.}}} \;dr{\prime}$$

Here summation (Σ) runs over all nuclei *A* in a molecule, polarization and reorganization effects are ignored. *Z*_*A*_ is charge of nucleus *A*, located at *R*_*A*_ and *ρ* (*r*′) is the electron density function of a molecule. Usually, the preferred nucleophilic site is represented by red color and the preferred electrophilic site is represented by blue color. The electrostatic potential values at the surface are represented by different colors. The potential decreases in the order: red < orange < yellow < green < blue. The color code of the map is in the range between 0.0550 a.u. (deepest red) and 0.0550 a.u. (deepest blue), where blue corresponds to the strongest attraction and red corresponds to the strongest repulsion. Regions of negative *V (r)* are associated with lone pairs of electronegative atoms.

**Fig. 6:**
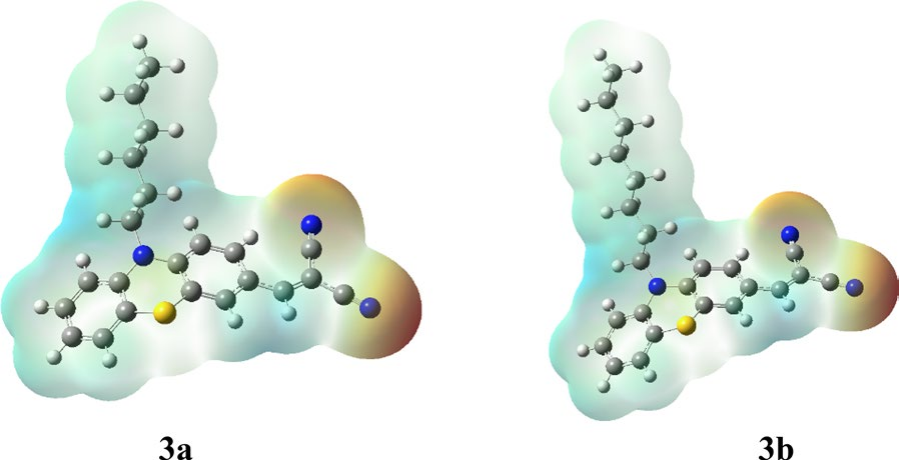
MEP plot of compounds **3a** and **3b**

According to the MEP analysis of compounds **3a** and **3b**, there are two negative regions at each molecule (red coded region) shown in Fig. 6. These red coded regions are nitrile functional groups of the both compounds. As these two compounds differ only at the alkyl chain lengths located at the nitrogen in a heterocyclic ring, therefore the reactive sites are same. Apart from the nitrile groups the rest is lying between yellow and green regions. This shows that no strong electrophilic sites exist in both the compounds.

### Natural bond orbital (NBO) analysis

Natural bond orbital analysis is an efficient method for studying intra- and intermolecular bonding and interaction among bonds, and provides a convenient basis to probe charge transfer or conjugative interaction [49]. The NBO approach describes the bonding anti-bonding interaction quantitatively and is expressed by means of second-order perturbation interaction energy E^(2)^ [50–53]. This energy estimates the off-diagonal NBO Fock matrix element. The stabilization energy E^(2)^ associated with *i* (donor) to *j* (acceptor) delocalization is approximated from the second-order perturbation approach as given below:

$$E^{\left( 2 \right)} = q_{i} \frac{{F^{2} \left( {i,j} \right)}}{{\varepsilon_{j} - \varepsilon_{i} }}$$

where *q*_*i*_ is the donor orbital occupancy, ε_*i*_ and ε_*j*_ are the diagonal elements (orbital energies) and *F* (*i, j*) is the off-diagonal Fock matrix element. The larger the *E*^(2)^ value is, the greater is the interaction between electron donors and electron acceptors and the extent of conjugation of whole system. The various second-order interactions between the occupied Lewis type (bond or line pair) NBO orbitals and unoccupied (anti-bonding and Rydberg) non-Lewis NBO orbitals are investigated by applying DFT at the B3LYP/6-31G (d, p) level. As a result of our study, the compounds **3a** and **3b** are types of Lewis structures with 97.93 and 98.03 % character, valance-non Lewis character of 1.90 and 1.79 % respectively. Both the compounds share the same Rydberg non-Lewis character of 0.16 %.

The intramolecular hyperconjugative interactions result in the transfer of charge from donor (π) to acceptor (π*) orbitals. This charge transfer increases the electron density (occupancy) in antibonding orbitals and weakens the respective bonds [54]. From the significant entries in Table 5, it is clear that the occupancy of π bonds (C–C) for benzene rings of the title compounds (**3a** and **3b**) lie in the range of ~1.59–1.71. On the other hand, the occupancy of π* bonds (C–C) for benzene rings range from ~0.33–0.42. This delocalization leads to the stabilized energy in the range of ~17.15–25.19 kcal/mol.

**Table 6:** First hyperpolarizability parameters of **3a** and **3b**

Compound	3a	3b
*β*_xxx_	−7021.88	1329.03
*β*_xxy_	1661.22	−2040.8
*β*_xyy_	−130.15	3129.93
*β*_yyy_	−267.26	−3673.5
*β*_xxz_	−103.44	−37.627
*β*_xyz_	47.6438	−20.756
*β*_yyz_	−95.5	−85.957
*β*_xzz_	105.388	−60.469
*β*_yzz_	−32.915	88.324
*β*_zzz_	−6.2876	−8.308
*β* × 10^−30 (esu)^	62.0307	61.7064

The pi-bond of ethylenic moiety (C13–C14) also shows an average of ~20 kcal/mol stabilization energy when it is delocalized to either acetonitrile group. The strongest stabilization energy to the system by 31.28 kcal/mol is due to the lone pair donation of nitrogen atom N (1) to the antibonding π* (C2–C3) orbital. On the other hand, the same lone pair gives a stabilization energy of 24.09 kcal/mol when it is conjugated with the antibonding π* (C11–C12) orbital of the aromatic ring. This clearly shows that the delocalization of lone pair of nitrogen N (1) is more towards that aromatic ring which has extended conjugation due to presence of electron withdrawing acetonitrile groups. The lone pair donation from sulfur atom (S1) to the antibonding π* (C1–C6) and (C7–C8) orbitals of both phenyl rings results in the stabilization energies of 12.09 and 11.23 kcal/mol respectively. The occupancy of lone pair electrons in sulfur atom (S1) is 1.84 as compared to 1.69 of lone pair on nitrogen atom (N1). As a consequence, the stabilization energies arising from the lone pair donation of sulfur atom to the antibonding π* (C–C) bonds of phenyl rings are comparatively smaller than those arising from lone pair donation of N1 atom. A plausible reason could be due to the deviation of sulfur atom from planarity because of its larger size. All σ to σ* transitions involving C–C bonds correspond to the weak stabilization energies in the range of ~2.53–4.58 kcal/mol.

### Hyperpolarizability and non-linear optical properties

Recently, compounds having non-linear optical (NLO) properties have got appreciable attention of researchers because of their wide applications in optoelectronic devices of telecommunications, information storage, optical switching and signal processing [55]. Molecules containing donor acceptor groups along with pi-electron conjugated system are considered as strong candidates for possessing NLO properties [56].

In each **3a** and **3b**, the phenothiazine moiety is connected to a nitrile group through a conjugated double bond, and these molecules are anticipated to show non-linear optical (NLO) properties. For the estimation of NLO properties, the first hyperpolarizability (*βo*) analysis for compounds **3a** and **3b** has been performed by employing same level of theory as for geometry optimization i.e. 6-31G (d, p) along with POLAR as an additional keyword. The first hyperpolarizability, a third rank tensor, is always described by a 3 × 3 × 3 matrix. The total 27 components of the 3D matrix can be reduced to 10 components as a result of Kleinman symmetry [57]. From the Gaussian output file ten components of 3D matrix have been identified as *β*_xxx_, *β*_xxy_, *β*_xyy_, *β*_yyy_, *β*_xxz_, *β*_xyz_, *β*_yyz_, *β*_xzz_, *β*_yzz_ and *β*_zzz_ respectively, and the values are given in Table 6.

Among all types of hyperpolarizabilities reported in literature, the more attractive is *β*_tot_. (First hyperpolarizability) [49] and it can be measured by using the following mathematical relation;

$$\beta = \sqrt {(\beta_{xxx} + \beta_{xyy} + \beta_{xzz} )^{2} + (\beta_{yyy} + \beta_{xxy} + \beta_{yzz} )^{2} + (\beta_{zzz} + \beta_{xxz} + \beta_{yyz} )^{2} }$$

First hyperpolarizability values have been converted into electrostatic units (1 a.u. = 8.6393 × 10^−33^esu). The calculated first hyperpolarizability (*β*_tot_.) values for **3a** and **3b** have been found to be 62.03 and 61.70 × 10^−30^esu respectively. These values are in excellent agreement with the reported values in literature [58, 59], and this agreement proves that both compounds are strong candidates for NLO applications.

## Method

All analytical grade chemicals and solvents were purchased from BDH, and used without further purification. Stuart Scientific (SMP3, version 5.0, UK) melting point apparatus was used to record the melting point, and the reported m. p. were uncorrected. ^1^H-NMR spectra were recorded on a Bruker-AVANCE-III 600 MHz at 300 K, and chemical shifts were reported in ppm with reference to the residual solvent signal. FT-IR spectra were recorded under neat conditions on Thermo Scientific NICOLET iS 50 FT-IR spectrometer (Thermo Scientific). UV–visible studies were performed by using Evolution 300UV/VIS spectrophotometer (Thermo Scientific).

### Crystallography

Sample crystals were mounted on Agilent Super Nova (Dual source) Agilent Technologies Diffractometer, equipped with graphite-monochromatic Cu/Mo Kα radiation source. The data collection was accomplished by using CrysAlisPro software [60] at 296 K. Structure solution was performed using SHELXS–97 and refined by full–matrix least–squares methods on F^2^ using SHELXL–97 [61], in-built with X-Seed [62]. All non–hydrogen atoms were refined anisotropically by full–matrix least squares methods [61]. All the C–H hydrogen atoms were positioned geometrically and treated as riding atoms with C–H = 0.93 Å and Uiso (H) = 1.2 Ueq (C) for aromatic carbon atoms. The methyl and methylene hydrogen atoms were also positioned geometrical with *C*_*methyl*_–H = 0.96 Å and *C*_*methylene*_–H = 0.97 Å and Uiso (H) = 1.5 Ueq (C) and Uiso (H) = 1.2 Ueq (C) for methyl and methylene carbon atoms respectively. The figures were drawn using ORTEP III [63], PLATON [64] and OLEX2 [65] programs. The cifs of both molecules have been assigned CCDC numbers 1028273 & 1028274 and these data files can be obtained free of charge on application to CCDC 12 Union Road, Cambridge CB21 EZ, UK. (Fax: (+44) 1223 336-033; e-mail: data_request@ccdc.cam. ac.uk).

### Computational details

Theoretical studies were performed by using Gaussian 09 software at density functional theory (DFT) level, as instituted in program [66]. The visualization of the results/optimized geometries was achieved by using Gauss view 05 [67]. The energy minima optimization of both compounds was carried out at B3LYP/6-31G (d, p) and B3LYP/6-311 + G (2d, p) levels of theory (the later was used further for nuclear magnetic studies). Frequency simulations were performed at the same level, to confirm the optimized geometries as a true minimum (no imaginary frequency). In addition, frequency simulations at B3LYP/6-311G (d, p) level were used for vibrational analysis. Nuclear magnetic resonance studies were performed at B3LYP/6-311 + G (2d, p) level, by adopting GIAO method in chloroform solvent and applying polarizable continuum model (PCM) for the solvent consideration. Chemical shift values were referred by using the internal reference standard i.e., tetramethylsilane. UV–vis absorption studies were simulated by using TD-DFT method and at B3LYP/6-31G (d, p) level of theory. MEP, NBO, FMO and first hyperpolarizability analyses were simulated at B3LYP/6-31G (d, p) level of DFT.

### Experimental

The synthesis of both phenothiazine derivatives was carried out in three steps starting from simple phenothiazine. First step was alkylation of nitrogen, followed by subsequent aldehyde formation and then conversion to final product (Fig. 1).

### General procedure for the synthesis of N-alkylated phenothiazine (1a, 1b)

In a round bottom flask a mixture of potassium hydroxide (2.003 g, 0.0357 mol), 10-phenothiazine (2.91 g, 0.0119 mol), 1-bromohexane (for **1a**) or 1-bromooctane (0.0179 mol) (for **1b**) and potassium iodide (in catalytic amount) in 50 ml dimethyl sulfoxide (DMSO) were taken. The reaction mixture was stirred for 5 h at room temperature and water (200 ml) was added. The crude product was extracted with CHCl_3_ (3 × 50 ml) and the organic layer was washed with saturated ammonium chloride solution and then with water. The organic layer was dried over anhydrous sodium sulfate and filtered, after removing the solvent under reduced pressure, crude product was purified by flash column chromatography (eluent: *n*-hexane) to obtain colorless oil **1a** in 88.68 % yield, and **1b** in 86.15 % yield.

### General procedure for synthesis of 10-alkyl-10*H* phenothiazine-3-carbaldehyde (2a, 2b)

To an ice cooled flask containing *N, N*-dimethylformamide (86 ml), POCl_3_ (53.5 ml) was added drop wise under stirring. After complete addition, the solution was stirred at room temperature for 90 min. Then the reaction mixture was cooled in an ice bath and already synthesized compound (**1a** or **1b)** (65 mmol) was added. The reaction mixture was warmed gradually up to 75 °C for 2 h. Then the mixture was cooled to room temperature and poured into ice water, basified (sat. aqueous K_2_CO_3_ solution) and extracted with CHCl_3_ (4 × 30 ml). Organic layer was washed, dried over MgSO_4_, filtered, evaporated and purified by flash silica gel column chromatography using petroleum ether/ethyl acetate (80/20) as eluent system to obtain yellow solids, **2a** in 92 % yield and **2b** in 91 % yield.

### Synthesis of 2-((10-hexyl-10*H*-phenothiazin-3-yl)methylene)malononitrile (3a) and 2-((10-octyl-10*H*-phenothiazin-3-yl)methylene)malononitrile (3b)

A mixture of **(2a** or **2b)** (3 mmol) and malononitrile (3 mmol) in basic ethanolic solution (10 ml) was stirred at room temperature overnight. The precipitates formed were filtered off and purified by recrystallization from methanol affording final products, **3a** in 78 % yield, and **3b** in 73 % yield.

### 2-((10-hexyl-10*H*-phenothiazin-3-yl)methylene)malononitrile (3a)

*M. p.* 84–85 °C* IR* (neat, cm^−1^): υ_max_ = 2916, 2848, 2214, 1559, 1472, 1458, 1402, 1360, 1218, 805, 740, 607; ^*1*^*H-NMR* (CDCl_3_, ppm): 7.74, (1H, dd, Ar–H, *J* = 1.8 Hz, 1.2 Hz), 7.53 (1H, d, Ar–H, *J* = 2.4 Hz), 7.47 (1H, s, Ar–H), 7.17 (1H, m, Ar–H), 7.08 (1H, dd, Ar–H, *J* = 1.8 Hz, 1.2 Hz), 6.98 (1H, m, Ar–H), 6.88 (1H, d, Ar–H, *J* = 8.4 Hz), 6.84 (2H, d, Ar–H, *J* = 9 Hz), 3.87 (2H, t, CH_2_, *J* = 7.2 Hz, 9.8 Hz), 1.44 (2H, pent, CH_2_), 1.32 (2H, pent, CH_2_), 1.81 (4H, pent, CH_2_), 0.88 (2H, t, CH_3_, *J* = 0.6 Hz, 1.2 Hz), ^*13*^*C-NMR* (CDCl_3_, ppm): 157.3, 150.8, 142.4, 131.4, 129.5, 127.8, 127.6, 125.1, 124.9, 122.9, 116.0, 114.8, 114.7, 113.6, 48.2, 31.3, 26.6, 26.4, 22.5, 14.0, UV–vis (DMSO): λ_max_ = 319.5 nm, 470.5 nm.

### 2-((10-octyl-10*H*-phenothiazin-3-yl)methylene)malononitrile (3b)

*M. p.* 90–92 °C *IR* (neat, cm^−1^): υ_max_ = 2916, 2848, 2215, 1570, 1559, 1461, 1405, 1364, 1220, 930, 814, 740, 608; ^*1*^*H-NMR* (CDCl_3_, ppm): 7.74 (1H, dd, Ar–H, *J* = 2.4 Hz, 1.8 Hz), 7.54 (1H, d, Ar–H, *J* = 2.4 Hz), 7.47 (1H, s, Ar–H), 7.17 (1H, m, Ar–H), 7.08 (1H, dd, Ar–H, *J* = 1.2 Hz, 1.2 Hz), 6.98 (1H, m, Ar–H), 6.88 (1H, d, Ar–H, *J* = 9 Hz), 6.84 (2H, d, Ar–H, *J* = 9 Hz), 3.88 (2H, t, CH_2_, *J* = 2.4 Hz, 1.8 Hz), 1.81 (2H, pent, CH_2_), 1.44 (2H, pent, CH_2_), 1.30 (8H, m, CH_2_), 0.87 (2H, t, CH_3_, *J* = 6.6 Hz, 7.2 Hz), ^*13*^*C-NMR* (CDCl_3_, ppm): 157.3, 150.8, 142.4, 131.4, 129.5, 127.8, 127.6, 125.1, 124. 9, 124.1, 122.9, 116.0, 114.9, 114.71, 113.5, 48.2, 31.7, 29.1, 29.1, 26.7, 26.6, 22.6, 14.1,* UV–vis.* (DMSO); λ_max_ = 320 nm, 471 nm.

## Conclusions

In this study, two novel phenothiazine derivatives 2-((10-hexyl-10*H*-phenothiazin-3-yl)methylene)malononitrile (**3a**) and 2-((10-octyl-10*H*-phenothiazin-3-yl)methylene)malononitrile (**3b**) have been synthesized and characterized by using FT-IR, UV–vis, ^1^H, ^13^C-NMR spectroscopic techniques and finally their structures are confirmed by single crystal X-ray diffraction studies. The DFT studies have shown a strong agreement between the simulated and experimental results. The optimized geometries of the both compounds at 6-31G (d, p) level have been used further for investigating structural properties. Frontier molecular orbital analysis shows that both the molecules have very low HOMO–LUMO energy gap, and therefore are kinetically less stable. The molecular electrostatic potential investigations reveal that electronegative region in both the compounds is spread over the nitrile groups. The high first hyperpolarizability values signify that these compounds can have very good nonlinear optical responses. The phenothiazine derivatives have very wide applications not only in dye sensitized solar cells but also in clinical field, and hopefully the results of this study will increase the interest of researchers working in this field.

## Additional file

10.1186/s13065-016-0158-z Cartesian co-ordinates of optimized geometries and cif files of **3a** and **3b** are given in supporting information. Experimental ^1^H,^13^C-NMR are also pasted in supporting information along with HOMO–LUMO surfaces, simulated UV–vis. Spectra and Tables containing bond length and bond angles data.
