# Supplementary material for: Synthesis and structural properties of 2-((10-alkyl-10H-phenothiazin-3-yl)methylene)malononitrile derivatives; a combined experimental and theoretical insight
Source: Chem Cent J. 2016 Mar 15;10:13. doi: 10.1186/s13065-016-0158-z (PMC4791767; doi:10.1186/s13065-016-0158-z)
Supplement: Supplementary file 1 — 10.1186/s13065-016-0158-z Cartesian co-ordinates of optimized geometries and cif files of 3a and 3b are given in supporting information. Experimental 1H,13C-NMR are also pasted in supporting information along with HOMO–LUMO surfaces, simulated UV–vis. Spectra and Tables containing bond length and bond angles data. [file 13065_2016_158_MOESM1_ESM.docx]

**Supplementary Inforamtion for**

**Synthesis and structural properties of 2-((10-alkyl-10H-phenothiazin-3-yl)methylene)malononitrile derivatives; A combined experimental and theoretical insight**

Fatimah Ali Al-Zahrani^a^, Muhammad Nadeem Arshad^a,b^*, Abdullah M. Asiri^a,b^, Tariq Mahmood^c^, Mazhar Amjad Gilani^d,e^, Reda M. El-shishtawy^a^

^a^Chemistry Department, Faculty of Science, King Abdulaziz University, P. O. Box 80203, Jeddah 21589, Saudi Arabia

^b^Centre of Excellence for Advanced Materials Research (CEAMR), King Abdulaziz University, P. O. Box 80203, Jeddah 21589, Saudi Arabia

^c^Department of Chemistry, COMSATS Institute of Information Technology, University Road, Tobe Camp, 22060 Abbottabad, Pakistan

^d^D epartment of Chemistry, College of Science and Humanities, Prince Sattam bin Abdulaziz University, P.O. Box 83, Alkharj 11942, Saudi Arabia

^e^Department of Chemical Engineering, COMSATS Institute of Information Technology, Defence Road, Off Raiwind Road, Lahore, Pakistan

*Corresponding author: E-mail: [mnachemist@hotmail.com](mailto:mnachemist@hotmail.com)

**Table S1; Showing the difference in puckering parameters**

| **Parameters** | **3a** | **3b** | | | |
| --- | --- | --- | --- | --- | --- |
|  |  | **Molecule A** | **Molecule B** | **Molecule C** | **Molecule D** |
| QT | 0.424Å | 0.4533 Å | 0.5377 Å | 0.3427 Å | 0.3922 Å |
| θ | 77.8o | 76.37 | 98.01 | 104.29 | 75.42 |
| φ | 4.1o | 5.12 | 185.47 | 188.85 | 9.84 |

**Table S2; Comparison of X-ray and simulated bond lengths (Å) of 3a and 3b(A)**

|  |  | **3a** |  |  | **3b(A)** |  |  |
| --- | --- | --- | --- | --- | --- | --- | --- |
| **Atom** | **Atom** | **X-ray/Å** | **Calc. B3LYP** | **Atom** | **Atom** | **X-ray/Å** | **Calc. B3LYP** |
| S1 | C1 | 1.759(4) | 1.781 | S1 | C1 | 1.757(3) | 1.781 |
| S1 | C7 | 1.757(4) | 1.782 | S1 | C7 | 1.750(3) | 1.782 |
| N1 | C2 | 1.394(5) | 1.398 | N1 | C2 | 1.389(4) | 1.398 |
| N1 | C12 | 1.413(5) | 1.423 | N1 | C12 | 1.418(4) | 1.423 |
| N1 | C17 | 1.480(5) | 1.471 | N1 | C17 | 1.474(4) | 1.471 |
| N2 | C15 | 1.138(6) | 1.164 | N2 | C15 | 1.131(5) | 1.164 |
| N3 | C16 | 1.128(5) | 1.164 | N3 | C16 | 1.137(5) | 1.164 |
| C1 | C2 | 1.415(5) | 1.414 | C1 | C2 | 1.412(4) | 1.414 |
| C1 | C6 | 1.380(5) | 1.385 | C1 | C6 | 1.370(4) | 1.385 |
| C2 | C3 | 1.405(5) | 1.410 | C2 | C3 | 1.399(4) | 1.410 |
| C3 | C4 | 1.371(5) | 1.385 | C3 | C4 | 1.366(4) | 1.385 |
| C4 | C5 | 1.413(5) | 1.411 | C4 | C5 | 1.402(4) | 1.411 |
| C5 | C6 | 1.396(5) | 1.414 | C5 | C6 | 1.394(4) | 1.414 |
| C5 | C13 | 1.442(5) | 1.443 | C5 | C13 | 1.432(4) | 1.443 |
| C7 | C8 | 1.381(6) | 1.395 | C7 | C8 | 1.381(5) | 1.395 |
| C7 | C12 | 1.389(6) | 1.406 | C7 | C12 | 1.391(4) | 1.406 |
| C8 | C9 | 1.373(6) | 1.394 | C8 | C9 | 1.374(5) | 1.394 |
| C9 | C10 | 1.366(6) | 1.393 | C9 | C10 | 1.368(6) | 1.393 |
| C10 | C11 | 1.374(6) | 1.395 | C10 | C11 | 1.377(5) | 1.395 |
| C11 | C12 | 1.390(6) | 1.402 | C11 | C12 | 1.390(4) | 1.402 |
| C13 | C14 | 1.347(6) | 1.371 | C13 | C14 | 1.360(5) | 1.371 |
| C14 | C15 | 1.435(7) | 1.430 | C14 | C15 | 1.423(6) | 1.430 |
| C14 | C16 | 1.440(6) | 1.433 | C14 | C16 | 1.428(6) | 1.433 |
| C17 | C18 | 1.532(5) | 1.534 | C17 | C18 | 1.523(4) | 1.534 |
| C18 | C19 | 1.511(5) | 1.543 | C18 | C19 | 1.510(4) | 1.543 |
| C19 | C20 | 1.504(5) | 1.533 | C19 | C20 | 1.504(4) | 1.533 |
| C20 | C21 | 1.507(5) | 1.533 | C20 | C21 | 1.490(5) | 1.533 |
| C21 | C22 | 1.504(6) | 1.531 | C22 | C23 | 1.471(6) | 1.531 |
|  |  |  |  | C22 | C21 | 1.499(5) | 1.533 |
|  |  |  |  | C23 | C24 | 1.464(6) | 1.531 |

**Table S3; Comparison of X-ray and simulated bond angles (˚) of 3a and 3b(A)**

| **Atom** | **Atom** | **Atom** | **X-ray**  **/˚** | **B3LYP** | **Atom** | **Atom** | **Atom** | **X-ray /˚** | **B3LYP** |
| --- | --- | --- | --- | --- | --- | --- | --- | --- | --- |
|  | **3a** |  |  |  |  | **3b(A)** |  |  |  |
| C7 | S1 | C1 | 99.9(2) | 97.8 | C7 | S1 | C1 | 99.5(16) | 97.8 |
| C2 | N1 | C12 | 122.9(4) | 118.9 | C2 | N1 | C12 | 121.8(3) | 118.9 |
| C2 | N1 | C17 | 116.6(4) | 119.6 | C2 | N1 | C17 | 118.6(3) | 119.6 |
| C12 | N1 | C17 | 119.4(4) | 119.4 | C12 | N1 | C17 | 118.3(3) | 119.4 |
| C2 | C1 | S1 | 121.2(4) | 119.0 | C2 | C1 | S1 | 119.8(2) | 118.9 |
| C6 | C1 | S1 | 117.6(3) | 120.2 | C6 | C1 | S1 | 118.9(2) | 120.3 |
| C6 | C1 | C2 | 121.0(4) | 120.5 | C6 | C1 | C2 | 120.9(3) | 120.5 |
| N1 | C2 | C1 | 120.1(4) | 119.9 | N1 | C2 | C1 | 121.5(3) | 119.9 |
| N1 | C2 | C3 | 123.3(4) | 122.2 | N1 | C2 | C3 | 122.4(3) | 122.2 |
| C3 | C2 | C1 | 116.6(4) | 117.8 | C3 | C2 | C1 | 116.2(3) | 117.8 |
| C4 | C3 | C2 | 122.2(4) | 121.5 | C4 | C3 | C2 | 122.6(3) | 121.5 |
| C3 | C4 | C5 | 120.9(5) | 120.9 | C3 | C4 | C5 | 121.0(3) | 120.9 |
| C4 | C5 | C13 | 123.1(4) | 125.5 | C4 | C5 | C13 | 126.1(3) | 125.5 |
| C6 | C5 | C4 | 117.3(4) | 117.4 | C6 | C5 | C4 | 116.8(3) | 117.4 |
| C6 | C5 | C13 | 119.6(4) | 117.0 | C6 | C5 | C13 | 117.0(3) | 117.0 |
| C1 | C6 | C5 | 121.7(4) | 121.7 | C1 | C6 | C5 | 122.3(3) | 121.7 |
| C8 | C7 | S1 | 118.3(4) | 120.1 | C8 | C7 | S1 | 118.2(3) | 120.1 |
| C8 | C7 | C12 | 120.4(4) | 120.7 | C8 | C7 | C12 | 120.4(3) | 120.7 |
| C9 | C8 | C7 | 121.6(5) | 120.3 | C12 | C7 | S1 | 121.1(3) | 119.0 |
| C10 | C9 | C8 | 117.9(5) | 119.3 | C9 | C8 | C7 | 121.3(4) | 120.3 |
| C9 | C10 | C11 | 121.8(5) | 120.5 | C10 | C9 | C8 | 118.4(4) | 119.3 |
| C10 | C11 | C12 | 120.7(5) | 120.6 | C9 | C10 | C11 | 121.3(4) | 120.4 |
| C7 | C12 | N1 | 121.3(4) | 119.4 | C10 | C11 | C12 | 120.8(4) | 120.6 |
| C7 | C12 | C11 | 117.6(5) | 118.2 | C7 | C12 | N1 | 121.1(3) | 119.9 |
| C11 | C12 | N1 | 121.0(5) | 121.7 | C11 | C12 | N1 | 121.2(3) | 121.7 |
| C14 | C13 | C5 | 130.9(5) | 125.5 | C11 | C12 | C7 | 117.7(3) | 118.2 |
| C13 | C14 | C15 | 124.4(5) | 125.1 | C14 | C13 | C5 | 132.2(3) | 131.5 |
| C13 | C14 | C16 | 120.2(5) | 119.2 | C13 | C14 | C15 | 125.2(4) | 125.1 |
| C15 | C14 | C16 | 115.3(5) | 115.6 | C13 | C14 | C16 | 119.9(4) | 119.2 |
| N2 | C15 | C14 | 178.0(7) | 179.3 | C15 | C14 | C16 | 114.9(3) | 115.6 |
| N3 | C16 | C14 | 179.6(6) | 179.6 | N2 | C15 | C14 | 178.0(5) | 179.3 |
| N1 | C17 | C18 | 117.0(3) | 112.9 | N3 | C16 | C14 | 179.4(5) | 179.7 |
| C19 | C18 | C17 | 109.5(3) | 112.0 | N1 | C17 | C18 | 115.6(2) | 112.9 |
| C20 | C19 | C18 | 114.1(3) | 113.0 | C19 | C18 | C17 | 112.3(3) | 111.9 |
| C19 | C20 | C21 | 114.8(4) | 113.5 | C20 | C19 | C18 | 113.8(3) | 113.0 |
| C22 | C21 | C20 | 114.3(4) | 113.1 | C21 | C20 | C19 | 116.7(3) | 113.4 |
|  |  |  |  |  | C23 | C22 | C21 | 117.7(4) | 113.5 |
|  |  |  |  |  | C24 | C23 | C22 | 117.0(4) | 113.2 |


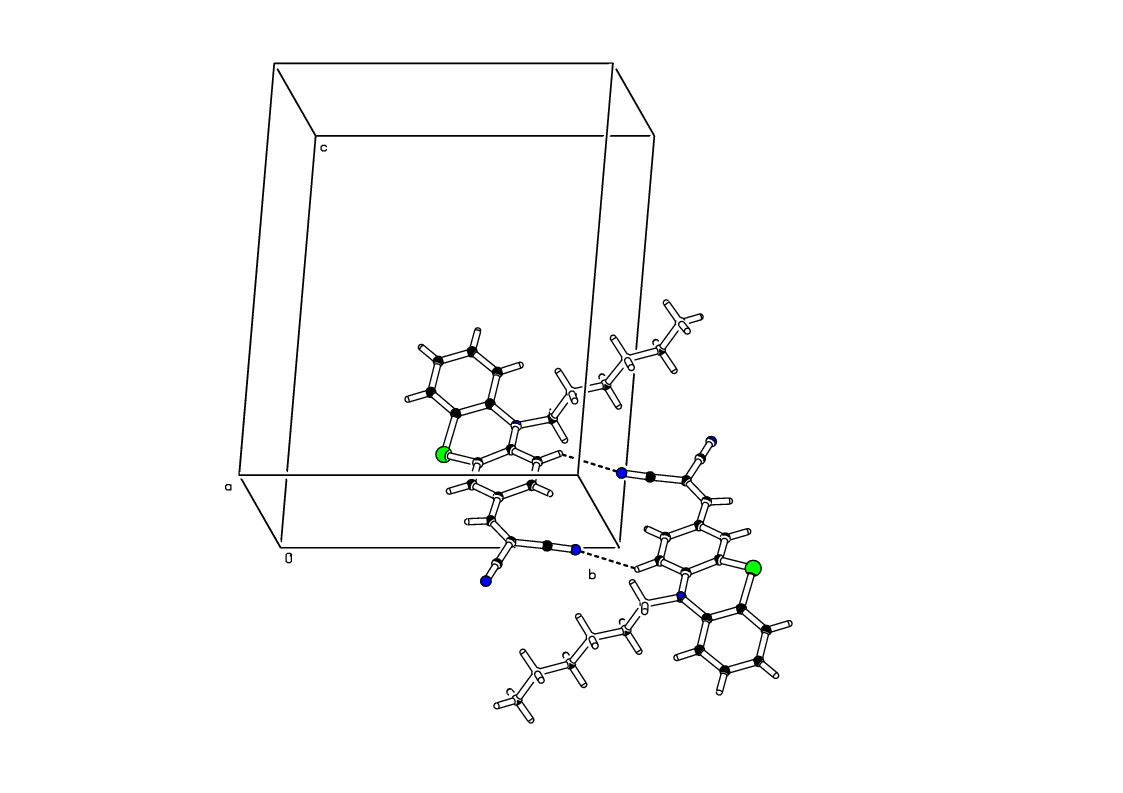


**Fig. S1;** Unit cell diagram of **(3a),** showing the dimers generated through hydrogen bonds.

**Fig. S2;** Experimental ^1^H-NMR spectrum of **3a** measured in CDCl_3_

**Fig. S3;** Experimental ^1^H-NMR spectrum of **3b** measured in CDCl_3_

**__**

**Fig. S4;** Experimental ^13^C-NMR of the **3a** measured in CDCl_3_

**__**

**Fig. S5;** Experimental ^13^C-NMR of **3b** measured in CDCl_3_

**Fig. S6;** FMO,s of **3a** and **3b**, showing the corresponding MOMO, LUMO and HOMO-LUMO energy gap.

**3a**

**3b**

**Fig. S7;** Combined simulated UV-vis. spectra measured at TD-B3LYP/6-31G (d, p) level in gas phase and in different solvents.

**Cif file of (3a)**

data_c:\nadeem

_audit_creation_method SHELXL-97

_chemical_name_systematic

;

2-(10-Hexyl-10H-phenothiazin-3-ylmethylene)-malononitrile

;

_chemical_name_common

;

2-(10-Hexyl-10H-phenothiazin-3-ylmethylene)-malononitrile

;

_chemical_melting_point ?

_chemical_formula_moiety 'C22 H21 N3 S'

_chemical_formula_sum

'C22 H21 N3 S'

_chemical_formula_weight 359.48

loop_

_atom_type_symbol

_atom_type_description

_atom_type_scat_dispersion_real

_atom_type_scat_dispersion_imag

_atom_type_scat_source

'C' 'C' 0.0181 0.0091

'International Tables Vol C Tables 4.2.6.8 and 6.1.1.4'

'H' 'H' 0.0000 0.0000

'International Tables Vol C Tables 4.2.6.8 and 6.1.1.4'

'N' 'N' 0.0311 0.0180

'International Tables Vol C Tables 4.2.6.8 and 6.1.1.4'

'S' 'S' 0.1246 0.1234

'International Tables Vol C Tables 4.2.6.8 and 6.1.1.4'

_symmetry_cell_setting 'Monoclinic'

_symmetry_space_group_name_H-M 'P 21/a'

_symmetry_space_group_name_Hall '-P 2yab'

loop_

_space_group_symop_operation_xyz

'x, y, z'

'-x+1/2, y+1/2, -z'

'-x, -y, -z'

'x-1/2, -y-1/2, z'

_cell_length_a 8.3072(11)

_cell_length_b 13.5441(19)

_cell_length_c 17.410(2)

_cell_angle_alpha 90

_cell_angle_beta 92.275(12)

_cell_angle_gamma 90

_cell_volume 1957.3(4)

_cell_formula_units_Z 4

_cell_measurement_temperature 296(2)

_cell_measurement_reflns_used ?

_cell_measurement_theta_min ?

_cell_measurement_theta_max ?

_exptl_crystal_description 'plate'

_exptl_crystal_colour 'red'

_exptl_crystal_size_max 0.34

_exptl_crystal_size_mid 0.14

_exptl_crystal_size_min 0.06

_exptl_crystal_density_meas ?

_exptl_crystal_density_diffrn 1.220

_exptl_crystal_density_method 'not measured'

_exptl_crystal_F_000 760

_exptl_absorpt_coefficient_mu 0.175

_exptl_absorpt_correction_T_min 0.76766

_exptl_absorpt_correction_T_max 1.00000

_exptl_absorpt_correction_type 'multi-scan'

_exptl_absorpt_process_details

;

CrysAlisPro, Agilent Technologies,

Version 1.171.36.20 (release 27-06-2012 CrysAlis171 .NET)

(compiled Jul 11 2012,15:38:31)

Empirical absorption correction using spherical harmonics,

implemented in SCALE3 ABSPACK scaling algorithm.

;

_exptl_special_details ?

_diffrn_ambient_temperature 296(2)

_diffrn_radiation_wavelength 0.71073

_diffrn_radiation_type MoK\a

_diffrn_radiation_source 'fine-focus sealed tube'

_diffrn_radiation_monochromator graphite

_diffrn_measurement_device_type 'SuperNova, Dual, Cu at zero, Atlas, CCD'

_diffrn_measurement_method '\w scans'

_diffrn_detector_area_resol_mean ?

_diffrn_standards_number 0

_diffrn_standards_interval_count .

_diffrn_standards_interval_time .

_diffrn_standards_decay_% ?

_diffrn_reflns_number 11893

_diffrn_reflns_av_unetI/netI 0.1799

_diffrn_reflns_av_R_equivalents 0.0988

_diffrn_reflns_limit_h_min -8

_diffrn_reflns_limit_h_max 10

_diffrn_reflns_limit_k_min -17

_diffrn_reflns_limit_k_max 17

_diffrn_reflns_limit_l_min -21

_diffrn_reflns_limit_l_max 22

_diffrn_reflns_theta_min 2.878

_diffrn_reflns_theta_max 29.518

_diffrn_reflns_theta_full 25.242

_diffrn_measured_fraction_theta_max 0.866

_diffrn_measured_fraction_theta_full 0.998

_diffrn_reflns_Laue_measured_fraction_max 0.866

_diffrn_reflns_Laue_measured_fraction_full 0.998

_diffrn_reflns_point_group_measured_fraction_max 0.866

_diffrn_reflns_point_group_measured_fraction_full 0.998

_reflns_number_total 4728

_reflns_number_gt 3183

_reflns_threshold_expression 'I > 2\s(I)'

_reflns_Friedel_coverage 0.000

_reflns_Friedel_fraction_max .

_reflns_Friedel_fraction_full .

_computing_data_collection 'CrysAlis PRO (Agilent, 2012)'

_computing_cell_refinement 'CrysAlis PRO (Agilent, 2012)'

_computing_data_reduction 'CrysAlis PRO (Agilent, 2012)'

_computing_structure_solution 'SHELXS-97 (Sheldrick, 1990)'

_computing_structure_refinement 'SHELXL-97 (Sheldrick, 1997)'

_computing_molecular_graphics ?

_computing_publication_material ?

_reflns_special_details

;

Reflections were merged by SHELXL according to the crystal

class for the calculation of statistics and refinement.

_reflns_Friedel_fraction is defined as the number of unique

Friedel pairs measured divided by the number that would be

possible theoretically, ignoring centric projections and

systematic absences.

;

_refine_ls_structure_factor_coef Fsqd

_refine_ls_matrix_type full

_refine_ls_weighting_scheme calc

_refine_ls_weighting_details

'w=1/[\s^2^(Fo^2^)+(0.0303P)^2^] where P=(Fo^2^+2Fc^2^)/3'

_atom_sites_solution_primary ?

_atom_sites_solution_secondary ?

_atom_sites_solution_hydrogens geom

_refine_ls_hydrogen_treatment constr

_refine_ls_extinction_method none

_refine_ls_extinction_coef .

_refine_ls_number_reflns 4728

_refine_ls_number_parameters 236

_refine_ls_number_restraints 0

_refine_ls_R_factor_all 0.2559

_refine_ls_R_factor_gt 0.0659

_refine_ls_wR_factor_ref 0.1809

_refine_ls_wR_factor_gt 0.1162

_refine_ls_goodness_of_fit_ref 0.837

_refine_ls_restrained_S_all 0.837

_refine_ls_shift/su_max 0.000

_refine_ls_shift/su_mean 0.000

loop_

_atom_site_label

_atom_site_type_symbol

_atom_site_fract_x

_atom_site_fract_y

_atom_site_fract_z

_atom_site_U_iso_or_equiv

_atom_site_adp_type

_atom_site_occupancy

_atom_site_site_symmetry_order

_atom_site_calc_flag

_atom_site_refinement_flags_posn

_atom_site_refinement_flags_adp

_atom_site_refinement_flags_occupancy

_atom_site_disorder_assembly

_atom_site_disorder_group

S1 S 0.42923(15) 0.51897(10) 0.15063(7) 0.0939(5) Uani 1 1 d . . . . .

N1 N 0.3983(4) 0.7211(3) 0.2258(2) 0.0705(10) Uani 1 1 d . . . . .

N2 N 0.6074(6) 0.9563(4) -0.1121(3) 0.1377(19) Uani 1 1 d . . . . .

N3 N 0.8773(5) 0.7381(4) -0.2359(3) 0.1220(17) Uani 1 1 d . . . . .

C1 C 0.5036(4) 0.6320(3) 0.1172(2) 0.0678(11) Uani 1 1 d . . . . .

C2 C 0.4706(4) 0.7219(3) 0.1549(3) 0.0697(12) Uani 1 1 d . . . . .

C3 C 0.5157(5) 0.8090(3) 0.1176(3) 0.0752(12) Uani 1 1 d . . . . .

H3 H 0.4965 0.8694 0.1409 0.090 Uiso 1 1 calc R U . . .

C4 C 0.5871(5) 0.8080(3) 0.0479(3) 0.0770(12) Uani 1 1 d . . . . .

H4 H 0.6102 0.8673 0.0239 0.092 Uiso 1 1 calc R U . . .

C5 C 0.6262(5) 0.7179(3) 0.0122(3) 0.0724(12) Uani 1 1 d . . . . .

C6 C 0.5856(4) 0.6309(3) 0.0498(2) 0.0722(12) Uani 1 1 d . . . . .

H6 H 0.6147 0.5706 0.0289 0.087 Uiso 1 1 calc R U . . .

C7 C 0.4281(4) 0.5441(4) 0.2496(3) 0.0737(12) Uani 1 1 d . . . . .

C8 C 0.4361(4) 0.4655(4) 0.3002(3) 0.0863(14) Uani 1 1 d . . . . .

H8 H 0.4509 0.4022 0.2811 0.104 Uiso 1 1 calc R U . . .

C9 C 0.4228(5) 0.4787(5) 0.3778(3) 0.0945(15) Uani 1 1 d . . . . .

H9 H 0.4262 0.4252 0.4114 0.113 Uiso 1 1 calc R U . . .

C10 C 0.4043(5) 0.5728(5) 0.4043(3) 0.0967(16) Uani 1 1 d . . . . .

H10 H 0.3962 0.5832 0.4568 0.116 Uiso 1 1 calc R U . . .

C11 C 0.3973(4) 0.6525(4) 0.3555(3) 0.0853(14) Uani 1 1 d . . . . .

H11 H 0.3852 0.7157 0.3753 0.102 Uiso 1 1 calc R U . . .

C12 C 0.4082(4) 0.6397(4) 0.2766(3) 0.0721(12) Uani 1 1 d . . . . .

C13 C 0.7069(4) 0.7126(3) -0.0595(3) 0.0750(12) Uani 1 1 d . . . . .

H13 H 0.7528 0.6516 -0.0696 0.090 Uiso 1 1 calc R U . . .

C14 C 0.7269(5) 0.7812(4) -0.1143(3) 0.0781(13) Uani 1 1 d . . . . .

C15 C 0.6624(6) 0.8794(4) -0.1123(3) 0.0946(15) Uani 1 1 d . . . . .

C16 C 0.8117(6) 0.7569(4) -0.1824(4) 0.0877(14) Uani 1 1 d . . . . .

C17 C 0.3373(4) 0.8168(3) 0.2536(2) 0.0802(13) Uani 1 1 d . . . . .

H17A H 0.2498 0.8035 0.2874 0.096 Uiso 1 1 calc R U . . .

H17B H 0.2923 0.8528 0.2096 0.096 Uiso 1 1 calc R U . . .

C18 C 0.4588(4) 0.8841(3) 0.2964(2) 0.0728(12) Uani 1 1 d . . . . .

H18A H 0.5143 0.8475 0.3374 0.087 Uiso 1 1 calc R U . . .

H18B H 0.5384 0.9073 0.2613 0.087 Uiso 1 1 calc R U . . .

C19 C 0.3716(4) 0.9711(3) 0.3297(2) 0.0795(13) Uani 1 1 d . . . . .

H19A H 0.3162 1.0066 0.2881 0.095 Uiso 1 1 calc R U . . .

H19B H 0.2907 0.9467 0.3635 0.095 Uiso 1 1 calc R U . . .

C20 C 0.4797(5) 1.0420(3) 0.3738(2) 0.0823(13) Uani 1 1 d . . . . .

H20A H 0.5574 1.0687 0.3391 0.099 Uiso 1 1 calc R U . . .

H20B H 0.5391 1.0057 0.4137 0.099 Uiso 1 1 calc R U . . .

C21 C 0.3938(5) 1.1268(4) 0.4105(3) 0.1060(17) Uani 1 1 d . . . . .

H21A H 0.3389 1.1652 0.3704 0.127 Uiso 1 1 calc R U . . .

H21B H 0.3125 1.1003 0.4432 0.127 Uiso 1 1 calc R U . . .

C22 C 0.5021(7) 1.1944(4) 0.4576(3) 0.144(2) Uani 1 1 d . . . . .

H22A H 0.5628 1.1564 0.4953 0.216 Uiso 1 1 calc R U . . .

H22B H 0.4381 1.2424 0.4831 0.216 Uiso 1 1 calc R U . . .

H22C H 0.5748 1.2274 0.4246 0.216 Uiso 1 1 calc R U . . .

loop_

_atom_site_aniso_label

_atom_site_aniso_U_11

_atom_site_aniso_U_22

_atom_site_aniso_U_33

_atom_site_aniso_U_23

_atom_site_aniso_U_13

_atom_site_aniso_U_12

S1 0.1272(10) 0.0697(9) 0.0838(9) -0.0123(7) -0.0058(7) -0.0163(7)

N1 0.067(2) 0.065(3) 0.078(3) -0.019(2) -0.0130(18) 0.0073(18)

N2 0.189(5) 0.087(4) 0.138(4) 0.017(4) 0.016(3) 0.029(3)

N3 0.134(4) 0.125(4) 0.108(4) -0.005(3) 0.021(3) -0.004(3)

C1 0.075(3) 0.055(3) 0.071(3) -0.002(2) -0.020(2) -0.002(2)

C2 0.063(2) 0.065(3) 0.079(3) -0.020(3) -0.024(2) 0.007(2)

C3 0.094(3) 0.049(3) 0.081(4) -0.004(3) -0.023(2) 0.005(2)

C4 0.091(3) 0.064(3) 0.074(3) -0.002(3) -0.022(2) -0.002(2)

C5 0.073(3) 0.064(3) 0.078(3) -0.004(3) -0.020(2) 0.002(2)

C6 0.086(3) 0.054(3) 0.075(3) -0.008(3) -0.019(2) 0.003(2)

C7 0.059(3) 0.081(4) 0.081(3) -0.010(3) -0.002(2) -0.005(2)

C8 0.071(3) 0.090(4) 0.099(4) -0.002(3) 0.008(3) -0.012(3)

C9 0.086(3) 0.103(5) 0.096(4) 0.010(4) 0.015(3) -0.008(3)

C10 0.087(3) 0.128(5) 0.076(4) 0.000(4) 0.014(3) -0.020(3)

C11 0.066(3) 0.099(4) 0.092(4) -0.021(3) 0.006(2) -0.003(2)

C12 0.052(2) 0.084(4) 0.080(3) -0.008(3) -0.003(2) 0.002(2)

C13 0.076(3) 0.065(3) 0.082(3) -0.013(3) -0.022(2) -0.003(2)

C14 0.080(3) 0.072(4) 0.081(4) -0.001(3) -0.015(2) -0.002(3)

C15 0.106(4) 0.080(4) 0.098(4) 0.006(4) 0.005(3) -0.001(3)

C16 0.094(4) 0.070(4) 0.098(4) 0.005(3) -0.006(3) -0.004(3)

C17 0.068(2) 0.077(3) 0.094(3) -0.025(3) -0.013(2) 0.014(2)

C18 0.058(2) 0.071(3) 0.089(3) -0.015(3) -0.001(2) 0.008(2)

C19 0.068(3) 0.080(3) 0.090(3) -0.020(3) -0.004(2) 0.010(2)

C20 0.088(3) 0.075(4) 0.084(3) -0.016(3) 0.007(2) 0.001(2)

C21 0.112(4) 0.097(4) 0.110(4) -0.027(4) 0.015(3) 0.001(3)

C22 0.188(6) 0.113(5) 0.130(5) -0.045(4) -0.014(4) -0.024(4)

_geom_special_details

;

All esds (except the esd in the dihedral angle between two l.s. planes)

are estimated using the full covariance matrix. The cell esds are taken

into account individually in the estimation of esds in distances, angles

and torsion angles; correlations between esds in cell parameters are only

used when they are defined by crystal symmetry. An approximate (isotropic)

treatment of cell esds is used for estimating esds involving l.s. planes.

;

loop_

_geom_bond_atom_site_label_1

_geom_bond_atom_site_label_2

_geom_bond_distance

_geom_bond_site_symmetry_2

_geom_bond_publ_flag

S1 C7 1.757(4) . ?

S1 C1 1.759(4) . ?

N1 C2 1.394(5) . ?

N1 C12 1.413(5) . ?

N1 C17 1.480(5) . ?

N2 C15 1.138(6) . ?

N3 C16 1.128(5) . ?

C1 C6 1.380(5) . ?

C1 C2 1.415(5) . ?

C2 C3 1.405(5) . ?

C3 C4 1.371(5) . ?

C3 H3 0.9300 . ?

C4 C5 1.413(5) . ?

C4 H4 0.9300 . ?

C5 C6 1.396(5) . ?

C5 C13 1.442(5) . ?

C6 H6 0.9300 . ?

C7 C8 1.381(6) . ?

C7 C12 1.389(6) . ?

C8 C9 1.373(6) . ?

C8 H8 0.9300 . ?

C9 C10 1.366(6) . ?

C9 H9 0.9300 . ?

C10 C11 1.374(6) . ?

C10 H10 0.9300 . ?

C11 C12 1.390(6) . ?

C11 H11 0.9300 . ?

C13 C14 1.347(6) . ?

C13 H13 0.9300 . ?

C14 C15 1.435(7) . ?

C14 C16 1.440(6) . ?

C17 C18 1.532(5) . ?

C17 H17A 0.9700 . ?

C17 H17B 0.9700 . ?

C18 C19 1.511(5) . ?

C18 H18A 0.9700 . ?

C18 H18B 0.9700 . ?

C19 C20 1.504(5) . ?

C19 H19A 0.9700 . ?

C19 H19B 0.9700 . ?

C20 C21 1.507(5) . ?

C20 H20A 0.9700 . ?

C20 H20B 0.9700 . ?

C21 C22 1.504(6) . ?

C21 H21A 0.9700 . ?

C21 H21B 0.9700 . ?

C22 H22A 0.9600 . ?

C22 H22B 0.9600 . ?

C22 H22C 0.9600 . ?

loop_

_geom_angle_atom_site_label_1

_geom_angle_atom_site_label_2

_geom_angle_atom_site_label_3

_geom_angle

_geom_angle_site_symmetry_1

_geom_angle_site_symmetry_3

_geom_angle_publ_flag

C7 S1 C1 99.9(2) . . ?

C2 N1 C12 122.9(4) . . ?

C2 N1 C17 116.6(4) . . ?

C12 N1 C17 119.4(4) . . ?

C6 C1 C2 121.0(4) . . ?

C6 C1 S1 117.6(3) . . ?

C2 C1 S1 121.2(4) . . ?

N1 C2 C3 123.3(4) . . ?

N1 C2 C1 120.1(4) . . ?

C3 C2 C1 116.6(4) . . ?

C4 C3 C2 122.2(4) . . ?

C4 C3 H3 118.9 . . ?

C2 C3 H3 118.9 . . ?

C3 C4 C5 120.9(5) . . ?

C3 C4 H4 119.6 . . ?

C5 C4 H4 119.6 . . ?

C6 C5 C4 117.3(4) . . ?

C6 C5 C13 119.6(4) . . ?

C4 C5 C13 123.1(4) . . ?

C1 C6 C5 121.7(4) . . ?

C1 C6 H6 119.1 . . ?

C5 C6 H6 119.1 . . ?

C8 C7 C12 120.4(4) . . ?

C8 C7 S1 118.3(4) . . ?

C12 C7 S1 121.2(4) . . ?

C9 C8 C7 121.6(5) . . ?

C9 C8 H8 119.2 . . ?

C7 C8 H8 119.2 . . ?

C10 C9 C8 117.9(5) . . ?

C10 C9 H9 121.1 . . ?

C8 C9 H9 121.1 . . ?

C9 C10 C11 121.8(5) . . ?

C9 C10 H10 119.1 . . ?

C11 C10 H10 119.1 . . ?

C10 C11 C12 120.7(5) . . ?

C10 C11 H11 119.7 . . ?

C12 C11 H11 119.7 . . ?

C7 C12 C11 117.6(5) . . ?

C7 C12 N1 121.3(4) . . ?

C11 C12 N1 121.0(5) . . ?

C14 C13 C5 130.9(5) . . ?

C14 C13 H13 114.6 . . ?

C5 C13 H13 114.6 . . ?

C13 C14 C15 124.4(5) . . ?

C13 C14 C16 120.2(5) . . ?

C15 C14 C16 115.3(5) . . ?

N2 C15 C14 178.0(7) . . ?

N3 C16 C14 179.6(6) . . ?

N1 C17 C18 117.0(3) . . ?

N1 C17 H17A 108.1 . . ?

C18 C17 H17A 108.1 . . ?

N1 C17 H17B 108.1 . . ?

C18 C17 H17B 108.1 . . ?

H17A C17 H17B 107.3 . . ?

C19 C18 C17 109.5(3) . . ?

C19 C18 H18A 109.8 . . ?

C17 C18 H18A 109.8 . . ?

C19 C18 H18B 109.8 . . ?

C17 C18 H18B 109.8 . . ?

H18A C18 H18B 108.2 . . ?

C20 C19 C18 114.1(3) . . ?

C20 C19 H19A 108.7 . . ?

C18 C19 H19A 108.7 . . ?

C20 C19 H19B 108.7 . . ?

C18 C19 H19B 108.7 . . ?

H19A C19 H19B 107.6 . . ?

C19 C20 C21 114.8(4) . . ?

C19 C20 H20A 108.6 . . ?

C21 C20 H20A 108.6 . . ?

C19 C20 H20B 108.6 . . ?

C21 C20 H20B 108.6 . . ?

H20A C20 H20B 107.5 . . ?

C22 C21 C20 114.3(4) . . ?

C22 C21 H21A 108.7 . . ?

C20 C21 H21A 108.7 . . ?

C22 C21 H21B 108.7 . . ?

C20 C21 H21B 108.7 . . ?

H21A C21 H21B 107.6 . . ?

C21 C22 H22A 109.5 . . ?

C21 C22 H22B 109.5 . . ?

H22A C22 H22B 109.5 . . ?

C21 C22 H22C 109.5 . . ?

H22A C22 H22C 109.5 . . ?

H22B C22 H22C 109.5 . . ?

loop_

_geom_torsion_atom_site_label_1

_geom_torsion_atom_site_label_2

_geom_torsion_atom_site_label_3

_geom_torsion_atom_site_label_4

_geom_torsion

_geom_torsion_site_symmetry_1

_geom_torsion_site_symmetry_2

_geom_torsion_site_symmetry_3

_geom_torsion_site_symmetry_4

_geom_torsion_publ_flag

C7 S1 C1 C6 153.1(3) . . . . ?

C7 S1 C1 C2 -31.3(3) . . . . ?

C12 N1 C2 C3 -155.0(4) . . . . ?

C17 N1 C2 C3 13.0(5) . . . . ?

C12 N1 C2 C1 24.4(5) . . . . ?

C17 N1 C2 C1 -167.7(3) . . . . ?

C6 C1 C2 N1 -175.2(3) . . . . ?

S1 C1 C2 N1 9.4(5) . . . . ?

C6 C1 C2 C3 4.2(5) . . . . ?

S1 C1 C2 C3 -171.2(3) . . . . ?

N1 C2 C3 C4 179.8(3) . . . . ?

C1 C2 C3 C4 0.4(5) . . . . ?

C2 C3 C4 C5 -3.3(5) . . . . ?

C3 C4 C5 C6 1.5(5) . . . . ?

C3 C4 C5 C13 -177.5(3) . . . . ?

C2 C1 C6 C5 -6.1(5) . . . . ?

S1 C1 C6 C5 169.5(3) . . . . ?

C4 C5 C6 C1 3.1(5) . . . . ?

C13 C5 C6 C1 -177.8(3) . . . . ?

C1 S1 C7 C8 -155.9(3) . . . . ?

C1 S1 C7 C12 28.6(4) . . . . ?

C12 C7 C8 C9 0.8(6) . . . . ?

S1 C7 C8 C9 -174.7(3) . . . . ?

C7 C8 C9 C10 -1.2(6) . . . . ?

C8 C9 C10 C11 0.7(7) . . . . ?

C9 C10 C11 C12 0.3(7) . . . . ?

C8 C7 C12 C11 0.2(6) . . . . ?

S1 C7 C12 C11 175.6(3) . . . . ?

C8 C7 C12 N1 -179.4(3) . . . . ?

S1 C7 C12 N1 -4.1(5) . . . . ?

C10 C11 C12 C7 -0.7(6) . . . . ?

C10 C11 C12 N1 178.9(4) . . . . ?

C2 N1 C12 C7 -27.5(5) . . . . ?

C17 N1 C12 C7 164.9(3) . . . . ?

C2 N1 C12 C11 152.9(3) . . . . ?

C17 N1 C12 C11 -14.7(5) . . . . ?

C6 C5 C13 C14 163.5(4) . . . . ?

C4 C5 C13 C14 -17.5(6) . . . . ?

C5 C13 C14 C15 -2.0(7) . . . . ?

C5 C13 C14 C16 -179.1(4) . . . . ?

C2 N1 C17 C18 -84.6(4) . . . . ?

C12 N1 C17 C18 83.8(5) . . . . ?

N1 C17 C18 C19 -172.3(4) . . . . ?

C17 C18 C19 C20 179.5(3) . . . . ?

C18 C19 C20 C21 -177.3(4) . . . . ?

C19 C20 C21 C22 177.2(4) . . . . ?

loop_

_geom_hbond_atom_site_label_D

_geom_hbond_atom_site_label_H

_geom_hbond_atom_site_label_A

_geom_hbond_distance_DH

_geom_hbond_distance_HA

_geom_hbond_distance_DA

_geom_hbond_angle_DHA

_geom_hbond_site_symmetry_A

C3 H3 N2 0.93 2.56 3.339(7) 142.1 3_675

C3 H3 N2 0.93 2.56 3.339(7) 142.1 3_675

_refine_diff_density_max 0.177

_refine_diff_density_min -0.200

_refine_diff_density_rms 0.042

**Cif file of (3b)**

data_c:\nadeem

_audit_creation_method SHELXL-97

_chemical_name_systematic

;

2-(10-Octyl-10<i>H<i/>-phenothiazin-3-ylmethylene)-malononitrile

;

_chemical_name_common

;

2-(10-Octyl-10<i>H<i/>-phenothiazin-3-ylmethylene)-malononitrile

;

_chemical_melting_point ?

_chemical_formula_moiety 'C24 H25 N3 S'

_chemical_formula_sum

'C24 H25 N3 S'

_chemical_formula_weight 387.53

loop_

_atom_type_symbol

_atom_type_description

_atom_type_scat_dispersion_real

_atom_type_scat_dispersion_imag

_atom_type_scat_source

'C' 'C' 0.0181 0.0091

'International Tables Vol C Tables 4.2.6.8 and 6.1.1.4'

'H' 'H' 0.0000 0.0000

'International Tables Vol C Tables 4.2.6.8 and 6.1.1.4'

'N' 'N' 0.0311 0.0180

'International Tables Vol C Tables 4.2.6.8 and 6.1.1.4'

'S' 'S' 0.1246 0.1234

'International Tables Vol C Tables 4.2.6.8 and 6.1.1.4'

_symmetry_cell_setting 'Triclinic'

_symmetry_space_group_name_H-M 'P -1'

_symmetry_space_group_name_Hall '-P 1'

loop_

_symmetry_equiv_pos_as_xyz

'x, y, z'

'-x, -y, -z'

_cell_length_a 16.4823(7)

_cell_length_b 16.9423(8)

_cell_length_c 17.6368(7)

_cell_angle_alpha 106.027(4)

_cell_angle_beta 110.499(4)

_cell_angle_gamma 96.744(4)

_cell_volume 4306.6(3)

_cell_formula_units_Z 8

_cell_measurement_temperature 296(2)

_cell_measurement_reflns_used 6760

_cell_measurement_theta_min 2.8458

_cell_measurement_theta_max 29.4470

_exptl_crystal_description 'needle'

_exptl_crystal_colour 'red'

_exptl_crystal_size_max 0.41

_exptl_crystal_size_mid 0.13

_exptl_crystal_size_min 0.11

_exptl_crystal_density_meas ?

_exptl_crystal_density_diffrn 1.195

_exptl_crystal_density_method 'not measured'

_exptl_crystal_F_000 1648

_exptl_absorpt_coefficient_mu 0.164

_exptl_absorpt_correction_T_min 0.76766

_exptl_absorpt_correction_T_max 1.00000

_exptl_absorpt_correction_type 'multi-scan'

_exptl_absorpt_process_details

;

CrysAlisPro, Agilent Technologies,

Version 1.171.36.20 (release 27-06-2012 CrysAlis171 .NET)

(compiled Jul 11 2012,15:38:31)

Empirical absorption correction using spherical harmonics,

implemented in SCALE3 ABSPACK scaling algorithm.

;

_exptl_special_details ?

_diffrn_ambient_temperature 296(2)

_diffrn_radiation_wavelength 0.71073

_diffrn_radiation_type MoK\a

_diffrn_radiation_source 'fine-focus sealed tube'

_diffrn_radiation_monochromator graphite

_diffrn_measurement_device_type 'SuperNova, Dual, Cu at zero, Atlas, CCD'

_diffrn_measurement_method '\w scans'

_diffrn_detector_area_resol_mean ?

_diffrn_standards_number 0

_diffrn_standards_interval_count .

_diffrn_standards_interval_time .

_diffrn_standards_decay_% ?

_diffrn_reflns_number 53398

_diffrn_reflns_av_R_equivalents 0.0574

_diffrn_reflns_av_sigmaI/netI 0.1264

_diffrn_reflns_limit_h_min -21

_diffrn_reflns_limit_h_max 22

_diffrn_reflns_limit_k_min -21

_diffrn_reflns_limit_k_max 23

_diffrn_reflns_limit_l_min -23

_diffrn_reflns_limit_l_max 24

_diffrn_reflns_theta_min 2.85

_diffrn_reflns_theta_max 29.51

_reflns_number_total 20881

_reflns_number_gt 7662

_reflns_threshold_expression >2sigma(I)

_computing_data_collection 'CrysAlis PRO (Agilent, 2012)'

_computing_cell_refinement 'CrysAlis PRO (Agilent, 2012)'

_computing_data_reduction 'CrysAlis PRO (Agilent, 2012)'

_computing_structure_solution 'SHELXS-97 (Sheldrick, 1990)'

_computing_structure_refinement 'SHELXL-97 (Sheldrick, 1997)'

_computing_molecular_graphics ?

_computing_publication_material ?

_iucr_refine_instructions_details

;

TITL 14089 in P-1

CELL 0.71073 16.4823 16.9423 17.6368 106.027 110.499 96.744

ZERR 8.0000 0.0007 0.0008 0.0007 0.004 0.004 0.004

LATT 1

SFAC C H N S

UNIT 192 200 24 8

MERG 2

FMAP 2

GRID

PLAN 20

TEMP 23

SIZE 0.11 0.13 0.41

BOND $H

HTAB

CONF

OMIT 3 3 9

OMIT 6 3 6

OMIT 7 0 7

OMIT 9 3 3

OMIT 6 0 6

OMIT -4 -4 4

L.S. 15

ACTA 52

WGHT 0.056900 0.552100

EXTI 0.000408

FVAR 1.36417

S1 4 0.929128 0.616699 0.455259 11.00000 0.07012 0.06198 =

0.08256 0.03294 0.05171 0.02991

S2 4 0.732626 0.684953 0.290205 11.00000 0.05720 0.07525 =

0.07516 0.02955 0.02973 0.01561

S3 4 0.372694 0.551802 -0.113931 11.00000 0.08366 0.07510 =

0.07018 0.01939 0.04868 0.02447

S4 4 1.147395 0.591946 0.668168 11.00000 0.08064 0.07293 =

0.08210 0.03139 0.05240 0.02887

N1 3 0.798406 0.704264 0.511357 11.00000 0.04851 0.05181 =

0.06043 0.01827 0.02747 0.02253

N2 3 0.699271 0.392471 0.690303 11.00000 0.13439 0.11370 =

0.18044 0.06195 0.12179 0.03844

N3 3 0.900680 0.253734 0.684517 11.00000 0.16652 0.14167 =

0.23201 0.13310 0.11734 0.08145

N4 3 0.544666 0.705231 0.247031 11.00000 0.06291 0.07031 =

0.06484 0.02538 0.02979 0.02779

N5 3 0.433966 0.393627 0.416182 11.00000 0.12949 0.16395 =

0.31032 0.13755 0.16225 0.07576

N6 3 0.652419 0.273240 0.451001 11.00000 0.10749 0.10524 =

0.15405 0.07761 0.06635 0.03783

N7 3 0.271538 0.665584 -0.030895 11.00000 0.06210 0.06409 =

0.06546 0.01664 0.03505 0.01777

N8 3 0.207486 0.391805 0.191330 11.00000 0.12918 0.12458 =

0.15982 0.06436 0.10781 0.04798

N9 3 0.409246 0.254327 0.197264 11.00000 0.11700 0.12923 =

0.18077 0.09742 0.08893 0.05223

N10 3 1.028693 0.692770 0.735464 11.00000 0.05866 0.05765 =

0.06374 0.01292 0.02751 0.02158

N11 3 0.931599 0.395136 0.922576 11.00000 0.18608 0.21034 =

0.34343 0.19055 0.21177 0.11487

N12 3 1.149730 0.276161 0.960661 11.00000 0.10210 0.10352 =

0.11515 0.05687 0.04663 0.02380

C1 1 0.875658 0.590193 0.518330 11.00000 0.04427 0.05050 =

0.05065 0.01588 0.02558 0.01625

C2 1 0.810488 0.632182 0.532624 11.00000 0.04278 0.04734 =

0.04800 0.00910 0.02168 0.01194

C3 1 0.758648 0.596438 0.568249 11.00000 0.04736 0.05381 =

0.06521 0.01332 0.03220 0.01714

AFIX 43

H3 2 0.712997 0.620603 0.575897 11.00000 -1.20000

AFIX 0

C4 1 0.772428 0.527446 0.592249 11.00000 0.05470 0.05460 =

0.06307 0.01521 0.03648 0.00972

AFIX 43

H4 2 0.735722 0.505451 0.614975 11.00000 -1.20000

AFIX 0

C5 1 0.841064 0.489275 0.583146 11.00000 0.04593 0.05604 =

0.05818 0.01981 0.02845 0.01414

C6 1 0.891415 0.522890 0.545329 11.00000 0.04354 0.05468 =

0.06170 0.02109 0.03080 0.02020

AFIX 43

H6 2 0.937282 0.498790 0.538186 11.00000 -1.20000

AFIX 0

C7 1 0.933305 0.725022 0.483505 11.00000 0.05564 0.05731 =

0.07090 0.02654 0.03183 0.01854

C8 1 0.998755 0.776888 0.475502 11.00000 0.08056 0.07245 =

0.11611 0.04907 0.05946 0.02572

AFIX 43

H8 2 1.042926 0.754654 0.462004 11.00000 -1.20000

AFIX 0

C9 1 0.999894 0.860695 0.487092 11.00000 0.10311 0.07257 =

0.15412 0.05681 0.07326 0.02102

AFIX 43

H9 2 1.043441 0.894906 0.480376 11.00000 -1.20000

AFIX 0

C10 1 0.935422 0.892586 0.508748 11.00000 0.10752 0.06082 =

0.11751 0.04362 0.05623 0.02576

AFIX 43

H10 2 0.935434 0.949216 0.516919 11.00000 -1.20000

AFIX 0

C11 1 0.870502 0.842445 0.518683 11.00000 0.08184 0.05873 =

0.08357 0.02646 0.04169 0.02823

AFIX 43

H11 2 0.828240 0.865974 0.534708 11.00000 -1.20000

AFIX 0

C12 1 0.867365 0.757145 0.505063 11.00000 0.05628 0.05120 =

0.05429 0.01834 0.02362 0.01549

C13 1 0.864595 0.419445 0.609513 11.00000 0.05573 0.06970 =

0.07459 0.03115 0.03674 0.02066

AFIX 43

H13 2 0.912042 0.402597 0.598072 11.00000 -1.20000

AFIX 0

C14 1 0.830731 0.373110 0.648096 11.00000 0.06985 0.06657 =

0.08117 0.03125 0.04228 0.01997

C15 1 0.757080 0.384681 0.670771 11.00000 0.09774 0.06907 =

0.10979 0.04130 0.06833 0.02425

C16 1 0.869410 0.306598 0.668596 11.00000 0.09981 0.09782 =

0.13488 0.07043 0.07277 0.03600

C17 1 0.719323 0.736019 0.513136 11.00000 0.05432 0.05876 =

0.06096 0.01608 0.02675 0.02848

AFIX 23

H17A 2 0.668893 0.687935 0.489996 11.00000 -1.20000

H17B 2 0.706245 0.768980 0.475149 11.00000 -1.20000

AFIX 0

C18 1 0.727387 0.790184 0.601350 11.00000 0.05774 0.05330 =

0.06279 0.00955 0.02634 0.02309

AFIX 23

H18A 2 0.734700 0.756166 0.638331 11.00000 -1.20000

H18B 2 0.780190 0.836243 0.626981 11.00000 -1.20000

AFIX 0

C19 1 0.646873 0.826178 0.596660 11.00000 0.06626 0.05703 =

0.06621 0.02037 0.03206 0.02986

AFIX 23

H19A 2 0.642108 0.863042 0.562670 11.00000 -1.20000

H19B 2 0.593798 0.780158 0.567080 11.00000 -1.20000

AFIX 0

C20 1 0.649667 0.875145 0.683163 11.00000 0.07130 0.06653 =

0.07455 0.01917 0.04123 0.02901

AFIX 23

H20A 2 0.656581 0.838340 0.717325 11.00000 -1.20000

H20B 2 0.702458 0.921459 0.711804 11.00000 -1.20000

AFIX 0

C22 1 0.573212 0.956068 0.770319 11.00000 0.10997 0.09105 =

0.09152 0.01975 0.05969 0.05111

AFIX 23

H22A 2 0.583176 0.918268 0.803378 11.00000 -1.20000

H22B 2 0.624363 1.004063 0.799354 11.00000 -1.20000

AFIX 0

C23 1 0.494355 0.987091 0.773481 11.00000 0.13483 0.14324 =

0.11530 0.03286 0.07292 0.07934

AFIX 23

H23A 2 0.486178 1.027306 0.743148 11.00000 -1.20000

H23B 2 0.442636 0.939716 0.741990 11.00000 -1.20000

AFIX 0

C24 1 0.495530 1.027487 0.858847 11.00000 0.17743 0.14718 =

0.15767 0.02668 0.11926 0.06915

AFIX 137

H24A 2 0.500295 0.987714 0.888897 11.00000 -1.50000

H24B 2 0.441412 1.045907 0.852813 11.00000 -1.50000

H24C 2 0.545655 1.075331 0.890814 11.00000 -1.50000

AFIX 0

C25 1 0.645300 0.618977 0.295676 11.00000 0.04555 0.06123 =

0.04745 0.01222 0.02345 0.01355

C26 1 0.562121 0.639821 0.278647 11.00000 0.05147 0.05808 =

0.05235 0.00931 0.02252 0.01725

C27 1 0.499680 0.592442 0.295491 11.00000 0.04306 0.07005 =

0.06880 0.01740 0.02550 0.01884

AFIX 43

H27 2 0.443887 0.604452 0.284248 11.00000 -1.20000

AFIX 0

C28 1 0.517568 0.529065 0.327897 11.00000 0.05071 0.06078 =

0.07006 0.01613 0.02905 0.01009

AFIX 43

H28 2 0.474920 0.500514 0.340427 11.00000 -1.20000

AFIX 0

C29 1 0.599781 0.506325 0.342618 11.00000 0.04998 0.05290 =

0.05821 0.01073 0.02660 0.01367

C30 1 0.661669 0.552647 0.324515 11.00000 0.04681 0.06089 =

0.05304 0.01496 0.02264 0.01840

AFIX 43

H30 2 0.715988 0.538174 0.332219 11.00000 -1.20000

AFIX 0

C31 1 0.669378 0.712946 0.202894 11.00000 0.07034 0.05211 =

0.05875 0.01093 0.02948 0.01186

C32 1 0.707806 0.731067 0.149726 11.00000 0.09162 0.07115 =

0.08568 0.02865 0.05089 0.02412

AFIX 43

H32 2 0.765410 0.724956 0.158551 11.00000 -1.20000

AFIX 0

C33 1 0.661607 0.758228 0.083428 11.00000 0.12175 0.07935 =

0.08022 0.03230 0.05408 0.02256

AFIX 43

H33 2 0.688784 0.773070 0.049548 11.00000 -1.20000

AFIX 0

C34 1 0.575179 0.762943 0.068411 11.00000 0.10594 0.08366 =

0.06186 0.02507 0.02811 0.01772

AFIX 43

H34 2 0.543219 0.779675 0.022973 11.00000 -1.20000

AFIX 0

C35 1 0.535179 0.743409 0.119247 11.00000 0.07812 0.07783 =

0.06340 0.02381 0.02246 0.02101

AFIX 43

H35 2 0.476025 0.745648 0.107116 11.00000 -1.20000

AFIX 0

C36 1 0.582667 0.720047 0.189472 11.00000 0.06695 0.05561 =

0.05472 0.01423 0.02250 0.01429

C37 1 0.624307 0.439891 0.374441 11.00000 0.04982 0.06379 =

0.06804 0.01965 0.03069 0.01491

AFIX 43

H37 2 0.679274 0.430476 0.376118 11.00000 -1.20000

AFIX 0

C38 1 0.582245 0.387979 0.402676 11.00000 0.06057 0.06470 =

0.07390 0.02170 0.03412 0.01659

C39 1 0.499565 0.391719 0.409449 11.00000 0.08354 0.09140 =

0.14644 0.06074 0.07685 0.03482

C40 1 0.621490 0.324028 0.429955 11.00000 0.07158 0.07570 =

0.09952 0.03765 0.04738 0.01615

C41 1 0.469964 0.741999 0.252483 11.00000 0.06718 0.07600 =

0.07827 0.02816 0.03248 0.03761

AFIX 23

H41A 2 0.415595 0.697472 0.223463 11.00000 -1.20000

H41B 2 0.463543 0.782318 0.222243 11.00000 -1.20000

AFIX 0

C42 1 0.480646 0.785997 0.343634 11.00000 0.06395 0.06362 =

0.07748 0.01654 0.03027 0.02427

AFIX 23

H42A 2 0.537021 0.827893 0.374322 11.00000 -1.20000

H42B 2 0.481889 0.744974 0.372778 11.00000 -1.20000

AFIX 0

C43 1 0.405393 0.828849 0.345836 11.00000 0.07050 0.06324 =

0.08783 0.02797 0.03723 0.02978

AFIX 23

H43A 2 0.405192 0.870156 0.317138 11.00000 -1.20000

H43B 2 0.349260 0.786762 0.313364 11.00000 -1.20000

AFIX 0

C44 1 0.410530 0.872500 0.434719 11.00000 0.07429 0.06848 =

0.09097 0.02615 0.04305 0.02897

AFIX 23

H44A 2 0.410206 0.830923 0.463037 11.00000 -1.20000

H44B 2 0.467125 0.913918 0.467277 11.00000 -1.20000

AFIX 0

C45 1 0.337125 0.916350 0.438593 11.00000 0.07689 0.08530 =

0.10208 0.03670 0.04671 0.04092

AFIX 23

H45A 2 0.338137 0.958694 0.411270 11.00000 -1.20000

H45B 2 0.280444 0.875181 0.405203 11.00000 -1.20000

AFIX 0

C46 1 0.341683 0.958475 0.527338 11.00000 0.08931 0.07734 =

0.10325 0.03448 0.05467 0.03619

AFIX 23

H46A 2 0.340785 0.915932 0.554440 11.00000 -1.20000

H46B 2 0.398613 0.999294 0.560589 11.00000 -1.20000

AFIX 0

C47 1 0.270217 1.002472 0.533274 11.00000 0.11130 0.13037 =

0.11377 0.03713 0.06132 0.07347

AFIX 23

H47A 2 0.213431 0.961196 0.502079 11.00000 -1.20000

H47B 2 0.269468 1.043455 0.504139 11.00000 -1.20000

AFIX 0

C48 1 0.277142 1.047162 0.621947 11.00000 0.13180 0.11242 =

0.13495 0.04321 0.08888 0.06753

AFIX 137

H48A 2 0.279962 1.007978 0.652545 11.00000 -1.50000

H48B 2 0.225825 1.069987 0.618310 11.00000 -1.50000

H48C 2 0.330088 1.092232 0.651954 11.00000 -1.50000

AFIX 0

C49 1 0.346882 0.549839 -0.025945 11.00000 0.05355 0.05756 =

0.05416 0.00985 0.02959 0.01072

C049 1 0.571280 0.910736 0.683501 11.00000 0.08357 0.08912 =

0.08272 0.02062 0.04549 0.03926

AFIX 23

H04A 2 0.518083 0.864879 0.652657 11.00000 -1.20000

H04B 2 0.565974 0.949696 0.651725 11.00000 -1.20000

AFIX 0

C50 1 0.293364 0.601353 0.000312 11.00000 0.05296 0.05817 =

0.05716 0.01089 0.02444 0.00592

C51 1 0.263324 0.583655 0.060607 11.00000 0.06709 0.06465 =

0.06991 0.01267 0.04122 0.01749

AFIX 43

H51 2 0.227577 0.615966 0.079096 11.00000 -1.20000

AFIX 0

C52 1 0.284490 0.521056 0.093031 11.00000 0.07127 0.06743 =

0.06789 0.01514 0.04279 0.01134

AFIX 43

H52 2 0.261330 0.510580 0.131348 11.00000 -1.20000

AFIX 0

C53 1 0.340418 0.472189 0.069807 11.00000 0.05867 0.05889 =

0.06342 0.01446 0.03200 0.01139

C54 1 0.371312 0.489949 0.010275 11.00000 0.05539 0.06062 =

0.06804 0.01332 0.03362 0.01483

AFIX 43

H54 2 0.410018 0.459782 -0.005256 11.00000 -1.20000

AFIX 0

C55 1 0.366488 0.654590 -0.112135 11.00000 0.05849 0.07400 =

0.05712 0.02127 0.02464 0.01409

C56 1 0.409310 0.689337 -0.154110 11.00000 0.07814 0.09197 =

0.07534 0.03465 0.03998 0.02120

AFIX 43

H56 2 0.442209 0.658650 -0.179055 11.00000 -1.20000

AFIX 0

C57 1 0.404824 0.767384 -0.160110 11.00000 0.11108 0.10669 =

0.10017 0.05517 0.05804 0.02796

AFIX 43

H57 2 0.433716 0.789777 -0.189012 11.00000 -1.20000

AFIX 0

C58 1 0.356440 0.812050 -0.122280 11.00000 0.12396 0.09427 =

0.11293 0.05597 0.06439 0.03795

AFIX 43

H58 2 0.351968 0.865075 -0.126331 11.00000 -1.20000

AFIX 0

C59 1 0.314775 0.779843 -0.078797 11.00000 0.09720 0.07588 =

0.08408 0.03180 0.04833 0.03237

AFIX 43

H59 2 0.284231 0.812321 -0.052161 11.00000 -1.20000

AFIX 0

C60 1 0.316958 0.699848 -0.073443 11.00000 0.06139 0.06616 =

0.05582 0.01655 0.02285 0.01557

C61 1 0.367435 0.406081 0.102258 11.00000 0.06451 0.06684 =

0.07401 0.01874 0.03637 0.01539

AFIX 43

H61 2 0.413789 0.387160 0.090235 11.00000 -1.20000

AFIX 0

C62 1 0.336424 0.367024 0.147347 11.00000 0.06867 0.06561 =

0.06991 0.02203 0.03261 0.01105

C63 1 0.264211 0.382100 0.171267 11.00000 0.08656 0.08089 =

0.09334 0.03291 0.05661 0.01938

C64 1 0.376860 0.304050 0.174075 11.00000 0.07932 0.09057 =

0.10918 0.04734 0.05358 0.01971

C65 1 0.203322 0.705915 -0.012508 11.00000 0.06095 0.07221 =

0.07100 0.01650 0.02995 0.02081

AFIX 23

H65A 2 0.157257 0.662616 -0.015247 11.00000 -1.20000

H65B 2 0.176062 0.728228 -0.057905 11.00000 -1.20000

AFIX 0

C66 1 0.234475 0.776864 0.073408 11.00000 0.07290 0.06682 =

0.07430 0.01605 0.03686 0.02227

AFIX 23

H66A 2 0.280518 0.821094 0.077499 11.00000 -1.20000

H66B 2 0.259763 0.755375 0.119943 11.00000 -1.20000

AFIX 0

C67 1 0.156444 0.813307 0.082151 11.00000 0.09353 0.07575 =

0.08729 0.02806 0.04967 0.03473

AFIX 23

H67A 2 0.139291 0.843460 0.041809 11.00000 -1.20000

H67B 2 0.106269 0.766554 0.064917 11.00000 -1.20000

AFIX 0

C68 1 0.171915 0.871001 0.168411 11.00000 0.12259 0.09524 =

0.09710 0.03179 0.05718 0.05275

AFIX 23

H68A 2 0.216960 0.921424 0.183261 11.00000 -1.20000

H68B 2 0.195017 0.843556 0.210286 11.00000 -1.20000

AFIX 0

C69 1 0.086800 0.897419 0.174631 11.00000 0.14063 0.11964 =

0.09080 0.01623 0.03541 0.07970

AFIX 23

H69A 2 0.064421 0.925166 0.132881 11.00000 -1.20000

H69B 2 0.041652 0.846589 0.158321 11.00000 -1.20000

AFIX 0

C70 1 0.097493 0.951425 0.255846 11.00000 0.15434 0.14287 =

0.12518 0.02785 0.04622 0.06214

AFIX 23

H70A 2 0.141580 1.002977 0.271955 11.00000 -1.20000

H70B 2 0.120448 0.924285 0.298037 11.00000 -1.20000

AFIX 0

C71 1 0.012323 0.974413 0.259278 11.00000 0.11989 0.16232 =

0.10969 0.01726 0.05281 0.06055

AFIX 23

H71A 2 0.028591 1.021615 0.312020 11.00000 -1.20000

H71B 2 -0.014084 0.994345 0.211768 11.00000 -1.20000

AFIX 0

C72 1 -0.057572 0.908030 0.255706 11.00000 0.14999 0.16634 =

0.17318 0.02715 0.10219 0.03660

AFIX 137

H72A 2 -0.085019 0.866877 0.198714 11.00000 -1.50000

H72B 2 -0.101873 0.933328 0.269885 11.00000 -1.50000

H72C 2 -0.031043 0.880939 0.296210 11.00000 -1.50000

AFIX 0

C73 1 1.104066 0.577440 0.742565 11.00000 0.04725 0.05905 =

0.05685 0.01406 0.02467 0.01286

C74 1 1.043062 0.622882 0.760259 11.00000 0.04900 0.05467 =

0.05528 0.01048 0.02286 0.01116

C75 1 0.997095 0.594492 0.805234 11.00000 0.05538 0.06936 =

0.07291 0.01472 0.03598 0.02086

AFIX 43

H75 2 0.953524 0.620891 0.814992 11.00000 -1.20000

AFIX 0

C76 1 1.014527 0.529431 0.834938 11.00000 0.06237 0.06500 =

0.07186 0.01779 0.03839 0.00925

AFIX 43

H76 2 0.982122 0.512179 0.863769 11.00000 -1.20000

AFIX 0

C77 1 1.080455 0.487792 0.822952 11.00000 0.05096 0.06003 =

0.06671 0.01712 0.03081 0.01294

C78 1 1.123824 0.514105 0.775489 11.00000 0.05188 0.06534 =

0.06446 0.01826 0.02812 0.01952

AFIX 43

H78 2 1.167523 0.487801 0.765914 11.00000 -1.20000

AFIX 0

C79 1 1.147748 0.698184 0.682861 11.00000 0.06180 0.07415 =

0.07861 0.03581 0.03315 0.01812

C80 1 1.205680 0.742829 0.660394 11.00000 0.09181 0.09440 =

0.12488 0.06115 0.06187 0.03467

AFIX 43

H80 2 1.245102 0.716442 0.641878 11.00000 -1.20000

AFIX 0

C81 1 1.206837 0.824983 0.664564 11.00000 0.11596 0.10828 =

0.16665 0.08587 0.07390 0.03377

AFIX 43

H81 2 1.244889 0.853666 0.647351 11.00000 -1.20000

AFIX 0

C82 1 1.150301 0.863722 0.694835 11.00000 0.13869 0.08657 =

0.14250 0.06035 0.06831 0.04127

AFIX 43

H82 2 1.150837 0.919746 0.699087 11.00000 -1.20000

AFIX 0

C83 1 1.092570 0.820886 0.719115 11.00000 0.09499 0.07780 =

0.09717 0.03749 0.04026 0.03077

AFIX 43

H83 2 1.055291 0.848602 0.740026 11.00000 -1.20000

AFIX 0

C84 1 1.089585 0.736740 0.712628 11.00000 0.07096 0.06312 =

0.06719 0.02474 0.02719 0.01988

C85 1 1.108289 0.424132 0.858444 11.00000 0.06045 0.06635 =

0.06808 0.02033 0.03060 0.01512

AFIX 43

H85 2 1.158553 0.409019 0.851971 11.00000 -1.20000

AFIX 0

C86 1 1.074201 0.382242 0.899684 11.00000 0.07047 0.06769 =

0.07700 0.02526 0.03788 0.01306

C87 1 0.995713 0.390934 0.912896 11.00000 0.11204 0.11244 =

0.17811 0.08694 0.10652 0.05129

C88 1 1.116519 0.322977 0.933240 11.00000 0.07659 0.07162 =

0.08395 0.02786 0.03941 0.00485

C89 1 0.955973 0.729902 0.746696 11.00000 0.06351 0.06410 =

0.06906 0.01040 0.02434 0.02504

AFIX 23

H89A 2 0.905611 0.684313 0.731991 11.00000 -1.20000

H89B 2 0.937580 0.759485 0.705973 11.00000 -1.20000

AFIX 0

C90 1 0.976964 0.790612 0.835748 11.00000 0.07382 0.06173 =

0.07411 0.01204 0.02979 0.02682

AFIX 23

H90A 2 1.027702 0.836290 0.851638 11.00000 -1.20000

H90B 2 0.993116 0.761243 0.876917 11.00000 -1.20000

AFIX 0

C91 1 0.898383 0.827150 0.839998 11.00000 0.07670 0.06304 =

0.07602 0.01817 0.03346 0.02917

AFIX 23

H91A 2 0.888273 0.862839 0.804658 11.00000 -1.20000

H91B 2 0.845766 0.780955 0.814861 11.00000 -1.20000

AFIX 0

C92 1 0.907777 0.877344 0.927333 11.00000 0.08894 0.07817 =

0.08214 0.02234 0.04246 0.03798

AFIX 23

H92A 2 0.959978 0.923936 0.952151 11.00000 -1.20000

H92B 2 0.918701 0.841858 0.962860 11.00000 -1.20000

AFIX 0

C93 1 0.829895 0.912615 0.931932 11.00000 0.10443 0.10589 =

0.09425 0.02760 0.05322 0.04959

AFIX 23

H93A 2 0.818748 0.947567 0.895860 11.00000 -1.20000

H93B 2 0.777881 0.865858 0.907311 11.00000 -1.20000

AFIX 0

C94 1 0.838146 0.963421 1.018402 11.00000 0.13864 0.18175 =

0.11690 0.03288 0.07211 0.08590

AFIX 23

H94A 2 0.890278 1.010061 1.043064 11.00000 -1.20000

H94B 2 0.849167 0.928399 1.054408 11.00000 -1.20000

AFIX 0

C95 1 0.759606 0.999390 1.023299 11.00000 0.20219 0.24027 =

0.13294 0.06851 0.10757 0.12962

AFIX 23

H95A 2 0.783109 1.054378 1.068397 11.00000 -1.20000

H95B 2 0.730227 1.008835 0.969490 11.00000 -1.20000

AFIX 0

C96 1 0.701345 0.958615 1.036848 11.00000 0.28774 0.25667 =

0.58721 0.28872 0.33862 0.15952

AFIX 137

H96A 2 0.688177 0.899843 1.003424 11.00000 -1.50000

H96B 2 0.648248 0.979248 1.020654 11.00000 -1.50000

H96C 2 0.722722 0.965337 1.096904 11.00000 -1.50000

AFIX 0

HKLF 4

REM 14089 in P-1

REM R1 = 0.0750 for 7662 Fo > 4sig(Fo) and 0.2260 for all 20881 data

REM 1014 parameters refined using 0 restraints

END

WGHT 0.0568 0.5537

REM Instructions for potential hydrogen bonds

EQIV $1 -x+1, -y+1, -z+1

HTAB C3 N5_$1

HTAB C27 N2_$1

HTAB C51 N11_$1

HTAB C75 N8_$1

REM Highest difference peak 0.370, deepest hole -0.282, 1-sigma level 0.041

Q1 1 0.0550 0.8837 0.2178 11.00000 0.05 0.37

Q2 1 0.1580 0.9469 0.2192 11.00000 0.05 0.36

Q3 1 0.7632 1.0198 1.1020 11.00000 0.05 0.26

Q4 1 0.9033 0.3743 0.8578 11.00000 0.05 0.22

Q5 1 0.6563 0.3692 0.6206 11.00000 0.05 0.22

Q6 1 0.6835 0.6324 0.2124 11.00000 0.05 0.20

Q7 1 0.5180 0.9164 0.6246 11.00000 0.05 0.20

Q8 1 0.9640 0.6649 0.5202 11.00000 0.05 0.19

Q9 1 0.9715 0.8945 0.9840 11.00000 0.05 0.19

Q10 1 0.4377 0.9808 0.7101 11.00000 0.05 0.17

Q11 1 0.2179 0.9855 0.4417 11.00000 0.05 0.17

Q12 1 0.0380 0.9168 0.2923 11.00000 0.05 0.17

Q13 1 0.7799 0.8931 0.9743 11.00000 0.05 0.17

Q14 1 1.1034 0.2734 0.8980 11.00000 0.05 0.16

Q15 1 0.7950 0.4989 0.5713 11.00000 0.05 0.16

Q16 1 0.7761 0.9291 1.0312 11.00000 0.05 0.16

Q17 1 0.3563 0.5826 0.0106 11.00000 0.05 0.16

Q18 1 0.6011 0.2667 0.3806 11.00000 0.05 0.16

Q19 1 0.7228 0.9332 0.9820 11.00000 0.05 0.16

Q20 1 1.1979 0.4750 0.7586 11.00000 0.05 0.15

;

_refine_special_details

;

Refinement of F^2^ against ALL reflections. The weighted R-factor wR and

goodness of fit S are based on F^2^, conventional R-factors R are based

on F, with F set to zero for negative F^2^. The threshold expression of

F^2^ > 2sigma(F^2^) is used only for calculating R-factors(gt) etc. and is

not relevant to the choice of reflections for refinement. R-factors based

on F^2^ are statistically about twice as large as those based on F, and R-

factors based on ALL data will be even larger.

;

_refine_ls_structure_factor_coef Fsqd

_refine_ls_matrix_type full

_refine_ls_weighting_scheme calc

_refine_ls_weighting_details

'calc w=1/[\s^2^(Fo^2^)+(0.0568P)^2^+0.5537P] where P=(Fo^2^+2Fc^2^)/3'

_atom_sites_solution_primary direct

_atom_sites_solution_secondary difmap

_atom_sites_solution_hydrogens geom

_refine_ls_hydrogen_treatment mixed

_refine_ls_extinction_method none

_refine_ls_extinction_coef ?

_refine_ls_number_reflns 20881

_refine_ls_number_parameters 1013

_refine_ls_number_restraints 0

_refine_ls_R_factor_all 0.2263

_refine_ls_R_factor_gt 0.0752

_refine_ls_wR_factor_ref 0.2185

_refine_ls_wR_factor_gt 0.1474

_refine_ls_goodness_of_fit_ref 1.013

_refine_ls_restrained_S_all 1.013

_refine_ls_shift/su_max 0.000

_refine_ls_shift/su_mean 0.000

loop_

_atom_site_label

_atom_site_type_symbol

_atom_site_fract_x

_atom_site_fract_y

_atom_site_fract_z

_atom_site_U_iso_or_equiv

_atom_site_adp_type

_atom_site_occupancy

_atom_site_symetry_multiplicity

_atom_site_calc_flag

_atom_site_refinement_flags

_atom_site_disorder_assembly

_atom_site_disorder_group

S1 S 0.92913(6) 0.61670(6) 0.45526(6) 0.0621(3) Uani 1 1 d . . .

S2 S 0.73263(6) 0.68495(6) 0.29021(6) 0.0673(3) Uani 1 1 d . . .

S3 S 0.37269(7) 0.55180(6) -0.11393(6) 0.0717(3) Uani 1 1 d . . .

S4 S 1.14739(7) 0.59195(6) 0.66817(6) 0.0706(3) Uani 1 1 d . . .

N1 N 0.79841(17) 0.70427(17) 0.51136(15) 0.0511(7) Uani 1 1 d . . .

N2 N 0.6993(3) 0.3925(3) 0.6903(3) 0.1221(15) Uani 1 1 d . . .

N3 N 0.9007(3) 0.2537(3) 0.6845(4) 0.1494(19) Uani 1 1 d . . .

N4 N 0.54467(19) 0.70523(19) 0.24703(17) 0.0631(8) Uani 1 1 d . . .

N5 N 0.4340(3) 0.3936(3) 0.4162(4) 0.163(2) Uani 1 1 d . . .

N6 N 0.6524(3) 0.2732(3) 0.4510(3) 0.1095(13) Uani 1 1 d . . .

N7 N 0.27154(19) 0.66558(19) -0.03090(17) 0.0620(8) Uani 1 1 d . . .

N8 N 0.2075(3) 0.3918(3) 0.1913(3) 0.1180(14) Uani 1 1 d . . .

N9 N 0.4092(3) 0.2543(3) 0.1973(3) 0.1216(15) Uani 1 1 d . . .

N10 N 1.02869(19) 0.69277(18) 0.73546(16) 0.0601(7) Uani 1 1 d . . .

N11 N 0.9316(4) 0.3951(4) 0.9226(4) 0.188(3) Uani 1 1 d . . .

N12 N 1.1497(3) 0.2762(3) 0.9607(2) 0.1016(13) Uani 1 1 d . . .

C1 C 0.8757(2) 0.59019(19) 0.51833(17) 0.0463(8) Uani 1 1 d . . .

C2 C 0.8105(2) 0.6322(2) 0.53263(17) 0.0464(8) Uani 1 1 d . . .

C3 C 0.7586(2) 0.5964(2) 0.56825(19) 0.0536(8) Uani 1 1 d . . .

H3 H 0.7130 0.6206 0.5759 0.064 Uiso 1 1 calc R . .

C4 C 0.7724(2) 0.5274(2) 0.59225(19) 0.0550(9) Uani 1 1 d . . .

H4 H 0.7357 0.5055 0.6150 0.066 Uiso 1 1 calc R . .

C5 C 0.8411(2) 0.4893(2) 0.58315(18) 0.0508(8) Uani 1 1 d . . .

C6 C 0.8914(2) 0.5229(2) 0.54533(18) 0.0493(8) Uani 1 1 d . . .

H6 H 0.9373 0.4988 0.5382 0.059 Uiso 1 1 calc R . .

C7 C 0.9333(2) 0.7250(2) 0.4835(2) 0.0578(9) Uani 1 1 d . . .

C8 C 0.9988(3) 0.7769(3) 0.4755(2) 0.0793(11) Uani 1 1 d . . .

H8 H 1.0429 0.7547 0.4620 0.095 Uiso 1 1 calc R . .

C9 C 0.9999(3) 0.8607(3) 0.4871(3) 0.0994(14) Uani 1 1 d . . .

H9 H 1.0434 0.8949 0.4804 0.119 Uiso 1 1 calc R . .

C10 C 0.9354(3) 0.8926(3) 0.5088(3) 0.0884(13) Uani 1 1 d . . .

H10 H 0.9354 0.9492 0.5169 0.106 Uiso 1 1 calc R . .

C11 C 0.8705(3) 0.8424(2) 0.5187(2) 0.0706(10) Uani 1 1 d . . .

H11 H 0.8282 0.8660 0.5347 0.085 Uiso 1 1 calc R . .

C12 C 0.8674(2) 0.7571(2) 0.50506(18) 0.0531(8) Uani 1 1 d . . .

C13 C 0.8646(2) 0.4194(2) 0.6095(2) 0.0615(9) Uani 1 1 d . . .

H13 H 0.9120 0.4026 0.5981 0.074 Uiso 1 1 calc R . .

C14 C 0.8307(3) 0.3731(2) 0.6481(2) 0.0672(10) Uani 1 1 d . . .

C15 C 0.7571(3) 0.3847(3) 0.6708(3) 0.0815(12) Uani 1 1 d . . .

C16 C 0.8694(3) 0.3066(3) 0.6686(3) 0.0952(14) Uani 1 1 d . . .

C17 C 0.7193(2) 0.7360(2) 0.51313(19) 0.0564(9) Uani 1 1 d . . .

H17A H 0.6689 0.6879 0.4900 0.068 Uiso 1 1 calc R . .

H17B H 0.7062 0.7690 0.4751 0.068 Uiso 1 1 calc R . .

C18 C 0.7274(2) 0.7902(2) 0.60136(19) 0.0586(9) Uani 1 1 d . . .

H18A H 0.7347 0.7562 0.6383 0.070 Uiso 1 1 calc R . .

H18B H 0.7802 0.8362 0.6270 0.070 Uiso 1 1 calc R . .

C19 C 0.6469(2) 0.8262(2) 0.59666(19) 0.0602(9) Uani 1 1 d . . .

H19A H 0.6421 0.8630 0.5627 0.072 Uiso 1 1 calc R . .

H19B H 0.5938 0.7802 0.5671 0.072 Uiso 1 1 calc R . .

C20 C 0.6497(2) 0.8751(2) 0.6832(2) 0.0671(10) Uani 1 1 d . . .

H20A H 0.6566 0.8383 0.7173 0.081 Uiso 1 1 calc R . .

H20B H 0.7025 0.9215 0.7118 0.081 Uiso 1 1 calc R . .

C22 C 0.5732(3) 0.9561(3) 0.7703(2) 0.0918(13) Uani 1 1 d . . .

H22A H 0.5832 0.9183 0.8034 0.110 Uiso 1 1 calc R . .

H22B H 0.6244 1.0041 0.7994 0.110 Uiso 1 1 calc R . .

C23 C 0.4943(4) 0.9871(4) 0.7735(3) 0.1226(18) Uani 1 1 d . . .

H23A H 0.4862 1.0273 0.7431 0.147 Uiso 1 1 calc R . .

H23B H 0.4426 0.9397 0.7420 0.147 Uiso 1 1 calc R . .

C24 C 0.4955(4) 1.0275(4) 0.8588(3) 0.148(2) Uani 1 1 d . . .

H24A H 0.5003 0.9877 0.8889 0.222 Uiso 1 1 calc R . .

H24B H 0.4414 1.0459 0.8528 0.222 Uiso 1 1 calc R . .

H24C H 0.5457 1.0753 0.8908 0.222 Uiso 1 1 calc R . .

C25 C 0.6453(2) 0.6190(2) 0.29568(18) 0.0512(8) Uani 1 1 d . . .

C26 C 0.5621(2) 0.6398(2) 0.27865(19) 0.0551(9) Uani 1 1 d . . .

C27 C 0.4997(2) 0.5924(2) 0.2955(2) 0.0606(9) Uani 1 1 d . . .

H27 H 0.4439 0.6045 0.2842 0.073 Uiso 1 1 calc R . .

C28 C 0.5176(2) 0.5291(2) 0.3279(2) 0.0607(9) Uani 1 1 d . . .

H28 H 0.4749 0.5005 0.3404 0.073 Uiso 1 1 calc R . .

C29 C 0.5998(2) 0.5063(2) 0.34262(19) 0.0539(8) Uani 1 1 d . . .

C30 C 0.6617(2) 0.5526(2) 0.32452(18) 0.0532(8) Uani 1 1 d . . .

H30 H 0.7160 0.5382 0.3322 0.064 Uiso 1 1 calc R . .

C31 C 0.6694(3) 0.7129(2) 0.2029(2) 0.0613(9) Uani 1 1 d . . .

C32 C 0.7078(3) 0.7311(2) 0.1497(2) 0.0774(11) Uani 1 1 d . . .

H32 H 0.7654 0.7250 0.1586 0.093 Uiso 1 1 calc R . .

C33 C 0.6616(4) 0.7582(3) 0.0834(3) 0.0887(13) Uani 1 1 d . . .

H33 H 0.6888 0.7731 0.0496 0.106 Uiso 1 1 calc R . .

C34 C 0.5752(3) 0.7629(3) 0.0684(2) 0.0857(13) Uani 1 1 d . . .

H34 H 0.5432 0.7797 0.0230 0.103 Uiso 1 1 calc R . .

C35 C 0.5352(3) 0.7434(2) 0.1192(2) 0.0746(11) Uani 1 1 d . . .

H35 H 0.4760 0.7457 0.1071 0.090 Uiso 1 1 calc R . .

C36 C 0.5827(3) 0.7200(2) 0.1895(2) 0.0606(9) Uani 1 1 d . . .

C37 C 0.6243(2) 0.4399(2) 0.37444(19) 0.0589(9) Uani 1 1 d . . .

H37 H 0.6793 0.4305 0.3761 0.071 Uiso 1 1 calc R . .

C38 C 0.5822(2) 0.3880(2) 0.4027(2) 0.0645(10) Uani 1 1 d . . .

C39 C 0.4996(3) 0.3917(3) 0.4094(3) 0.0923(13) Uani 1 1 d . . .

C40 C 0.6215(3) 0.3240(3) 0.4300(3) 0.0767(11) Uani 1 1 d . . .

C41 C 0.4700(2) 0.7420(2) 0.2525(2) 0.0704(10) Uani 1 1 d . . .

H41A H 0.4156 0.6975 0.2235 0.085 Uiso 1 1 calc R . .

H41B H 0.4635 0.7823 0.2222 0.085 Uiso 1 1 calc R . .

C42 C 0.4806(2) 0.7860(2) 0.3436(2) 0.0685(10) Uani 1 1 d . . .

H42A H 0.5370 0.8279 0.3743 0.082 Uiso 1 1 calc R . .

H42B H 0.4819 0.7450 0.3728 0.082 Uiso 1 1 calc R . .

C43 C 0.4054(2) 0.8288(2) 0.3458(2) 0.0704(10) Uani 1 1 d . . .

H43A H 0.4052 0.8702 0.3171 0.084 Uiso 1 1 calc R . .

H43B H 0.3493 0.7868 0.3134 0.084 Uiso 1 1 calc R . .

C44 C 0.4105(3) 0.8725(2) 0.4347(2) 0.0740(11) Uani 1 1 d . . .

H44A H 0.4102 0.8309 0.4630 0.089 Uiso 1 1 calc R . .

H44B H 0.4671 0.9139 0.4673 0.089 Uiso 1 1 calc R . .

C45 C 0.3371(3) 0.9164(3) 0.4386(2) 0.0818(12) Uani 1 1 d . . .

H45A H 0.3381 0.9587 0.4113 0.098 Uiso 1 1 calc R . .

H45B H 0.2804 0.8752 0.4052 0.098 Uiso 1 1 calc R . .

C46 C 0.3417(3) 0.9585(3) 0.5273(2) 0.0828(12) Uani 1 1 d . . .

H46A H 0.3408 0.9159 0.5544 0.099 Uiso 1 1 calc R . .

H46B H 0.3986 0.9993 0.5606 0.099 Uiso 1 1 calc R . .

C47 C 0.2702(3) 1.0025(3) 0.5333(3) 0.1105(16) Uani 1 1 d . . .

H47A H 0.2134 0.9612 0.5021 0.133 Uiso 1 1 calc R . .

H47B H 0.2695 1.0435 0.5041 0.133 Uiso 1 1 calc R . .

C48 C 0.2772(3) 1.0472(3) 0.6219(3) 0.1122(16) Uani 1 1 d . . .

H48A H 0.2799 1.0080 0.6525 0.168 Uiso 1 1 calc R . .

H48B H 0.2258 1.0700 0.6183 0.168 Uiso 1 1 calc R . .

H48C H 0.3301 1.0922 0.6520 0.168 Uiso 1 1 calc R . .

C49 C 0.3469(2) 0.5498(2) -0.02594(18) 0.0549(9) Uani 1 1 d . . .

C049 C 0.5713(3) 0.9107(3) 0.6835(2) 0.0817(12) Uani 1 1 d . . .

H04A H 0.5181 0.8649 0.6526 0.098 Uiso 1 1 calc R . .

H04B H 0.5660 0.9497 0.6517 0.098 Uiso 1 1 calc R . .

C50 C 0.2934(2) 0.6014(2) 0.0003(2) 0.0580(9) Uani 1 1 d . . .

C51 C 0.2633(2) 0.5837(2) 0.0606(2) 0.0652(10) Uani 1 1 d . . .

H51 H 0.2276 0.6160 0.0791 0.078 Uiso 1 1 calc R . .

C52 C 0.2845(2) 0.5211(2) 0.0930(2) 0.0667(10) Uani 1 1 d . . .

H52 H 0.2613 0.5106 0.1314 0.080 Uiso 1 1 calc R . .

C53 C 0.3404(2) 0.4722(2) 0.0698(2) 0.0597(9) Uani 1 1 d . . .

C54 C 0.3713(2) 0.4899(2) 0.0103(2) 0.0605(9) Uani 1 1 d . . .

H54 H 0.4100 0.4598 -0.0053 0.073 Uiso 1 1 calc R . .

C55 C 0.3665(2) 0.6546(2) -0.1121(2) 0.0631(10) Uani 1 1 d . . .

C56 C 0.4093(3) 0.6893(3) -0.1541(2) 0.0776(11) Uani 1 1 d . . .

H56 H 0.4422 0.6586 -0.1791 0.093 Uiso 1 1 calc R . .

C57 C 0.4048(3) 0.7674(3) -0.1601(3) 0.0966(14) Uani 1 1 d . . .

H57 H 0.4337 0.7898 -0.1890 0.116 Uiso 1 1 calc R . .

C58 C 0.3564(3) 0.8121(3) -0.1223(3) 0.0996(14) Uani 1 1 d . . .

H58 H 0.3520 0.8651 -0.1263 0.120 Uiso 1 1 calc R . .

C59 C 0.3148(3) 0.7798(3) -0.0788(2) 0.0800(11) Uani 1 1 d . . .

H59 H 0.2842 0.8123 -0.0522 0.096 Uiso 1 1 calc R . .

C60 C 0.3170(2) 0.6998(2) -0.0734(2) 0.0621(10) Uani 1 1 d . . .

C61 C 0.3674(2) 0.4061(2) 0.1023(2) 0.0671(10) Uani 1 1 d . . .

H61 H 0.4138 0.3872 0.0902 0.080 Uiso 1 1 calc R . .

C62 C 0.3364(3) 0.3670(2) 0.1473(2) 0.0673(10) Uani 1 1 d . . .

C63 C 0.2642(3) 0.3821(3) 0.1713(3) 0.0802(12) Uani 1 1 d . . .

C64 C 0.3769(3) 0.3040(3) 0.1741(3) 0.0854(13) Uani 1 1 d . . .

C65 C 0.2033(2) 0.7059(2) -0.0125(2) 0.0682(10) Uani 1 1 d . . .

H65A H 0.1573 0.6626 -0.0152 0.082 Uiso 1 1 calc R . .

H65B H 0.1761 0.7282 -0.0579 0.082 Uiso 1 1 calc R . .

C66 C 0.2345(2) 0.7769(2) 0.0734(2) 0.0704(10) Uani 1 1 d . . .

H66A H 0.2805 0.8211 0.0775 0.084 Uiso 1 1 calc R . .

H66B H 0.2598 0.7554 0.1199 0.084 Uiso 1 1 calc R . .

C67 C 0.1564(3) 0.8133(2) 0.0821(2) 0.0801(11) Uani 1 1 d . . .

H67A H 0.1393 0.8435 0.0418 0.096 Uiso 1 1 calc R . .

H67B H 0.1063 0.7666 0.0649 0.096 Uiso 1 1 calc R . .

C68 C 0.1719(3) 0.8710(3) 0.1684(3) 0.0988(14) Uani 1 1 d . . .

H68A H 0.2170 0.9214 0.1833 0.119 Uiso 1 1 calc R . .

H68B H 0.1950 0.8436 0.2103 0.119 Uiso 1 1 calc R . .

C69 C 0.0868(3) 0.8974(3) 0.1746(3) 0.1200(19) Uani 1 1 d . . .

H69A H 0.0644 0.9252 0.1329 0.144 Uiso 1 1 calc R . .

H69B H 0.0417 0.8466 0.1583 0.144 Uiso 1 1 calc R . .

C70 C 0.0975(4) 0.9514(4) 0.2559(3) 0.145(2) Uani 1 1 d . . .

H70A H 0.1416 1.0030 0.2720 0.174 Uiso 1 1 calc R . .

H70B H 0.1205 0.9243 0.2980 0.174 Uiso 1 1 calc R . .

C71 C 0.0123(4) 0.9744(4) 0.2593(3) 0.133(2) Uani 1 1 d . . .

H71A H 0.0286 1.0216 0.3120 0.159 Uiso 1 1 calc R . .

H71B H -0.0141 0.9943 0.2118 0.159 Uiso 1 1 calc R . .

C72 C -0.0576(4) 0.9080(4) 0.2557(4) 0.159(2) Uani 1 1 d . . .

H72A H -0.0850 0.8669 0.1987 0.239 Uiso 1 1 calc R . .

H72B H -0.1019 0.9333 0.2699 0.239 Uiso 1 1 calc R . .

H72C H -0.0310 0.8809 0.2962 0.239 Uiso 1 1 calc R . .

C73 C 1.1041(2) 0.5774(2) 0.74256(19) 0.0544(9) Uani 1 1 d . . .

C74 C 1.0431(2) 0.6229(2) 0.76026(19) 0.0543(9) Uani 1 1 d . . .

C75 C 0.9971(2) 0.5945(2) 0.8052(2) 0.0644(10) Uani 1 1 d . . .

H75 H 0.9535 0.6209 0.8150 0.077 Uiso 1 1 calc R . .

C76 C 1.0145(2) 0.5294(2) 0.8349(2) 0.0648(10) Uani 1 1 d . . .

H76 H 0.9821 0.5122 0.8638 0.078 Uiso 1 1 calc R . .

C77 C 1.0805(2) 0.4878(2) 0.8229(2) 0.0581(9) Uani 1 1 d . . .

C78 C 1.1238(2) 0.5141(2) 0.77549(19) 0.0594(9) Uani 1 1 d . . .

H78 H 1.1675 0.4878 0.7659 0.071 Uiso 1 1 calc R . .

C79 C 1.1477(3) 0.6982(2) 0.6829(2) 0.0676(10) Uani 1 1 d . . .

C80 C 1.2057(3) 0.7428(3) 0.6604(3) 0.0918(13) Uani 1 1 d . . .

H80 H 1.2451 0.7164 0.6419 0.110 Uiso 1 1 calc R . .

C81 C 1.2068(4) 0.8250(4) 0.6646(3) 0.1158(17) Uani 1 1 d . . .

H81 H 1.2449 0.8537 0.6473 0.139 Uiso 1 1 calc R . .

C82 C 1.1503(4) 0.8637(3) 0.6948(3) 0.1129(17) Uani 1 1 d . . .

H82 H 1.1508 0.9198 0.6991 0.135 Uiso 1 1 calc R . .

C83 C 1.0926(3) 0.8209(3) 0.7191(3) 0.0867(12) Uani 1 1 d . . .

H83 H 1.0553 0.8486 0.7400 0.104 Uiso 1 1 calc R . .

C84 C 1.0896(3) 0.7367(2) 0.7126(2) 0.0662(10) Uani 1 1 d . . .

C85 C 1.1083(2) 0.4241(2) 0.8584(2) 0.0640(10) Uani 1 1 d . . .

H85 H 1.1586 0.4090 0.8520 0.077 Uiso 1 1 calc R . .

C86 C 1.0742(3) 0.3822(2) 0.8997(2) 0.0694(10) Uani 1 1 d . . .

C87 C 0.9957(4) 0.3909(3) 0.9129(4) 0.1094(16) Uani 1 1 d . . .

C88 C 1.1165(3) 0.3230(3) 0.9332(3) 0.0761(12) Uani 1 1 d . . .

C89 C 0.9560(2) 0.7299(2) 0.7467(2) 0.0680(10) Uani 1 1 d . . .

H89A H 0.9056 0.6843 0.7320 0.082 Uiso 1 1 calc R . .

H89B H 0.9376 0.7595 0.7060 0.082 Uiso 1 1 calc R . .

C90 C 0.9770(2) 0.7906(2) 0.8357(2) 0.0713(11) Uani 1 1 d . . .

H90A H 1.0277 0.8363 0.8516 0.086 Uiso 1 1 calc R . .

H90B H 0.9931 0.7612 0.8769 0.086 Uiso 1 1 calc R . .

C91 C 0.8984(2) 0.8271(2) 0.8400(2) 0.0711(10) Uani 1 1 d . . .

H91A H 0.8883 0.8628 0.8047 0.085 Uiso 1 1 calc R . .

H91B H 0.8458 0.7810 0.8149 0.085 Uiso 1 1 calc R . .

C92 C 0.9078(3) 0.8773(2) 0.9273(2) 0.0801(12) Uani 1 1 d . . .

H92A H 0.9600 0.9239 0.9522 0.096 Uiso 1 1 calc R . .

H92B H 0.9187 0.8419 0.9629 0.096 Uiso 1 1 calc R . .

C93 C 0.8299(3) 0.9126(3) 0.9319(3) 0.0967(14) Uani 1 1 d . . .

H93A H 0.8187 0.9476 0.8959 0.116 Uiso 1 1 calc R . .

H93B H 0.7779 0.8659 0.9073 0.116 Uiso 1 1 calc R . .

C94 C 0.8382(4) 0.9634(4) 1.0184(3) 0.139(2) Uani 1 1 d . . .

H94A H 0.8903 1.0101 1.0431 0.167 Uiso 1 1 calc R . .

H94B H 0.8492 0.9284 1.0544 0.167 Uiso 1 1 calc R . .

C95 C 0.7596(5) 0.9994(5) 1.0233(4) 0.172(3) Uani 1 1 d . . .

H95A H 0.7831 1.0544 1.0684 0.206 Uiso 1 1 calc R . .

H95B H 0.7302 1.0089 0.9695 0.206 Uiso 1 1 calc R . .

C96 C 0.7013(6) 0.9586(6) 1.0368(8) 0.288(7) Uani 1 1 d . . .

H96A H 0.6882 0.8998 1.0034 0.433 Uiso 1 1 calc R . .

H96B H 0.6482 0.9792 1.0207 0.433 Uiso 1 1 calc R . .

H96C H 0.7227 0.9653 1.0969 0.433 Uiso 1 1 calc R . .

loop_

_atom_site_aniso_label

_atom_site_aniso_U_11

_atom_site_aniso_U_22

_atom_site_aniso_U_33

_atom_site_aniso_U_23

_atom_site_aniso_U_13

_atom_site_aniso_U_12

S1 0.0700(6) 0.0619(6) 0.0824(6) 0.0329(5) 0.0516(5) 0.0299(5)

S2 0.0571(6) 0.0751(7) 0.0750(6) 0.0295(5) 0.0297(5) 0.0156(5)

S3 0.0835(7) 0.0750(7) 0.0701(6) 0.0194(5) 0.0486(5) 0.0244(5)

S4 0.0805(7) 0.0728(7) 0.0820(6) 0.0313(5) 0.0524(5) 0.0288(5)

N1 0.0484(17) 0.0517(17) 0.0603(15) 0.0182(13) 0.0274(13) 0.0225(14)

N2 0.134(4) 0.114(3) 0.180(4) 0.062(3) 0.122(3) 0.038(3)

N3 0.166(5) 0.142(4) 0.232(5) 0.133(4) 0.117(4) 0.081(4)

N4 0.063(2) 0.070(2) 0.0647(17) 0.0253(15) 0.0297(15) 0.0277(16)

N5 0.129(4) 0.164(5) 0.310(7) 0.137(5) 0.162(5) 0.076(4)

N6 0.107(3) 0.105(3) 0.154(3) 0.078(3) 0.066(3) 0.038(3)

N7 0.0620(19) 0.064(2) 0.0653(17) 0.0166(15) 0.0350(15) 0.0177(16)

N8 0.129(4) 0.124(4) 0.160(4) 0.064(3) 0.108(3) 0.048(3)

N9 0.117(4) 0.129(4) 0.181(4) 0.097(3) 0.089(3) 0.052(3)

N10 0.0586(19) 0.0576(19) 0.0637(16) 0.0129(14) 0.0275(14) 0.0216(16)

N11 0.186(5) 0.210(6) 0.343(8) 0.191(6) 0.212(6) 0.115(5)

N12 0.102(3) 0.103(3) 0.115(3) 0.057(2) 0.047(2) 0.024(2)

C1 0.0442(19) 0.050(2) 0.0506(16) 0.0159(14) 0.0255(14) 0.0162(16)

C2 0.0427(19) 0.047(2) 0.0479(16) 0.0091(14) 0.0216(15) 0.0119(16)

C3 0.047(2) 0.054(2) 0.0651(19) 0.0133(16) 0.0321(16) 0.0171(17)

C4 0.055(2) 0.055(2) 0.0630(19) 0.0152(16) 0.0364(17) 0.0097(18)

C5 0.046(2) 0.056(2) 0.0581(18) 0.0198(16) 0.0284(16) 0.0141(17)

C6 0.0435(19) 0.055(2) 0.0616(18) 0.0211(16) 0.0308(16) 0.0202(16)

C7 0.056(2) 0.057(2) 0.071(2) 0.0265(17) 0.0318(18) 0.0185(18)

C8 0.080(3) 0.072(3) 0.116(3) 0.049(2) 0.059(2) 0.026(2)

C9 0.103(4) 0.072(3) 0.154(4) 0.057(3) 0.073(3) 0.021(3)

C10 0.107(4) 0.061(3) 0.117(3) 0.044(2) 0.056(3) 0.026(3)

C11 0.082(3) 0.059(3) 0.083(2) 0.0264(19) 0.042(2) 0.028(2)

C12 0.056(2) 0.051(2) 0.0542(18) 0.0183(15) 0.0236(16) 0.0154(18)

C13 0.056(2) 0.070(3) 0.074(2) 0.0311(19) 0.0367(18) 0.0206(19)

C14 0.070(3) 0.066(3) 0.081(2) 0.031(2) 0.042(2) 0.020(2)

C15 0.098(4) 0.069(3) 0.110(3) 0.041(2) 0.068(3) 0.024(3)

C16 0.100(4) 0.098(4) 0.135(4) 0.070(3) 0.073(3) 0.036(3)

C17 0.054(2) 0.059(2) 0.0609(19) 0.0160(16) 0.0267(16) 0.0285(17)

C18 0.058(2) 0.053(2) 0.0627(19) 0.0095(15) 0.0263(17) 0.0231(17)

C19 0.066(2) 0.057(2) 0.066(2) 0.0203(16) 0.0320(18) 0.0298(18)

C20 0.071(3) 0.066(2) 0.074(2) 0.0192(18) 0.0412(19) 0.029(2)

C22 0.110(4) 0.091(3) 0.091(3) 0.020(2) 0.060(3) 0.051(3)

C23 0.135(5) 0.143(5) 0.115(4) 0.033(3) 0.073(3) 0.079(4)

C24 0.177(6) 0.147(5) 0.158(5) 0.027(4) 0.119(4) 0.069(4)

C25 0.045(2) 0.061(2) 0.0473(17) 0.0122(15) 0.0234(15) 0.0135(17)

C26 0.051(2) 0.058(2) 0.0522(18) 0.0093(16) 0.0225(16) 0.0172(18)

C27 0.043(2) 0.070(3) 0.069(2) 0.0174(18) 0.0255(17) 0.0188(19)

C28 0.051(2) 0.061(2) 0.070(2) 0.0161(18) 0.0290(17) 0.0101(18)

C29 0.050(2) 0.053(2) 0.0581(19) 0.0107(16) 0.0266(16) 0.0136(18)

C30 0.047(2) 0.061(2) 0.0529(18) 0.0149(16) 0.0226(15) 0.0183(18)

C31 0.070(3) 0.052(2) 0.059(2) 0.0109(16) 0.0294(19) 0.0118(19)

C32 0.091(3) 0.071(3) 0.086(3) 0.029(2) 0.051(2) 0.024(2)

C33 0.122(4) 0.079(3) 0.080(3) 0.032(2) 0.054(3) 0.023(3)

C34 0.106(4) 0.083(3) 0.062(2) 0.025(2) 0.028(3) 0.018(3)

C35 0.078(3) 0.078(3) 0.063(2) 0.024(2) 0.022(2) 0.021(2)

C36 0.067(3) 0.056(2) 0.055(2) 0.0142(16) 0.0224(18) 0.0143(19)

C37 0.050(2) 0.064(2) 0.068(2) 0.0196(18) 0.0306(17) 0.0149(18)

C38 0.060(2) 0.065(3) 0.074(2) 0.0217(19) 0.0341(19) 0.017(2)

C39 0.083(3) 0.091(3) 0.146(4) 0.061(3) 0.077(3) 0.035(3)

C40 0.071(3) 0.076(3) 0.099(3) 0.038(2) 0.047(2) 0.016(2)

C41 0.067(3) 0.076(3) 0.078(2) 0.0281(19) 0.032(2) 0.038(2)

C42 0.064(2) 0.064(2) 0.077(2) 0.0165(18) 0.0302(19) 0.0243(19)

C43 0.070(3) 0.063(2) 0.088(2) 0.0279(19) 0.037(2) 0.030(2)

C44 0.074(3) 0.068(3) 0.091(3) 0.026(2) 0.043(2) 0.029(2)

C45 0.077(3) 0.085(3) 0.102(3) 0.037(2) 0.047(2) 0.041(2)

C46 0.089(3) 0.077(3) 0.103(3) 0.034(2) 0.055(2) 0.036(2)

C47 0.111(4) 0.130(4) 0.114(3) 0.037(3) 0.061(3) 0.073(3)

C48 0.132(4) 0.112(4) 0.135(4) 0.043(3) 0.089(3) 0.067(3)

C49 0.053(2) 0.057(2) 0.0540(18) 0.0098(16) 0.0295(16) 0.0107(18)

C049 0.083(3) 0.089(3) 0.083(2) 0.021(2) 0.045(2) 0.039(2)

C50 0.053(2) 0.058(2) 0.0571(19) 0.0109(17) 0.0244(17) 0.0059(18)

C51 0.067(2) 0.065(3) 0.070(2) 0.0126(18) 0.0412(19) 0.017(2)

C52 0.071(3) 0.067(3) 0.068(2) 0.0151(19) 0.0428(19) 0.011(2)

C53 0.059(2) 0.059(2) 0.063(2) 0.0144(17) 0.0319(18) 0.0114(19)

C54 0.055(2) 0.061(2) 0.068(2) 0.0133(18) 0.0336(18) 0.0148(18)

C55 0.058(2) 0.074(3) 0.0570(19) 0.0212(18) 0.0246(18) 0.014(2)

C56 0.078(3) 0.092(3) 0.075(2) 0.035(2) 0.040(2) 0.021(2)

C57 0.111(4) 0.107(4) 0.100(3) 0.055(3) 0.058(3) 0.028(3)

C58 0.124(4) 0.094(4) 0.113(3) 0.056(3) 0.064(3) 0.038(3)

C59 0.097(3) 0.076(3) 0.084(3) 0.032(2) 0.048(2) 0.032(2)

C60 0.061(2) 0.066(3) 0.0557(19) 0.0165(17) 0.0228(18) 0.016(2)

C61 0.064(2) 0.067(3) 0.074(2) 0.0187(19) 0.036(2) 0.015(2)

C62 0.069(3) 0.066(3) 0.070(2) 0.0220(19) 0.033(2) 0.011(2)

C63 0.086(3) 0.081(3) 0.093(3) 0.033(2) 0.057(3) 0.019(3)

C64 0.079(3) 0.091(4) 0.109(3) 0.047(3) 0.054(3) 0.020(3)

C65 0.061(2) 0.072(3) 0.071(2) 0.0165(18) 0.0299(19) 0.021(2)

C66 0.073(3) 0.067(2) 0.074(2) 0.0160(18) 0.037(2) 0.022(2)

C67 0.093(3) 0.076(3) 0.087(3) 0.028(2) 0.050(2) 0.035(2)

C68 0.123(4) 0.095(3) 0.097(3) 0.032(3) 0.057(3) 0.053(3)

C69 0.140(5) 0.120(4) 0.091(3) 0.016(3) 0.035(3) 0.080(4)

C70 0.154(6) 0.143(5) 0.125(4) 0.028(4) 0.046(4) 0.062(4)

C71 0.120(5) 0.162(6) 0.110(4) 0.017(3) 0.053(3) 0.061(4)

C72 0.150(6) 0.166(6) 0.173(6) 0.027(5) 0.102(5) 0.037(5)

C73 0.047(2) 0.059(2) 0.0568(18) 0.0140(16) 0.0247(16) 0.0128(17)

C74 0.049(2) 0.055(2) 0.0552(18) 0.0105(16) 0.0228(16) 0.0111(17)

C75 0.055(2) 0.069(3) 0.073(2) 0.0147(19) 0.0359(19) 0.0208(19)

C76 0.062(2) 0.065(2) 0.072(2) 0.0178(19) 0.0384(19) 0.009(2)

C77 0.051(2) 0.060(2) 0.067(2) 0.0171(17) 0.0308(18) 0.0129(18)

C78 0.052(2) 0.065(2) 0.064(2) 0.0182(18) 0.0281(17) 0.0195(18)

C79 0.062(2) 0.074(3) 0.079(2) 0.036(2) 0.033(2) 0.018(2)

C80 0.092(3) 0.094(4) 0.125(3) 0.061(3) 0.062(3) 0.035(3)

C81 0.116(4) 0.108(4) 0.166(5) 0.086(4) 0.074(4) 0.034(3)

C82 0.139(5) 0.086(4) 0.142(4) 0.060(3) 0.068(4) 0.041(4)

C83 0.095(3) 0.078(3) 0.097(3) 0.037(2) 0.040(3) 0.031(3)

C84 0.071(3) 0.063(3) 0.067(2) 0.0247(18) 0.027(2) 0.020(2)

C85 0.060(2) 0.066(2) 0.068(2) 0.0203(18) 0.0305(18) 0.0151(19)

C86 0.070(3) 0.068(3) 0.077(2) 0.025(2) 0.038(2) 0.013(2)

C87 0.112(4) 0.112(4) 0.178(5) 0.087(4) 0.106(4) 0.051(3)

C88 0.076(3) 0.072(3) 0.084(3) 0.028(2) 0.039(2) 0.005(2)

C89 0.063(2) 0.064(2) 0.069(2) 0.0104(18) 0.0243(18) 0.0250(19)

C90 0.074(3) 0.062(2) 0.074(2) 0.0120(18) 0.030(2) 0.027(2)

C91 0.077(3) 0.063(2) 0.076(2) 0.0181(18) 0.033(2) 0.029(2)

C92 0.089(3) 0.078(3) 0.082(3) 0.022(2) 0.042(2) 0.038(2)

C93 0.104(4) 0.106(4) 0.094(3) 0.028(2) 0.053(3) 0.050(3)

C94 0.138(5) 0.182(6) 0.117(4) 0.033(4) 0.072(4) 0.086(4)

C95 0.202(8) 0.240(9) 0.133(5) 0.068(5) 0.108(5) 0.130(7)

C96 0.288(12) 0.256(11) 0.59(2) 0.289(13) 0.339(14) 0.159(9)

_geom_special_details

;

All esds (except the esd in the dihedral angle between two l.s. planes)

are estimated using the full covariance matrix. The cell esds are taken

into account individually in the estimation of esds in distances, angles

and torsion angles; correlations between esds in cell parameters are only

used when they are defined by crystal symmetry. An approximate (isotropic)

treatment of cell esds is used for estimating esds involving l.s. planes.

;

loop_

_geom_bond_atom_site_label_1

_geom_bond_atom_site_label_2

_geom_bond_distance

_geom_bond_site_symmetry_2

_geom_bond_publ_flag

S1 C7 1.750(4) . ?

S1 C1 1.757(3) . ?

S2 C31 1.755(4) . ?

S2 C25 1.763(3) . ?

S3 C55 1.749(4) . ?

S3 C49 1.754(3) . ?

S4 C79 1.745(4) . ?

S4 C73 1.757(3) . ?

N1 C2 1.389(4) . ?

N1 C12 1.418(4) . ?

N1 C17 1.474(4) . ?

N2 C15 1.131(5) . ?

N3 C16 1.137(5) . ?

N4 C26 1.388(4) . ?

N4 C36 1.426(4) . ?

N4 C41 1.464(4) . ?

N5 C39 1.131(5) . ?

N6 C40 1.134(5) . ?

N7 C50 1.379(4) . ?

N7 C60 1.412(4) . ?

N7 C65 1.466(4) . ?

N8 C63 1.123(5) . ?

N9 C64 1.142(5) . ?

N10 C74 1.389(4) . ?

N10 C84 1.413(5) . ?

N10 C89 1.465(4) . ?

N11 C87 1.135(6) . ?

N12 C88 1.136(5) . ?

C1 C6 1.370(4) . ?

C1 C2 1.412(4) . ?

C2 C3 1.399(4) . ?

C3 C4 1.366(4) . ?

C3 H3 0.9300 . ?

C4 C5 1.402(4) . ?

C4 H4 0.9300 . ?

C5 C6 1.394(4) . ?

C5 C13 1.432(4) . ?

C6 H6 0.9300 . ?

C7 C8 1.381(5) . ?

C7 C12 1.391(4) . ?

C8 C9 1.374(5) . ?

C8 H8 0.9300 . ?

C9 C10 1.368(6) . ?

C9 H9 0.9300 . ?

C10 C11 1.378(5) . ?

C10 H10 0.9300 . ?

C11 C12 1.390(4) . ?

C11 H11 0.9300 . ?

C13 C14 1.360(5) . ?

C13 H13 0.9300 . ?

C14 C15 1.423(6) . ?

C14 C16 1.428(6) . ?

C17 C18 1.523(4) . ?

C17 H17A 0.9700 . ?

C17 H17B 0.9700 . ?

C18 C19 1.510(4) . ?

C18 H18A 0.9700 . ?

C18 H18B 0.9700 . ?

C19 C20 1.504(4) . ?

C19 H19A 0.9700 . ?

C19 H19B 0.9700 . ?

C20 C049 1.490(5) . ?

C20 H20A 0.9700 . ?

C20 H20B 0.9700 . ?

C22 C23 1.471(6) . ?

C22 C049 1.499(5) . ?

C22 H22A 0.9700 . ?

C22 H22B 0.9700 . ?

C23 C24 1.464(6) . ?

C23 H23A 0.9700 . ?

C23 H23B 0.9700 . ?

C24 H24A 0.9600 . ?

C24 H24B 0.9600 . ?

C24 H24C 0.9600 . ?

C25 C30 1.373(4) . ?

C25 C26 1.407(4) . ?

C26 C27 1.394(5) . ?

C27 C28 1.364(5) . ?

C27 H27 0.9300 . ?

C28 C29 1.408(5) . ?

C28 H28 0.9300 . ?

C29 C30 1.392(4) . ?

C29 C37 1.429(5) . ?

C30 H30 0.9300 . ?

C31 C32 1.380(5) . ?

C31 C36 1.390(5) . ?

C32 C33 1.384(5) . ?

C32 H32 0.9300 . ?

C33 C34 1.371(6) . ?

C33 H33 0.9300 . ?

C34 C35 1.369(5) . ?

C34 H34 0.9300 . ?

C35 C36 1.403(5) . ?

C35 H35 0.9300 . ?

C37 C38 1.357(5) . ?

C37 H37 0.9300 . ?

C38 C39 1.416(6) . ?

C38 C40 1.435(6) . ?

C41 C42 1.510(4) . ?

C41 H41A 0.9700 . ?

C41 H41B 0.9700 . ?

C42 C43 1.516(5) . ?

C42 H42A 0.9700 . ?

C42 H42B 0.9700 . ?

C43 C44 1.505(5) . ?

C43 H43A 0.9700 . ?

C43 H43B 0.9700 . ?

C44 C45 1.504(5) . ?

C44 H44A 0.9700 . ?

C44 H44B 0.9700 . ?

C45 C46 1.501(5) . ?

C45 H45A 0.9700 . ?

C45 H45B 0.9700 . ?

C46 C47 1.485(5) . ?

C46 H46A 0.9700 . ?

C46 H46B 0.9700 . ?

C47 C48 1.494(5) . ?

C47 H47A 0.9700 . ?

C47 H47B 0.9700 . ?

C48 H48A 0.9600 . ?

C48 H48B 0.9600 . ?

C48 H48C 0.9600 . ?

C49 C54 1.369(4) . ?

C49 C50 1.414(5) . ?

C049 H04A 0.9700 . ?

C049 H04B 0.9700 . ?

C50 C51 1.406(5) . ?

C51 C52 1.362(5) . ?

C51 H51 0.9300 . ?

C52 C53 1.400(5) . ?

C52 H52 0.9300 . ?

C53 C54 1.402(4) . ?

C53 C61 1.440(5) . ?

C54 H54 0.9300 . ?

C55 C56 1.379(5) . ?

C55 C60 1.403(5) . ?

C56 C57 1.364(5) . ?

C56 H56 0.9300 . ?

C57 C58 1.375(6) . ?

C57 H57 0.9300 . ?

C58 C59 1.367(5) . ?

C58 H58 0.9300 . ?

C59 C60 1.388(5) . ?

C59 H59 0.9300 . ?

C61 C62 1.352(5) . ?

C61 H61 0.9300 . ?

C62 C63 1.423(6) . ?

C62 C64 1.425(6) . ?

C65 C66 1.520(4) . ?

C65 H65A 0.9700 . ?

C65 H65B 0.9700 . ?

C66 C67 1.527(5) . ?

C66 H66A 0.9700 . ?

C66 H66B 0.9700 . ?

C67 C68 1.478(5) . ?

C67 H67A 0.9700 . ?

C67 H67B 0.9700 . ?

C68 C69 1.552(6) . ?

C68 H68A 0.9700 . ?

C68 H68B 0.9700 . ?

C69 C70 1.408(6) . ?

C69 H69A 0.9700 . ?

C69 H69B 0.9700 . ?

C70 C71 1.517(7) . ?

C70 H70A 0.9700 . ?

C70 H70B 0.9700 . ?

C71 C72 1.484(7) . ?

C71 H71A 0.9700 . ?

C71 H71B 0.9700 . ?

C72 H72A 0.9600 . ?

C72 H72B 0.9600 . ?

C72 H72C 0.9600 . ?

C73 C78 1.374(4) . ?

C73 C74 1.404(4) . ?

C74 C75 1.407(5) . ?

C75 C76 1.364(5) . ?

C75 H75 0.9300 . ?

C76 C77 1.408(5) . ?

C76 H76 0.9300 . ?

C77 C78 1.400(4) . ?

C77 C85 1.433(5) . ?

C78 H78 0.9300 . ?

C79 C80 1.379(5) . ?

C79 C84 1.390(5) . ?

C80 C81 1.371(6) . ?

C80 H80 0.9300 . ?

C81 C82 1.372(7) . ?

C81 H81 0.9300 . ?

C82 C83 1.382(6) . ?

C82 H82 0.9300 . ?

C83 C84 1.392(5) . ?

C83 H83 0.9300 . ?

C85 C86 1.351(5) . ?

C85 H85 0.9300 . ?

C86 C87 1.410(6) . ?

C86 C88 1.431(6) . ?

C89 C90 1.513(4) . ?

C89 H89A 0.9700 . ?

C89 H89B 0.9700 . ?

C90 C91 1.516(5) . ?

C90 H90A 0.9700 . ?

C90 H90B 0.9700 . ?

C91 C92 1.483(5) . ?

C91 H91A 0.9700 . ?

C91 H91B 0.9700 . ?

C92 C93 1.496(5) . ?

C92 H92A 0.9700 . ?

C92 H92B 0.9700 . ?

C93 C94 1.479(6) . ?

C93 H93A 0.9700 . ?

C93 H93B 0.9700 . ?

C94 C95 1.513(7) . ?

C94 H94A 0.9700 . ?

C94 H94B 0.9700 . ?

C95 C96 1.246(9) . ?

C95 H95A 0.9700 . ?

C95 H95B 0.9700 . ?

C96 H96A 0.9600 . ?

C96 H96B 0.9600 . ?

C96 H96C 0.9600 . ?

loop_

_geom_angle_atom_site_label_1

_geom_angle_atom_site_label_2

_geom_angle_atom_site_label_3

_geom_angle

_geom_angle_site_symmetry_1

_geom_angle_site_symmetry_3

_geom_angle_publ_flag

C7 S1 C1 99.59(16) . . ?

C31 S2 C25 98.62(17) . . ?

C55 S3 C49 100.60(17) . . ?

C79 S4 C73 99.91(18) . . ?

C2 N1 C12 121.8(3) . . ?

C2 N1 C17 118.6(3) . . ?

C12 N1 C17 118.3(3) . . ?

C26 N4 C36 119.0(3) . . ?

C26 N4 C41 119.7(3) . . ?

C36 N4 C41 119.0(3) . . ?

C50 N7 C60 123.3(3) . . ?

C50 N7 C65 118.8(3) . . ?

C60 N7 C65 117.7(3) . . ?

C74 N10 C84 122.1(3) . . ?

C74 N10 C89 118.5(3) . . ?

C84 N10 C89 118.7(3) . . ?

C6 C1 C2 120.9(3) . . ?

C6 C1 S1 118.9(2) . . ?

C2 C1 S1 119.8(2) . . ?

N1 C2 C3 122.4(3) . . ?

N1 C2 C1 121.5(3) . . ?

C3 C2 C1 116.2(3) . . ?

C4 C3 C2 122.6(3) . . ?

C4 C3 H3 118.7 . . ?

C2 C3 H3 118.7 . . ?

C3 C4 C5 121.0(3) . . ?

C3 C4 H4 119.5 . . ?

C5 C4 H4 119.5 . . ?

C6 C5 C4 116.8(3) . . ?

C6 C5 C13 117.0(3) . . ?

C4 C5 C13 126.1(3) . . ?

C1 C6 C5 122.3(3) . . ?

C1 C6 H6 118.9 . . ?

C5 C6 H6 118.9 . . ?

C8 C7 C12 120.4(3) . . ?

C8 C7 S1 118.2(3) . . ?

C12 C7 S1 121.1(3) . . ?

C9 C8 C7 121.3(4) . . ?

C9 C8 H8 119.3 . . ?

C7 C8 H8 119.3 . . ?

C10 C9 C8 118.4(4) . . ?

C10 C9 H9 120.8 . . ?

C8 C9 H9 120.8 . . ?

C9 C10 C11 121.3(4) . . ?

C9 C10 H10 119.4 . . ?

C11 C10 H10 119.4 . . ?

C10 C11 C12 120.8(4) . . ?

C10 C11 H11 119.6 . . ?

C12 C11 H11 119.6 . . ?

C11 C12 C7 117.7(3) . . ?

C11 C12 N1 121.2(3) . . ?

C7 C12 N1 121.1(3) . . ?

C14 C13 C5 132.2(3) . . ?

C14 C13 H13 113.9 . . ?

C5 C13 H13 113.9 . . ?

C13 C14 C15 125.2(4) . . ?

C13 C14 C16 119.9(4) . . ?

C15 C14 C16 114.9(3) . . ?

N2 C15 C14 178.0(5) . . ?

N3 C16 C14 179.4(5) . . ?

N1 C17 C18 115.6(2) . . ?

N1 C17 H17A 108.4 . . ?

C18 C17 H17A 108.4 . . ?

N1 C17 H17B 108.4 . . ?

C18 C17 H17B 108.4 . . ?

H17A C17 H17B 107.4 . . ?

C19 C18 C17 112.3(3) . . ?

C19 C18 H18A 109.1 . . ?

C17 C18 H18A 109.1 . . ?

C19 C18 H18B 109.1 . . ?

C17 C18 H18B 109.1 . . ?

H18A C18 H18B 107.9 . . ?

C20 C19 C18 113.8(3) . . ?

C20 C19 H19A 108.8 . . ?

C18 C19 H19A 108.8 . . ?

C20 C19 H19B 108.8 . . ?

C18 C19 H19B 108.8 . . ?

H19A C19 H19B 107.7 . . ?

C049 C20 C19 116.7(3) . . ?

C049 C20 H20A 108.1 . . ?

C19 C20 H20A 108.1 . . ?

C049 C20 H20B 108.1 . . ?

C19 C20 H20B 108.1 . . ?

H20A C20 H20B 107.3 . . ?

C23 C22 C049 117.7(4) . . ?

C23 C22 H22A 107.9 . . ?

C049 C22 H22A 107.9 . . ?

C23 C22 H22B 107.9 . . ?

C049 C22 H22B 107.9 . . ?

H22A C22 H22B 107.2 . . ?

C24 C23 C22 117.0(4) . . ?

C24 C23 H23A 108.0 . . ?

C22 C23 H23A 108.0 . . ?

C24 C23 H23B 108.0 . . ?

C22 C23 H23B 108.0 . . ?

H23A C23 H23B 107.3 . . ?

C23 C24 H24A 109.5 . . ?

C23 C24 H24B 109.5 . . ?

H24A C24 H24B 109.5 . . ?

C23 C24 H24C 109.5 . . ?

H24A C24 H24C 109.5 . . ?

H24B C24 H24C 109.5 . . ?

C30 C25 C26 120.6(3) . . ?

C30 C25 S2 119.6(3) . . ?

C26 C25 S2 119.5(3) . . ?

N4 C26 C27 122.4(3) . . ?

N4 C26 C25 120.5(3) . . ?

C27 C26 C25 117.0(3) . . ?

C28 C27 C26 122.3(3) . . ?

C28 C27 H27 118.9 . . ?

C26 C27 H27 118.9 . . ?

C27 C28 C29 120.9(3) . . ?

C27 C28 H28 119.6 . . ?

C29 C28 H28 119.6 . . ?

C30 C29 C28 116.9(3) . . ?

C30 C29 C37 117.8(3) . . ?

C28 C29 C37 125.3(3) . . ?

C25 C30 C29 122.3(3) . . ?

C25 C30 H30 118.9 . . ?

C29 C30 H30 118.9 . . ?

C32 C31 C36 120.4(4) . . ?

C32 C31 S2 119.3(3) . . ?

C36 C31 S2 120.3(3) . . ?

C31 C32 C33 120.8(4) . . ?

C31 C32 H32 119.6 . . ?

C33 C32 H32 119.6 . . ?

C34 C33 C32 119.0(4) . . ?

C34 C33 H33 120.5 . . ?

C32 C33 H33 120.5 . . ?

C35 C34 C33 121.0(4) . . ?

C35 C34 H34 119.5 . . ?

C33 C34 H34 119.5 . . ?

C34 C35 C36 120.6(4) . . ?

C34 C35 H35 119.7 . . ?

C36 C35 H35 119.7 . . ?

C31 C36 C35 118.1(4) . . ?

C31 C36 N4 120.2(3) . . ?

C35 C36 N4 121.7(4) . . ?

C38 C37 C29 131.9(3) . . ?

C38 C37 H37 114.0 . . ?

C29 C37 H37 114.0 . . ?

C37 C38 C39 124.9(4) . . ?

C37 C38 C40 120.6(4) . . ?

C39 C38 C40 114.5(4) . . ?

N5 C39 C38 178.3(5) . . ?

N6 C40 C38 179.6(5) . . ?

N4 C41 C42 113.7(3) . . ?

N4 C41 H41A 108.8 . . ?

C42 C41 H41A 108.8 . . ?

N4 C41 H41B 108.8 . . ?

C42 C41 H41B 108.8 . . ?

H41A C41 H41B 107.7 . . ?

C41 C42 C43 111.6(3) . . ?

C41 C42 H42A 109.3 . . ?

C43 C42 H42A 109.3 . . ?

C41 C42 H42B 109.3 . . ?

C43 C42 H42B 109.3 . . ?

H42A C42 H42B 108.0 . . ?

C44 C43 C42 114.6(3) . . ?

C44 C43 H43A 108.6 . . ?

C42 C43 H43A 108.6 . . ?

C44 C43 H43B 108.6 . . ?

C42 C43 H43B 108.6 . . ?

H43A C43 H43B 107.6 . . ?

C45 C44 C43 115.6(3) . . ?

C45 C44 H44A 108.4 . . ?

C43 C44 H44A 108.4 . . ?

C45 C44 H44B 108.4 . . ?

C43 C44 H44B 108.4 . . ?

H44A C44 H44B 107.4 . . ?

C46 C45 C44 115.4(3) . . ?

C46 C45 H45A 108.4 . . ?

C44 C45 H45A 108.4 . . ?

C46 C45 H45B 108.4 . . ?

C44 C45 H45B 108.4 . . ?

H45A C45 H45B 107.5 . . ?

C47 C46 C45 116.6(3) . . ?

C47 C46 H46A 108.1 . . ?

C45 C46 H46A 108.1 . . ?

C47 C46 H46B 108.1 . . ?

C45 C46 H46B 108.1 . . ?

H46A C46 H46B 107.3 . . ?

C46 C47 C48 116.4(4) . . ?

C46 C47 H47A 108.2 . . ?

C48 C47 H47A 108.2 . . ?

C46 C47 H47B 108.2 . . ?

C48 C47 H47B 108.2 . . ?

H47A C47 H47B 107.3 . . ?

C47 C48 H48A 109.5 . . ?

C47 C48 H48B 109.5 . . ?

H48A C48 H48B 109.5 . . ?

C47 C48 H48C 109.5 . . ?

H48A C48 H48C 109.5 . . ?

H48B C48 H48C 109.5 . . ?

C54 C49 C50 120.6(3) . . ?

C54 C49 S3 117.8(3) . . ?

C50 C49 S3 121.2(3) . . ?

C20 C049 C22 116.1(3) . . ?

C20 C049 H04A 108.3 . . ?

C22 C049 H04A 108.3 . . ?

C20 C049 H04B 108.3 . . ?

C22 C049 H04B 108.3 . . ?

H04A C049 H04B 107.4 . . ?

N7 C50 C51 121.4(3) . . ?

N7 C50 C49 122.4(3) . . ?

C51 C50 C49 116.2(3) . . ?

C52 C51 C50 122.6(4) . . ?

C52 C51 H51 118.7 . . ?

C50 C51 H51 118.7 . . ?

C51 C52 C53 121.3(4) . . ?

C51 C52 H52 119.4 . . ?

C53 C52 H52 119.4 . . ?

C52 C53 C54 116.4(4) . . ?

C52 C53 C61 124.8(3) . . ?

C54 C53 C61 118.7(3) . . ?

C49 C54 C53 122.7(3) . . ?

C49 C54 H54 118.6 . . ?

C53 C54 H54 118.6 . . ?

C56 C55 C60 120.2(4) . . ?

C56 C55 S3 117.4(3) . . ?

C60 C55 S3 122.4(3) . . ?

C57 C56 C55 121.9(4) . . ?

C57 C56 H56 119.0 . . ?

C55 C56 H56 119.0 . . ?

C56 C57 C58 118.2(4) . . ?

C56 C57 H57 120.9 . . ?

C58 C57 H57 120.9 . . ?

C59 C58 C57 121.1(5) . . ?

C59 C58 H58 119.4 . . ?

C57 C58 H58 119.4 . . ?

C58 C59 C60 121.6(4) . . ?

C58 C59 H59 119.2 . . ?

C60 C59 H59 119.2 . . ?

C59 C60 C55 117.0(4) . . ?

C59 C60 N7 121.5(4) . . ?

C55 C60 N7 121.5(3) . . ?

C62 C61 C53 130.5(4) . . ?

C62 C61 H61 114.7 . . ?

C53 C61 H61 114.7 . . ?

C61 C62 C63 125.8(4) . . ?

C61 C62 C64 119.5(4) . . ?

C63 C62 C64 114.7(4) . . ?

N8 C63 C62 177.6(5) . . ?

N9 C64 C62 178.5(5) . . ?

N7 C65 C66 116.6(3) . . ?

N7 C65 H65A 108.1 . . ?

C66 C65 H65A 108.1 . . ?

N7 C65 H65B 108.1 . . ?

C66 C65 H65B 108.1 . . ?

H65A C65 H65B 107.3 . . ?

C65 C66 C67 110.3(3) . . ?

C65 C66 H66A 109.6 . . ?

C67 C66 H66A 109.6 . . ?

C65 C66 H66B 109.6 . . ?

C67 C66 H66B 109.6 . . ?

H66A C66 H66B 108.1 . . ?

C68 C67 C66 116.6(3) . . ?

C68 C67 H67A 108.1 . . ?

C66 C67 H67A 108.1 . . ?

C68 C67 H67B 108.1 . . ?

C66 C67 H67B 108.1 . . ?

H67A C67 H67B 107.3 . . ?

C67 C68 C69 113.5(4) . . ?

C67 C68 H68A 108.9 . . ?

C69 C68 H68A 108.9 . . ?

C67 C68 H68B 108.9 . . ?

C69 C68 H68B 108.9 . . ?

H68A C68 H68B 107.7 . . ?

C70 C69 C68 115.9(4) . . ?

C70 C69 H69A 108.3 . . ?

C68 C69 H69A 108.3 . . ?

C70 C69 H69B 108.3 . . ?

C68 C69 H69B 108.3 . . ?

H69A C69 H69B 107.4 . . ?

C69 C70 C71 114.0(5) . . ?

C69 C70 H70A 108.7 . . ?

C71 C70 H70A 108.7 . . ?

C69 C70 H70B 108.7 . . ?

C71 C70 H70B 108.7 . . ?

H70A C70 H70B 107.6 . . ?

C72 C71 C70 118.1(6) . . ?

C72 C71 H71A 107.8 . . ?

C70 C71 H71A 107.8 . . ?

C72 C71 H71B 107.8 . . ?

C70 C71 H71B 107.8 . . ?

H71A C71 H71B 107.1 . . ?

C71 C72 H72A 109.5 . . ?

C71 C72 H72B 109.5 . . ?

H72A C72 H72B 109.5 . . ?

C71 C72 H72C 109.5 . . ?

H72A C72 H72C 109.5 . . ?

H72B C72 H72C 109.5 . . ?

C78 C73 C74 120.9(3) . . ?

C78 C73 S4 118.1(3) . . ?

C74 C73 S4 120.6(3) . . ?

N10 C74 C73 122.1(3) . . ?

N10 C74 C75 121.2(3) . . ?

C73 C74 C75 116.6(3) . . ?

C76 C75 C74 122.0(4) . . ?

C76 C75 H75 119.0 . . ?

C74 C75 H75 119.0 . . ?

C75 C76 C77 121.5(4) . . ?

C75 C76 H76 119.2 . . ?

C77 C76 H76 119.2 . . ?

C78 C77 C76 116.4(3) . . ?

C78 C77 C85 118.5(3) . . ?

C76 C77 C85 125.0(3) . . ?

C73 C78 C77 122.3(3) . . ?

C73 C78 H78 118.9 . . ?

C77 C78 H78 118.9 . . ?

C80 C79 C84 120.2(4) . . ?

C80 C79 S4 117.7(3) . . ?

C84 C79 S4 122.0(3) . . ?

C81 C80 C79 121.9(5) . . ?

C81 C80 H80 119.0 . . ?

C79 C80 H80 119.0 . . ?

C80 C81 C82 118.2(5) . . ?

C80 C81 H81 120.9 . . ?

C82 C81 H81 120.9 . . ?

C81 C82 C83 121.1(5) . . ?

C81 C82 H82 119.4 . . ?

C83 C82 H82 119.4 . . ?

C82 C83 C84 120.7(5) . . ?

C82 C83 H83 119.6 . . ?

C84 C83 H83 119.6 . . ?

C79 C84 C83 117.8(4) . . ?

C79 C84 N10 121.8(3) . . ?

C83 C84 N10 120.4(4) . . ?

C86 C85 C77 131.2(4) . . ?

C86 C85 H85 114.4 . . ?

C77 C85 H85 114.4 . . ?

C85 C86 C87 124.7(4) . . ?

C85 C86 C88 121.1(4) . . ?

C87 C86 C88 114.1(4) . . ?

N11 C87 C86 177.8(6) . . ?

N12 C88 C86 179.1(5) . . ?

N10 C89 C90 115.9(3) . . ?

N10 C89 H89A 108.3 . . ?

C90 C89 H89A 108.3 . . ?

N10 C89 H89B 108.3 . . ?

C90 C89 H89B 108.3 . . ?

H89A C89 H89B 107.4 . . ?

C89 C90 C91 111.8(3) . . ?

C89 C90 H90A 109.3 . . ?

C91 C90 H90A 109.3 . . ?

C89 C90 H90B 109.3 . . ?

C91 C90 H90B 109.3 . . ?

H90A C90 H90B 107.9 . . ?

C92 C91 C90 115.7(3) . . ?

C92 C91 H91A 108.4 . . ?

C90 C91 H91A 108.4 . . ?

C92 C91 H91B 108.4 . . ?

C90 C91 H91B 108.4 . . ?

H91A C91 H91B 107.4 . . ?

C91 C92 C93 115.8(3) . . ?

C91 C92 H92A 108.3 . . ?

C93 C92 H92A 108.3 . . ?

C91 C92 H92B 108.3 . . ?

C93 C92 H92B 108.3 . . ?

H92A C92 H92B 107.4 . . ?

C94 C93 C92 116.7(4) . . ?

C94 C93 H93A 108.1 . . ?

C92 C93 H93A 108.1 . . ?

C94 C93 H93B 108.1 . . ?

C92 C93 H93B 108.1 . . ?

H93A C93 H93B 107.3 . . ?

C93 C94 C95 116.9(5) . . ?

C93 C94 H94A 108.1 . . ?

C95 C94 H94A 108.1 . . ?

C93 C94 H94B 108.1 . . ?

C95 C94 H94B 108.1 . . ?

H94A C94 H94B 107.3 . . ?

C96 C95 C94 119.2(8) . . ?

C96 C95 H95A 107.5 . . ?

C94 C95 H95A 107.5 . . ?

C96 C95 H95B 107.5 . . ?

C94 C95 H95B 107.5 . . ?

H95A C95 H95B 107.0 . . ?

C95 C96 H96A 109.5 . . ?

C95 C96 H96B 109.5 . . ?

H96A C96 H96B 109.5 . . ?

C95 C96 H96C 109.5 . . ?

H96A C96 H96C 109.5 . . ?

H96B C96 H96C 109.5 . . ?

loop_

_geom_torsion_atom_site_label_1

_geom_torsion_atom_site_label_2

_geom_torsion_atom_site_label_3

_geom_torsion_atom_site_label_4

_geom_torsion

_geom_torsion_site_symmetry_1

_geom_torsion_site_symmetry_2

_geom_torsion_site_symmetry_3

_geom_torsion_site_symmetry_4

_geom_torsion_publ_flag

C7 S1 C1 C6 -152.3(2) . . . . ?

C7 S1 C1 C2 34.4(3) . . . . ?

C12 N1 C2 C3 157.0(3) . . . . ?

C17 N1 C2 C3 -10.1(4) . . . . ?

C12 N1 C2 C1 -23.8(4) . . . . ?

C17 N1 C2 C1 169.1(2) . . . . ?

C6 C1 C2 N1 175.2(3) . . . . ?

S1 C1 C2 N1 -11.7(4) . . . . ?

C6 C1 C2 C3 -5.6(4) . . . . ?

S1 C1 C2 C3 167.6(2) . . . . ?

N1 C2 C3 C4 -177.5(3) . . . . ?

C1 C2 C3 C4 3.2(4) . . . . ?

C2 C3 C4 C5 0.8(5) . . . . ?

C3 C4 C5 C6 -2.6(4) . . . . ?

C3 C4 C5 C13 176.9(3) . . . . ?

C2 C1 C6 C5 4.0(4) . . . . ?

S1 C1 C6 C5 -169.2(2) . . . . ?

C4 C5 C6 C1 0.3(4) . . . . ?

C13 C5 C6 C1 -179.4(3) . . . . ?

C1 S1 C7 C8 155.2(3) . . . . ?

C1 S1 C7 C12 -31.2(3) . . . . ?

C12 C7 C8 C9 -1.1(6) . . . . ?

S1 C7 C8 C9 172.5(3) . . . . ?

C7 C8 C9 C10 1.4(6) . . . . ?

C8 C9 C10 C11 -0.1(7) . . . . ?

C9 C10 C11 C12 -1.5(6) . . . . ?

C10 C11 C12 C7 1.7(5) . . . . ?

C10 C11 C12 N1 -177.1(3) . . . . ?

C8 C7 C12 C11 -0.5(5) . . . . ?

S1 C7 C12 C11 -173.9(2) . . . . ?

C8 C7 C12 N1 178.4(3) . . . . ?

S1 C7 C12 N1 4.9(4) . . . . ?

C2 N1 C12 C11 -153.5(3) . . . . ?

C17 N1 C12 C11 13.6(4) . . . . ?

C2 N1 C12 C7 27.6(4) . . . . ?

C17 N1 C12 C7 -165.2(3) . . . . ?

C6 C5 C13 C14 179.4(3) . . . . ?

C4 C5 C13 C14 -0.1(6) . . . . ?

C5 C13 C14 C15 1.4(6) . . . . ?

C5 C13 C14 C16 -178.9(4) . . . . ?

C13 C14 C15 N2 -165(16) . . . . ?

C16 C14 C15 N2 16(16) . . . . ?

C13 C14 C16 N3 -7(85) . . . . ?

C15 C14 C16 N3 172(100) . . . . ?

C2 N1 C17 C18 82.2(4) . . . . ?

C12 N1 C17 C18 -85.4(3) . . . . ?

N1 C17 C18 C19 175.2(3) . . . . ?

C17 C18 C19 C20 176.0(3) . . . . ?

C18 C19 C20 C049 -178.6(3) . . . . ?

C049 C22 C23 C24 177.0(5) . . . . ?

C31 S2 C25 C30 149.5(3) . . . . ?

C31 S2 C25 C26 -36.7(3) . . . . ?

C36 N4 C26 C27 -146.0(3) . . . . ?

C41 N4 C26 C27 16.9(4) . . . . ?

C36 N4 C26 C25 34.4(4) . . . . ?

C41 N4 C26 C25 -162.7(3) . . . . ?

C30 C25 C26 N4 -178.0(3) . . . . ?

S2 C25 C26 N4 8.3(4) . . . . ?

C30 C25 C26 C27 2.4(4) . . . . ?

S2 C25 C26 C27 -171.3(2) . . . . ?

N4 C26 C27 C28 -178.9(3) . . . . ?

C25 C26 C27 C28 0.7(5) . . . . ?

C26 C27 C28 C29 -2.6(5) . . . . ?

C27 C28 C29 C30 1.5(5) . . . . ?

C27 C28 C29 C37 -178.5(3) . . . . ?

C26 C25 C30 C29 -3.6(5) . . . . ?

S2 C25 C30 C29 170.1(2) . . . . ?

C28 C29 C30 C25 1.6(4) . . . . ?

C37 C29 C30 C25 -178.4(3) . . . . ?

C25 S2 C31 C32 -150.3(3) . . . . ?

C25 S2 C31 C36 32.2(3) . . . . ?

C36 C31 C32 C33 1.4(5) . . . . ?

S2 C31 C32 C33 -176.1(3) . . . . ?

C31 C32 C33 C34 -3.2(6) . . . . ?

C32 C33 C34 C35 1.8(6) . . . . ?

C33 C34 C35 C36 1.6(6) . . . . ?

C32 C31 C36 C35 1.9(5) . . . . ?

S2 C31 C36 C35 179.4(2) . . . . ?

C32 C31 C36 N4 -177.0(3) . . . . ?

S2 C31 C36 N4 0.5(4) . . . . ?

C34 C35 C36 C31 -3.4(5) . . . . ?

C34 C35 C36 N4 175.5(3) . . . . ?

C26 N4 C36 C31 -39.5(4) . . . . ?

C41 N4 C36 C31 157.5(3) . . . . ?

C26 N4 C36 C35 141.7(3) . . . . ?

C41 N4 C36 C35 -21.4(5) . . . . ?

C30 C29 C37 C38 175.2(3) . . . . ?

C28 C29 C37 C38 -4.8(6) . . . . ?

C29 C37 C38 C39 -2.4(6) . . . . ?

C29 C37 C38 C40 178.6(3) . . . . ?

C37 C38 C39 N5 -161(24) . . . . ?

C40 C38 C39 N5 18(24) . . . . ?

C37 C38 C40 N6 -69(72) . . . . ?

C39 C38 C40 N6 112(72) . . . . ?

C26 N4 C41 C42 64.5(4) . . . . ?

C36 N4 C41 C42 -132.6(3) . . . . ?

N4 C41 C42 C43 175.5(3) . . . . ?

C41 C42 C43 C44 178.7(3) . . . . ?

C42 C43 C44 C45 179.3(3) . . . . ?

C43 C44 C45 C46 179.0(3) . . . . ?

C44 C45 C46 C47 179.9(4) . . . . ?

C45 C46 C47 C48 -177.7(4) . . . . ?

C55 S3 C49 C54 -160.2(3) . . . . ?

C55 S3 C49 C50 27.3(3) . . . . ?

C19 C20 C049 C22 177.4(4) . . . . ?

C23 C22 C049 C20 -176.0(4) . . . . ?

C60 N7 C50 C51 163.4(3) . . . . ?

C65 N7 C50 C51 -10.9(4) . . . . ?

C60 N7 C50 C49 -16.5(5) . . . . ?

C65 N7 C50 C49 169.2(3) . . . . ?

C54 C49 C50 N7 176.2(3) . . . . ?

S3 C49 C50 N7 -11.5(4) . . . . ?

C54 C49 C50 C51 -3.7(5) . . . . ?

S3 C49 C50 C51 168.6(2) . . . . ?

N7 C50 C51 C52 -179.4(3) . . . . ?

C49 C50 C51 C52 0.5(5) . . . . ?

C50 C51 C52 C53 2.1(5) . . . . ?

C51 C52 C53 C54 -1.4(5) . . . . ?

C51 C52 C53 C61 178.7(3) . . . . ?

C50 C49 C54 C53 4.5(5) . . . . ?

S3 C49 C54 C53 -168.0(2) . . . . ?

C52 C53 C54 C49 -1.9(5) . . . . ?

C61 C53 C54 C49 177.9(3) . . . . ?

C49 S3 C55 C56 160.0(3) . . . . ?

C49 S3 C55 C60 -22.9(3) . . . . ?

C60 C55 C56 C57 -0.4(6) . . . . ?

S3 C55 C56 C57 176.7(3) . . . . ?

C55 C56 C57 C58 0.5(6) . . . . ?

C56 C57 C58 C59 0.8(7) . . . . ?

C57 C58 C59 C60 -2.2(7) . . . . ?

C58 C59 C60 C55 2.2(5) . . . . ?

C58 C59 C60 N7 -177.9(4) . . . . ?

C56 C55 C60 C59 -0.9(5) . . . . ?

S3 C55 C60 C59 -177.9(3) . . . . ?

C56 C55 C60 N7 179.2(3) . . . . ?

S3 C55 C60 N7 2.2(4) . . . . ?

C50 N7 C60 C59 -158.4(3) . . . . ?

C65 N7 C60 C59 15.9(4) . . . . ?

C50 N7 C60 C55 21.5(5) . . . . ?

C65 N7 C60 C55 -164.2(3) . . . . ?

C52 C53 C61 C62 12.4(6) . . . . ?

C54 C53 C61 C62 -167.4(3) . . . . ?

C53 C61 C62 C63 2.3(6) . . . . ?

C53 C61 C62 C64 -178.4(4) . . . . ?

C61 C62 C63 N8 176(100) . . . . ?

C64 C62 C63 N8 -3(13) . . . . ?

C61 C62 C64 N9 136(20) . . . . ?

C63 C62 C64 N9 -44(20) . . . . ?

C50 N7 C65 C66 84.4(4) . . . . ?

C60 N7 C65 C66 -90.2(4) . . . . ?

N7 C65 C66 C67 178.9(3) . . . . ?

C65 C66 C67 C68 168.4(4) . . . . ?

C66 C67 C68 C69 -173.6(4) . . . . ?

C67 C68 C69 C70 179.1(5) . . . . ?

C68 C69 C70 C71 -179.1(5) . . . . ?

C69 C70 C71 C72 71.5(7) . . . . ?

C79 S4 C73 C78 -155.4(3) . . . . ?

C79 S4 C73 C74 31.6(3) . . . . ?

C84 N10 C74 C73 -17.7(4) . . . . ?

C89 N10 C74 C73 172.3(3) . . . . ?

C84 N10 C74 C75 161.9(3) . . . . ?

C89 N10 C74 C75 -8.1(4) . . . . ?

C78 C73 C74 N10 173.2(3) . . . . ?

S4 C73 C74 N10 -14.0(4) . . . . ?

C78 C73 C74 C75 -6.4(4) . . . . ?

S4 C73 C74 C75 166.4(2) . . . . ?

N10 C74 C75 C76 -175.7(3) . . . . ?

C73 C74 C75 C76 3.9(5) . . . . ?

C74 C75 C76 C77 0.8(5) . . . . ?

C75 C76 C77 C78 -3.0(5) . . . . ?

C75 C76 C77 C85 174.3(3) . . . . ?

C74 C73 C78 C77 4.4(5) . . . . ?

S4 C73 C78 C77 -168.6(2) . . . . ?

C76 C77 C78 C73 0.4(5) . . . . ?

C85 C77 C78 C73 -177.0(3) . . . . ?

C73 S4 C79 C80 156.8(3) . . . . ?

C73 S4 C79 C84 -25.9(3) . . . . ?

C84 C79 C80 C81 -1.5(6) . . . . ?

S4 C79 C80 C81 175.9(4) . . . . ?

C79 C80 C81 C82 2.2(7) . . . . ?

C80 C81 C82 C83 -1.1(8) . . . . ?

C81 C82 C83 C84 -0.7(7) . . . . ?

C80 C79 C84 C83 -0.3(5) . . . . ?

S4 C79 C84 C83 -177.6(3) . . . . ?

C80 C79 C84 N10 179.4(3) . . . . ?

S4 C79 C84 N10 2.1(5) . . . . ?

C82 C83 C84 C79 1.4(6) . . . . ?

C82 C83 C84 N10 -178.3(4) . . . . ?

C74 N10 C84 C79 24.2(5) . . . . ?

C89 N10 C84 C79 -165.8(3) . . . . ?

C74 N10 C84 C83 -156.1(3) . . . . ?

C89 N10 C84 C83 13.9(4) . . . . ?

C78 C77 C85 C86 -173.5(3) . . . . ?

C76 C77 C85 C86 9.3(6) . . . . ?

C77 C85 C86 C87 3.6(7) . . . . ?

C77 C85 C86 C88 -177.2(3) . . . . ?

C85 C86 C87 N11 131(16) . . . . ?

C88 C86 C87 N11 -48(17) . . . . ?

C85 C86 C88 N12 150(38) . . . . ?

C87 C86 C88 N12 -31(38) . . . . ?

C74 N10 C89 C90 82.6(4) . . . . ?

C84 N10 C89 C90 -87.8(4) . . . . ?

N10 C89 C90 C91 178.5(3) . . . . ?

C89 C90 C91 C92 171.3(3) . . . . ?

C90 C91 C92 C93 -179.3(4) . . . . ?

C91 C92 C93 C94 -179.6(4) . . . . ?

C92 C93 C94 C95 179.9(6) . . . . ?

C93 C94 C95 C96 93.0(10) . . . . ?

loop_

_geom_hbond_atom_site_label_D

_geom_hbond_atom_site_label_H

_geom_hbond_atom_site_label_A

_geom_hbond_distance_DH

_geom_hbond_distance_HA

_geom_hbond_distance_DA

_geom_hbond_angle_DHA

_geom_hbond_site_symmetry_A

C3 H3 N5 0.93 2.46 3.300(6) 150.0 2_666

C27 H27 N2 0.93 2.56 3.408(6) 152.7 2_666

C51 H51 N11 0.93 2.60 3.379(6) 142.0 2_666

C75 H75 N8 0.93 2.60 3.427(6) 148.9 2_666

_diffrn_measured_fraction_theta_max 0.869

_diffrn_reflns_theta_full 26.00

_diffrn_measured_fraction_theta_full 0.998

_refine_diff_density_max 0.362

_refine_diff_density_min -0.291

_refine_diff_density_rms 0.042

**Cartesian co-ordinates of optimized geometry at B3LYP/6-31G (d, p) (3a)**

S -0.00164800 -2.62271700 -1.66367200

N 1.36432900 -0.87287400 0.35544600

N -4.50193100 2.98364300 1.75606300

N -7.55856200 0.93177300 -0.63774600

C -0.79173800 -1.38571900 -0.65438800

C 0.00289200 -0.59245800 0.20587900

C -0.62678800 0.46556500 0.89338100

H -0.05257200 1.08358200 1.57250300

C -1.97597400 0.73356200 0.73044800

H -2.40892800 1.55814800 1.28148400

C -2.77706000 -0.06301900 -0.11640300

C -2.14778800 -1.13654200 -0.78843900

H -2.73411200 -1.77061300 -1.44744100

C 1.20816000 -3.17232300 -0.47511500

C 1.60217100 -4.51018400 -0.44348700

H 1.12886700 -5.21664500 -1.11829500

C 2.60100000 -4.92952800 0.43502700

H 2.91799200 -5.96726800 0.44104400

C 3.16950000 -4.00871800 1.31258100

H 3.92920000 -4.32435700 2.02085900

C 2.75315500 -2.67718100 1.30943400

H 3.17706100 -1.98948000 2.03192700

C 1.78727500 -2.23103100 0.39526000

C -4.18923200 0.12251700 -0.35389100

H -4.63003200 -0.60380200 -1.03274400

C -5.07789200 1.05197300 0.12211900

C -4.75396800 2.11445000 1.02324600

C -6.44765200 0.98852000 -0.29478400

C 2.27809300 0.18338800 0.81751600

H 2.02430600 0.51620200 1.83464100

H 3.27116300 -0.26275600 0.87598700

C 2.34783100 1.37693000 -0.14494200

H 2.65161900 1.00550500 -1.13100400

H 1.35568100 1.82277000 -0.27398200

C 3.33046600 2.45042700 0.34063400

H 4.32815800 2.00614400 0.46657500

H 3.02671000 2.80189400 1.33709100

C 3.42730100 3.65034400 -0.60951000

H 2.42954900 4.09351400 -0.73639000

H 3.73214600 3.30058500 -1.60583000

C 4.40429800 4.73167600 -0.13157400

H 5.40134900 4.28817300 -0.00520500

H 4.09898500 5.07983000 0.86450000

C 4.49360700 5.92667500 -1.08504700

H 3.51850000 6.41207700 -1.20453500

H 4.83128400 5.61597300 -2.08019700

H 5.19664500 6.68025900 -0.71624300

**Cartesian co-ordinates of optimized geometry at B3LYP/6-31G (d, p) (3b)**

S -2.29686200 2.16673400 1.75744100

N -0.23933200 1.72824500 -0.38180500

N -2.42158000 -4.92993600 -1.85099500

N -5.96246000 -5.33652200 0.71334900

C -2.17633600 0.73230600 0.70793100

C -1.10414000 0.64320500 -0.21036200

C -0.95377400 -0.55926900 -0.93146000

H -0.15069000 -0.65793200 -1.65126200

C -1.81980200 -1.62503500 -0.75048100

H -1.65925400 -2.52487600 -1.32976000

C -2.90254500 -1.53548100 0.15126800

C -3.06052800 -0.32282500 0.86167400

H -3.88219900 -0.21968200 1.56478300

C -1.77514200 3.38536900 0.56572300

C -2.32168900 4.66867700 0.58966600

H -3.09953000 4.89859300 1.31115800

C -1.86338200 5.64582400 -0.29377400

H -2.27735400 6.64817300 -0.25674100

C -0.88691300 5.31556700 -1.23113000

H -0.53856600 6.05716300 -1.94325500

C -0.36315400 4.02355400 -1.28268000

H 0.36176900 3.77731200 -2.04972000

C -0.77692300 3.04597400 -0.36570900

C -3.86930200 -2.57684600 0.40782500

H -4.63400600 -2.31075400 1.13377900

C -3.99914900 -3.84367900 -0.10054600

C -3.12601000 -4.43662700 -1.06584100

C -5.08167000 -4.66973400 0.34650400

C 1.10915200 1.50402800 -0.92539300

H 1.06650700 1.10313800 -1.94879100

H 1.58774500 2.48105900 -0.99506200

C 1.97664200 0.61235600 -0.02610800

H 2.03285300 1.07655400 0.96584100

H 1.50310500 -0.36508500 0.11570000

C 3.38670200 0.41855600 -0.59845100

H 3.86239100 1.39894700 -0.74369400

H 3.31813700 -0.03639700 -1.59695400

C 4.28266400 -0.45129700 0.29232300

H 3.80616700 -1.43067800 0.43877500

H 4.35177200 0.00416000 1.29007100

C 5.69394800 -0.65317900 -0.27259200

H 6.16889300 0.32705300 -0.42166500

H 5.62359800 -1.11009800 -1.27001400

C 6.59082900 -1.52007900 0.61930700

H 6.11581000 -2.50006500 0.76956300

H 6.66195800 -1.06308900 1.61676800

C 8.00257300 -1.72470800 0.05608600

H 8.47683300 -0.74538600 -0.09495800

H 7.93134400 -2.18303500 -0.93968300

C 8.89180300 -2.59003100 0.95394000

H 9.89053400 -2.71845200 0.52438700

H 9.01200900 -2.13925500 1.94561800

H 8.46043000 -3.58739600 1.09589000
